# Derivatives of benzo-1,4-thiazine-3-carboxylic acid and the corresponding amino acid conjugates

**DOI:** 10.3762/bjoc.18.124

**Published:** 2022-09-09

**Authors:** Péter Kisszékelyi, Tibor Peňaška, Klára Stankovianska, Mária Mečiarová, Radovan Šebesta

**Affiliations:** 1 Department of Organic Chemistry, Faculty of Natural Sciences, Comenius University in Bratislava, Mlynská dolina, Ilkovičova 6, 842 15 Bratislava, Slovakia

**Keywords:** amino acid, benzothiazine, oxidative dimerization, peptide coupling, stereoselective hydrogenation

## Abstract

Herein, we present the synthesis and utilization of derivatives of 4*H*-benzo[*b*][1,4]thiazine-3-carboxylic acid. These benzothiazine compounds were assembled via the coupling of aminothiols and bromopyruvates. Oxidative dimerization of these starting materials was also observed and the corresponding benzothiazine dimers were isolated. Moreover, the coupling of benzothiazines with amino acids was realized. In doing so, an enantioselective synthesis of the nonproteinogenic amino acid 2-amino-3-propylhexanoic acid was accomplished.

## Introduction

Heterocyclic compounds with a benzothiazine moiety are attractive building blocks in medicinal chemistry. Benzo-1,4-thiazine derivatives possess a wide range of biological and pharmacological properties, such as anticancer and antitumor, antioxidant, antimicrobial, antibacterial, antifungal, antiviral, antimalarial, antidiabetic, antihypertensive, anti-inflammatory, analgesic, anti-rheumatic, or anti-allergic properties [1–5].

**Figure 1:**
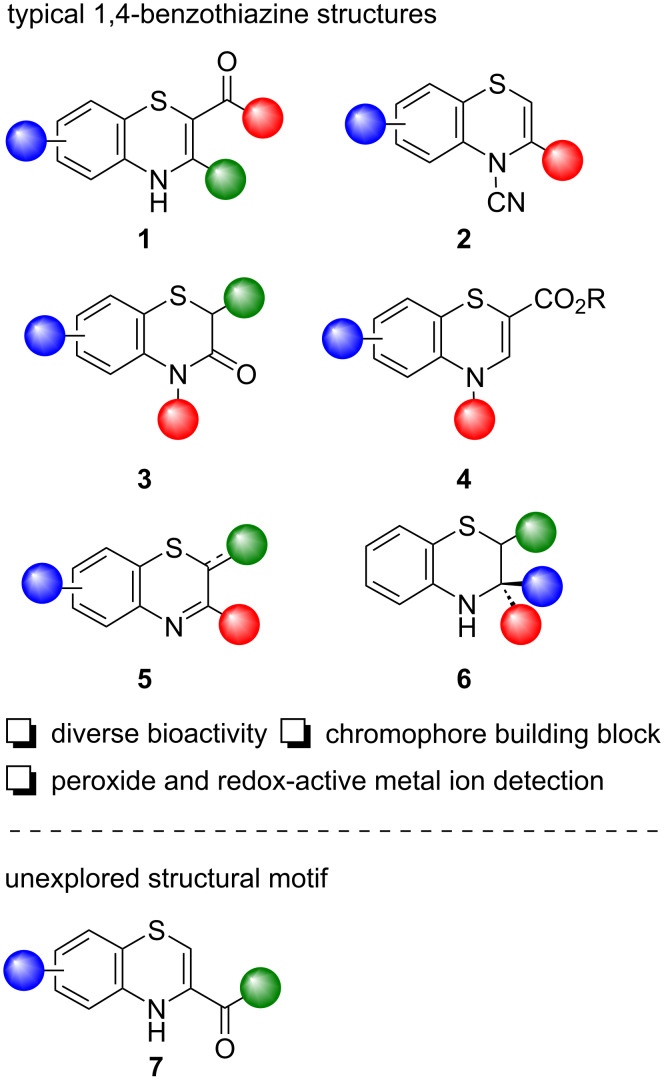
Typical benzothiazine structures.

Several methods for the preparation of 4*H*-benzo-1,4-thiazines have been described in the literature. Methods for the synthesis of 2,3-disubstituted 4*H*-benzo-1,4-thiazines **1** (Figure 1) are the most studied and described. Such benzothiazine derivatives are typically prepared by reactions of various carbonyl or carboxyl compounds with 2-aminothiophenols [6–7] or the respective dimers (2,2'-disulfanediyldianilines) [8]. These disulfides are often formed in situ from the corresponding aminothiols [9–13].

Green chemistry methods for benzo-1,4-thiazine synthesis have also been described in the literature. 2,3-Disubstituted-1,4-benzothiazines were prepared in high yield (83–96%) by oxidative cyclocondensation of 2-aminobenzenethiols and 1,3-dicarbonyl compounds using a catalytic amount of hydrazine hydrate without solvent in a short reaction time (10 min) [14]. Reactions of 2-aminothiophenols with β-keto esters and β-diketones under microwave irradiation (MWI) using basic alumina as heterogeneous catalyst without solvent afforded 4*H*-benzo-1,4-thiazines in a yield of 69–85% within 6–11 min [15]. Furthermore, baker’s yeast as whole-cell biocatalyst catalyzed the reaction of 2-aminothiophenols with 1,3-dicarbonyl compounds in methanol and the corresponding 4*H*-benzo-1,4-thiazines were prepared in 51–82% yield. These reactions were significantly accelerated by ultrasonic irradiation [16]. Cyclocondensation of 1,3-dicarbonyl compounds with substituted diaryl disulfides in water in the presence of β-cyclodextrin gave 2,3-disubstituted benzo-1,4-thiazines in 70–91% yield in 50 min [17]. A highly efficient visible-light-mediated one-pot, three-component procedure was also explored for the preparation of 3-aryl-4*H*-benzo-1,4-thiazin-2-amines [18].

3-Aryl- and 3-alkyl-4*H*-benzo[*b*][1,4]thiazine-4-carbonitriles **2** (Figure 1) were synthesized in high yield from the corresponding 2-aminobenzothiazoles using the copper–organic framework Cu–MOF-74 as a catalyst [19]. The reactions of 2-aminobenzenethioles with ethyl 2-bromoalkanoates [20], 2-chloroacetic acid [21–22], or diethyl 2-bromo-2-methylmalonate [23] gave 2*H*-benzo[*b*][1,4]thiazin-3(4*H*)-one derivatives **3** (Figure 1), which also have interesting biological properties. 2*H*-Benzo-1,4-thiazin-3(4*H*)-ones were also prepared by cyclization of 1,2-diaryldisulfanes with dialkyl but-2-ynedioates [24–25]. *N*-Substituted benzo-1,4-thiazine-2-carboxylates **4** (Figure 1) were prepared by *m*-CPBA-mediated oxidative ring expansion of substituted benzothiazoles [26], or via copper-catalyzed intramolecular amination of aryl bromides [27].

Recently, Nguyen and Retailleau introduced a TFA-catalyzed umpolung strategy with 2-aminothiophenols, preparing several 2*H*-benzo-1,4-thiazine derivatives **5** in high yield [28]. 3-Phenyl-2*H*-benzo-1,4-thiazine, an earlier representative of this family, was found to transform into a green-blue chromophore in the presence of peroxides or redox-active metal ions under acidic conditions, creating a potential detection method for such entities [29]. Additionally, the same structure was used for the preparation of a benzo-1,4-thiazine-based cyanine chromophore, which showed a reversible acidochromic behavior [30]. Zhao et al. demonstrated a three-component transition-metal-free aerobic method using a KI/DMSO/O_2_ system for the facile generation of iminobenzo-1,4-thiazines in moderate to good yield [31]. 3,4-Dihydro-2*H*-benzo-1,4-thiazine derivatives **6** were also successfully prepared [32]. The protocol, using NaI as a catalyst and K_2_S_2_O_8_ as an oxidant, tolerated a broad range of substrates with good stereoselectivity.

Interestingly, structurally related benzothiazine derivatives with a carboxylic function in the C-3 position are only seldomly described in the literature. Syntheses and utilization of the corresponding 4*H*-benzo[*b*][1,4]thiazine-3-carboxylic acids **7** are very rare.

Part of our research program is the construction of chiral heterocyclic compounds of medicinal interest [33–34]. Recently, we have been involved in the synthesis of potential SARS-CoV-2 protease inhibitors. Given the potential usefulness of the benzothiazine scaffold as a biologically active unit and the peptidomimetic nature of many SARS-CoV-2 protease inhibitors [35], we decided to investigate the viability of attaching a 4*H*-benzo[*b*][1,4]thiazine-3-carboxylic core to amino acids.

## Results and Discussion

**Scheme 1:**
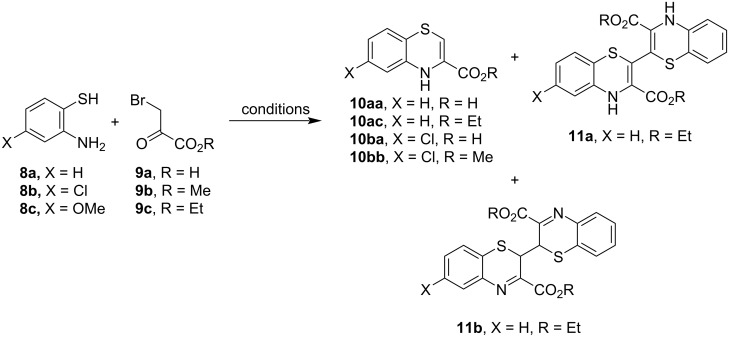
Condensation reactions of 2-aminothiophenols **8** and bromopyruvic acid and esters **9**.

We began our work with the condensation reactions of 2-aminothiophenols **8** and bromopyruvic acid and esters **9** to form 4*H*-benzo-1,4-thiazines **10**, having a carboxylic acid or an ester function at the C-3 position (Scheme 1).

The reaction of thiol **8a** with bromo-substituted acid **9a** in diethyl ether at 0 °C for 1 h gave acid **10aa** in 75% yield, while complete decomposition was observed at reflux temperature after only 10 min (Table 1, entries 1 and 2). Acid **10aa** did not form when the reaction was performed in ethanol, CH_2_Cl_2_, THF, DMF, or ethyl acetate at different temperatures under classical conditions as well as under MWI. The acid **10aa** could not be formed even when 2,2'-disulfanediyldianiline was used as the starting material in DMF or ethanol at room temperature or under reflux. Thiol **8a** reacted with the keto ester **9c** in ethanol to form the ester **10ac** with a yield of 51% and 29%, respectively, under different conditions (Table 1, entries 3 and 4). Reactions in ethanol under MWI and in CH_2_Cl_2_ with classical stirring at room temperature only resulted in oxidative dimerization, forming derivative **11b** in 15–28% yield (Table 1, entries 5–7). Neither did the reaction proceed in ethyl acetate nor in CH_2_Cl_2_. Like acid **9a**, 2,2'-disulfanediyldianiline did also not react with the ester **9c**. The reaction of 2-amino-4-chlorobenzenethiol (**8b**) with keto acid **9a** in ethanol provided acid **10ba** in 43% and 66% yield, depending on the reaction time (Table 1, entries 8 and 9). Similarly, ester **10bb** was formed in 50% yield in ethanol and 30% in diethyl ether (Table 1, entries 10 and 11). Reactions with thiol **8c**, having an electron-donating methoxy group, and acid **9a** or esters **9b**,**c** only gave unidentifiable decomposition products in various solvents (ethanol, diethyl ether, methanol, and CH_2_Cl_2_) at different temperatures under classical conditions and even under MWI (Table 1, entries 12 and 13). Also, reactions with 6,6'-disulfanediylbis(3-methoxyaniline) were unsuccessful.

**Table 1:** Conditions applied for the condensation reactions.^a^

entry	X	R	conditions	yield of **10** (%)	yield of **11b** (%)

1	H	H	Et_2_O, 0 °C, 1 h	75 (**10aa**)	—
2	H	H	Et_2_O, reflux, 10 min	decomposition	
3	H	Et	EtOH, rt, 40 min	51 (**10ac**)	—
4	H	Et	EtOH, 0 °C → rt, 2 h	29 (**10ac**)	—
5	H	Et	EtOH, MWI, 100 °C, 20 min	—	28
6	H	Et	EtOH, MWI, 60 °C, 1 h	—	20
7	H	Et	CH_2_Cl_2_, rt, 2 h	—	15
8	Cl	H	EtOH, rt, 0.5 h	43 (**10ba**)	—
9	Cl	H	EtOH, rt, 1 h	66 (**10ba**)	—
10	Cl	Me	EtOH, rt, 0.5 h	50 (**10bb**)	—
11	Cl	Me	Et_2_O, rt, 1 h	30 (**10bb**)	—
12	MeO	H	Et_2_O, 0 °C, 1 h	decomposition	
13	MeO	Et	EtOH, 0 °C → rt, 1 h	decomposition	

^a^Refer to Supporting Information File 1 for all explored reaction conditions.

**Scheme 2:**
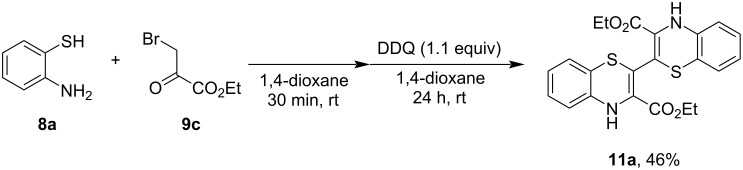
Direct synthesis of dimer **11a** under oxidative reaction conditions.

Having observed dimer formation during the syntheses of benzothiazines, we have attempted to synthesize the corresponding dimer directly. To that end, we attempted reactions from aminothiol **8a** as well as from the corresponding disulfide, both at room temperature and under MWI. In these experiments, the yield of dimer **11a** was in the range of 10–34%. We also tried to enhance the oxidative dimerization using a mild oxidizing agent. The use of 2,3-dichloro-5,6-dicyano-1,4-benzoquinone (DDQ) in 1,4-dioxane afforded the dimer **11a** in a slightly better yield of 46% (Scheme 2).

For all the prepared benzothiazine derivatives **10** we observed some degree of instability. The derivatives were reasonably stable in the solid state but usually decomposed in solution. During the preparation of **10ac**, dimers **11a** and **11b** were also detected in most experiments (TLC analysis), and they could be isolated by crystallization from MeOH or hexane. Performing the reaction in the dark with freshly degassed ethanol at lower temperature did bring about somewhat better results, but the oxidative dimerization was still a competitive reaction. NMR analyses of the isolated product **10ac** also revealed the presence of the corresponding 2*H*-isomer in a low amount, but it was not isolated separately.

**Figure 2:**
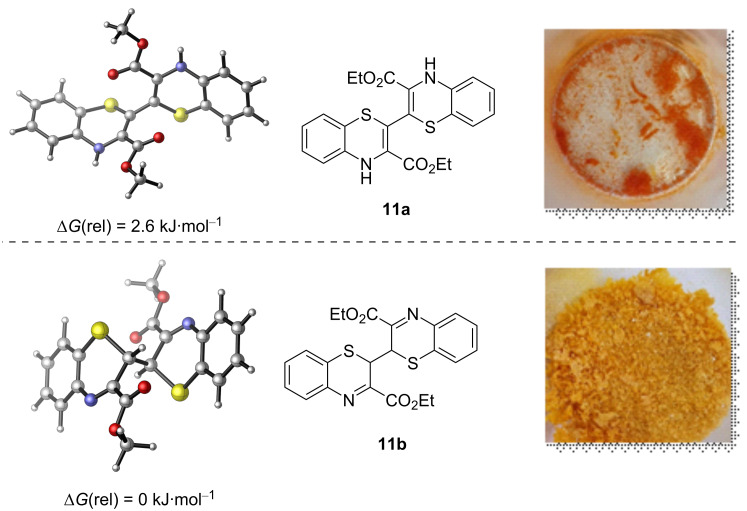
DFT (ωB97xD/6-31G*)-calculated structures of enamine and imine tautomers **11a** and **11b**.

Quantum chemical calculations (ωB97xD/6-31G(d)//MN15/6-311+G(2d,p)) revealed that the stability of the two isomers was very similar, with a difference in Δ*G* of only 2.6 kJ⋅mol^−1^ and 2*H*-benzo-1,4-thiazine **11b** being more stable (Figure 2). It seems likely that initially enamine dimer **11a** formed, which then tautomerized to the more stable imine form **11b**.

**Scheme 3:**
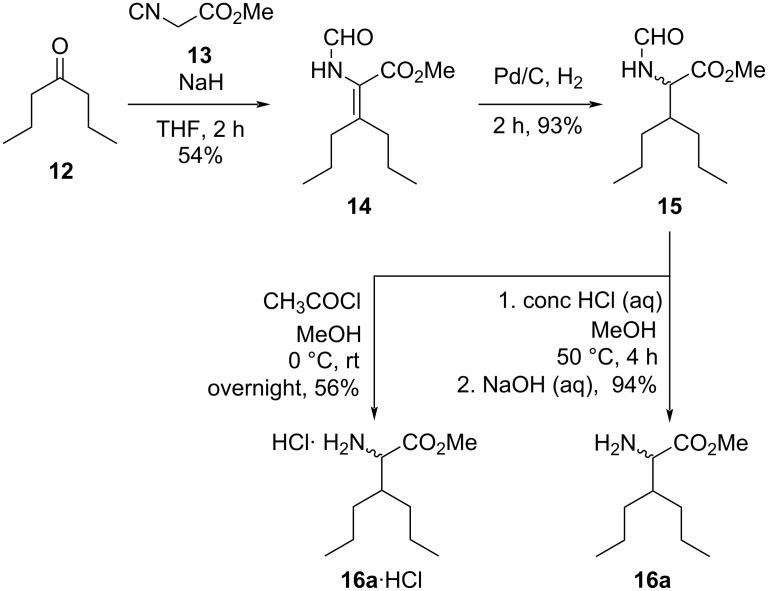
Preparation of racemic 3-propylnorleucin **16a**.

As a continuation of our work, we aimed to utilize the carboxylic acid function of the prepared 4*H*-benzo-1,4-thiazines **10** by attaching them to nonproteinogenic amino acid **16a** and ʟ-phenylalanine. Preparation of 3-propylnorleucin methyl ester (**16a**) started with the condensation reaction of heptan-4-one (**12**) and methyl isocyanoacetate (**13**). The palladium-catalyzed hydrogenation of intermediate **14** gave the racemic *N*-formyl-protected amino acid methyl ester **15** in good yield. Using either concentrated HCl (aq) or in situ-formed HCl from the reaction of MeOH and acetyl chloride, compound **15** could easily be deprotected to gain either the salt **16a**·HCl or the free amine **16a** in good to excellent yield (Scheme 3).

We also explored the asymmetric catalytic hydrogenation of adduct **14**. Our first attempt at the reduction using organocatalyzed transfer hydrogenation was unsuccessful (see Supporting Information File 1). The (*R*)-Ru(OAc)_2_(BINAP)-assisted hydrogenation with H_2_ pressure up to 50 bar was also found to be ineffective. By changing the metal complex to Rh(COD)_2_BF_4_, we successfully realized the saturation of the double bond. Chiral ligands (*R*)-BINAP (**L1**) and (*R*,*R*)-phenyl-BPE (**L4**) gave unsatisfactory selectivity (Table 2, entries 1 and 4). Application of ligand (*S*,*S*)-methyl-DUPHOS (**L3**) gave increased ee in the hydrogenation reaction, but the best result (90% ee) was achieved using 6 mol % Josiphos ligand **L2** at 35 °C.

**Table 2:** Stereoselective catalytic hydrogenation reactions of dehydroamino acid ester **14**.

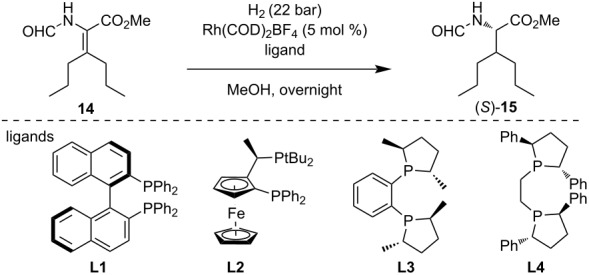				

entry	ligand (mol %)	*T* (°C)	yield^a^	ee (%)

1	**L1** (5)^b^	40	not determined	21
2	**L2** (5)	40	90	83
3	**L3** (5)	40	85	73
4	**L4** (5)	40	87	19
5	**L2** (6)	35	90	90
6	**L3** (6)	35	83	69

^a^Isolated yield after purification by flash chromatography. ^b^No complete conversion.

**Scheme 4:**
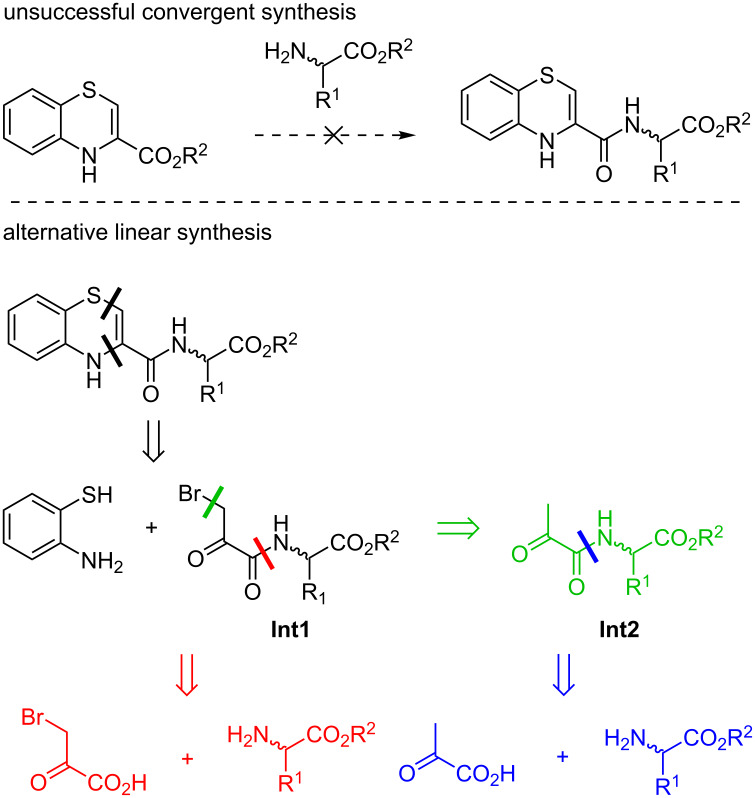
Unsuccessful direct coupling of amino acid methyl esters with the benzothiazine motif and retrosynthetic analysis of alternative linear reaction routes.

Following the synthesis of 3-propylnorleucin methyl ester (**16a**), we carried on with the amine couplings. Several attempts were made to combine the benzothiazine motif with amino acid methyl esters (Scheme 4) using coupling agents (EDC, COMU, T_3_P^®^, etc.) or through acid chloride (SOCl_2_ and (COCl)_2_) in a flask with stirring or under ball-milling conditions. However, we were unable to isolate the desired amides (see Supporting Information File 1 for detailed reaction conditions) since decomposition of the benzothiazine core was observed in all coupling reactions.

**Scheme 5:**
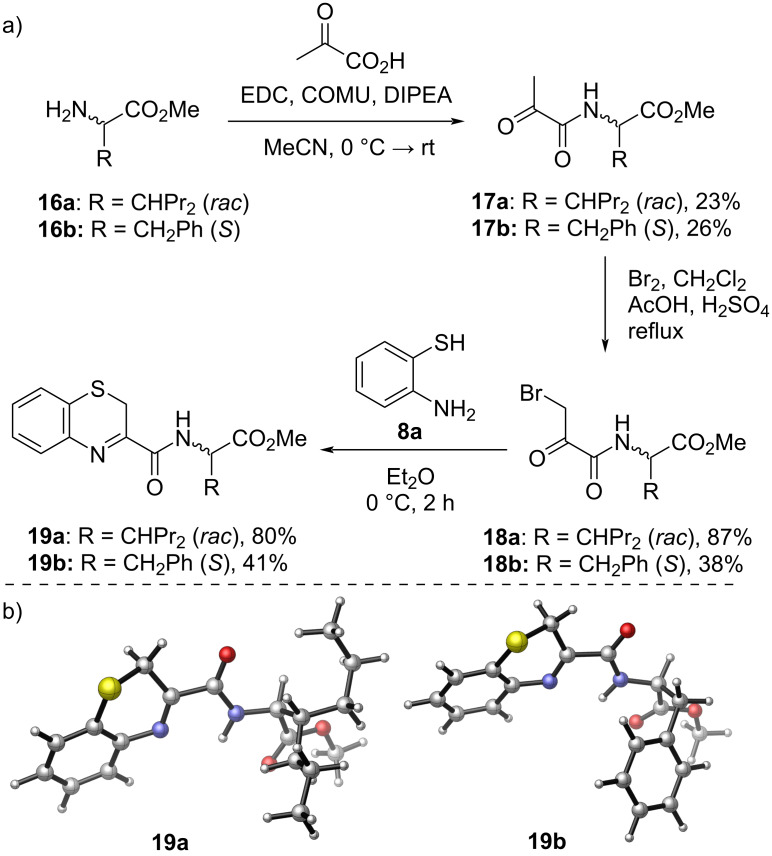
a) Synthesis of 2*H*-benzo-1,4-thiazine amino acid conjugates **19a** and **19b** and b) 3D renderings of **19a** and **19b** obtained by DFT calculations (ωB97xD/6-31G*).

To avoid the issues related to the instable benzothiazine ring, we explored other possible linear reaction routes (Scheme 4) as an alternative to the original convergent synthesis plan. Instead of the classical amide coupling, bromo derivative **Int1** should allow the formation of the benzothiazine ring as the final step. As an α-halocarbonyl compound, we tried to prepare **Int1** through the coupling of bromopyruvic acid and ester, respectively, with the amino acids. However, these reactions proved to be unsuccessful due to the high reactivity of the bromo derivative. On the contrary, coupling of the less reactive amides formed from pyruvic acid and the amino acids **16a** and **16b** was accomplished (Scheme 5a), and several different conditions were tested (see Supporting Information File 1). Next, compounds **17a** and **17b** were brominated in the α-position using Br_2_ under acidic conditions. Finally, cyclization reaction with 2-aminothiophenol (**8a**) in dry diethyl ether at 0 °C gave benzo-1,4-thiazines **19a** and **19b** in good yield. Interestingly, the isolated products were not the expected 4*H*-, rather the 2*H*-benzo-1,4-thiazines. 3D renderings of derivatives **19a** and **19b** obtained at the ωB97xD/6-31G* level are depicted in Scheme 5b.

## Conclusion

In conclusion, we have described the synthesis of rarely explored 4*H*-benzo[*b*][1,4]thiazine-3-carboxylic esters and amides with amino acids. Benzothiazine derivatives with a carboxylic function in the C-3 position exhibit low stability under acidic as well as basic conditions, which complicates the synthetic utilization. As such, direct coupling of 4*H*-benzo[*b*][1,4]thiazine-3-carboxylic acid with amino acids failed. However, we have managed the synthesis of benzothiazine–amino acid conjugates via a linear synthesis in which the benzothiazine moiety was assembled from pyruvic acid attached to an amino acid. Oxidative dimerization of benzothiazine derivatives was also observed, and potentially useful benzothiazine dimers were isolated.

## Supporting Information

File 1 Experimental procedures and characterization data, additional experimental results, pictures of NMR and HRMS spectra.
