# Supplementary material for: Derivatives of benzo-1,4-thiazine-3-carboxylic acid and the corresponding amino acid conjugates
Source: Beilstein J Org Chem. 2022 Sep 9;18:1195–202. doi: 10.3762/bjoc.18.124 (PMC9475188; doi:10.3762/bjoc.18.124)
Supplement: File 1 — Experimental procedures and characterization data, additional experimental results, pictures of NMR and HRMS spectra. [file Beilstein_J_Org_Chem-18-1195-s001.pdf]

BEILSTEIN JOURNAL OF ORGANIC CHEMISTRY

Supporting Information

for

Derivatives of benzo-1,4-thiazine-3-carboxylic acid and the corresponding amino acid conjugates

Péter Kisszékelyi, Tibor Peňaška, Klára Stankovianska, Mária Mečiarová and Radovan Šebesta

Beilstein J. Org. Chem. **2022,** *18,* 1195–1202. doi:10.3762/bjoc.18.124

Experimental procedures and characterization data, additional experimental results, pictures of NMR and HRMS spectra

License and Terms: This is a supporting information file under the terms of the Creative Commons Attribution License (https://creativecommons.org/licenses/by/4.0). Please note that the reuse, redistribution and reproduction in particular requires that the author(s) and source are credited and that individual graphics may be subject to special legal provisions.
The license is subject to the *Beilstein Journal of Organic Chemistry* terms and conditions: (https://www.beilstein-journals.org/bjoc/terms)

Table of contents

1. General information S2
2. Additional experimental results S3
3. Experimental procedures S9
4. Characterization data S15
5. Pictures of NMR, MS, HRMS, and HPLC spectra S22
6. Stereochemical description of **16**·HCl S61
7. Details of DFT calculations S62
8. References S69

1. General information

Chemicals were purchased from Merck (previously Sigma Aldrich), Alfa Aesar, Acros Organics and TCI, and if not otherwise noted, they were used as provided. Reactions were monitored by thin-layer chromatography (TLC) analysis using Merck silica gel 60 F_{254} plates. Following the general workup, products were purified by flash chromatography using Isolera Biotage FSKO-1107-0010 or Büchi Pure C-810 Flash system and Std, HC or HP Puriflash columns by Interchim. NMR spectra were measured on a Varian VNMRS 600 spectrometer (600 MHz for ^{1}H NMR experiments, 150 MHz for ^{13}C NMR spectra) and Bruker Avance NEO 400 MHz (400 MHz for ^{1}H NMR and 100 MHz for ^{13}C NMR). Chemical shifts (δ) were referenced to TMS as an internal standard and are given in ppm. Interaction constants (*J*) are in Hz. The following abbreviations are used to describe the multiplicity of observed signals: s (singlet), d (doublet), t (triplet), q (quartet), and m (multiplet). High-resolution mass spectroscopy was measured by using Orbitrap Thermo Scientific Velos pro with HESI/heated electrospray ionization. Low-resolution mass spectroscopy was measured on an Advion Expression CMS Compact Mass Spectrometer using Atmospheric Solids Analysis Probe (ASAP®) or a TLC plate reader. Enantiomeric purities were determined by Chiralcel and Chiralpak (Daicel Chemical Industries Ltd.) columns on HPLC Agilent Technologies 1200 Infinity series using Chemstation software for LC systems. IR spectra were recorded on an Agilent Technologies Cary 630 FTIR spectrometer. Melting points were measured on an M-565 Büchi device.

2. Additional experimental results

Table S1. Reactions of **9** with 2-aminobenzenethiol (**8a**) and 2-aminophenyl disulfide (**S1**).

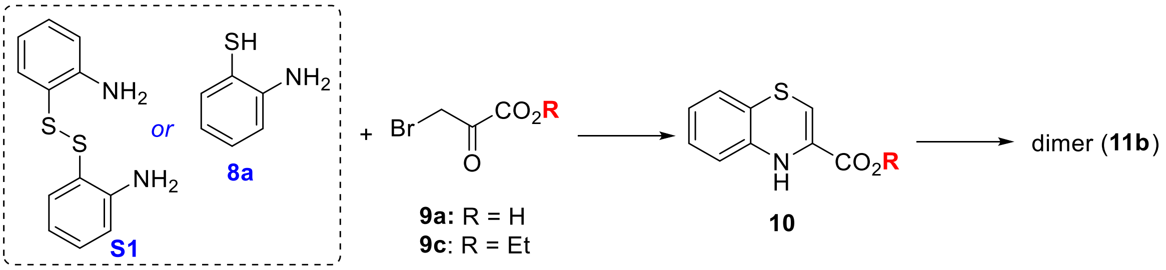

entry	8a/S1	R	conditions	yield
1	**S1**	Et	EtOH, rt, 24 h	—
2	**S1**	Et	EtOH, rt, 168 h	—
3	**S1**	Et	EtOH, reflux, 30 min	—
4	**8a**	Et	EtOH, MWI, 100 °C, 5 min	—
5	**8a**	Et	EtOH, rt, 24 h	—
6	**8a**	Et	EtOH, MWI, 100 °C, 20 min	(dimer, 28%)
7	**8a**	Et	EtOH, MWI, 100 °C, 1 h	(dimer, 11%)
8	**8a**	Et	EtOH, MWI, 60 °C, 1 h	(dimer, 20%)
9	**S1**	Et	EtOH, MWI, 100 °C, 20 min	—
10	**8a**	Et	EtOH, MWI, 100 °C, 20 min	—
11	**S1**	Et	EtOH, reflux, 10 min, $NaBH_4$	—
12	**8a**	Et	CH_2Cl_2, rt, 2 h	(dimer, 15%)
13	**S1**	Et	CH_2Cl_2, rt→reflux, 2 h	—
14	**8a**	Et	EtOH, rt, 40 min	51%
15	**8a**	Et	EtOAc, rt, 1 h	—
16	**S1**	Et	EtOAc, rt, 1 h	—
17	**8a**	Et	EtOH, 0 °C→rt, 2h	29%
18	**S1**	H	EtOH, reflux, 30 min	—
19	**8a**	H	EtOH, reflux, 30 min	—
20	**8a**	H	EtOH, MWI, 60 °C, 1 h	—
21	**8a**	H	CH_2Cl_2, MWI, 100 °C, 1 h	—
22	**8a**	H	Et_2O, reflux, 10 min	—
23	**8a**	H	Et_2O, rt, 10 min	—
24	**8a**	H	Et_2O, 0 °C→rt, 10 min	—
25	**8a**	H	Et_2O, 0 °C, 15 min	traces
26	**8a**	H	Et_2O, 0 °C, 1 h	traces
27	**8a**	H	EtOH, 0 °C, 30 min	—
28	**8a**	H	THF, 0 °C, 30 min	—
29	**S1**	H	DMF, rt, 24h	—
30	**8a**	H	DMF, rt, 24h	—
31	**8a**	H	EtOAc, 0 °C, 1 h	—
32	**8a**	H	Et_2O, rt, 1 h	—
33	**8a**	H	Et_2O, 0 °C, 1 h	75%

Table S2. Reactions of **9** with 2-amino-4-methoxybenzenethiol (**8c**) and 2-amino-4-methoxyphenyl disulfide (**S2**).

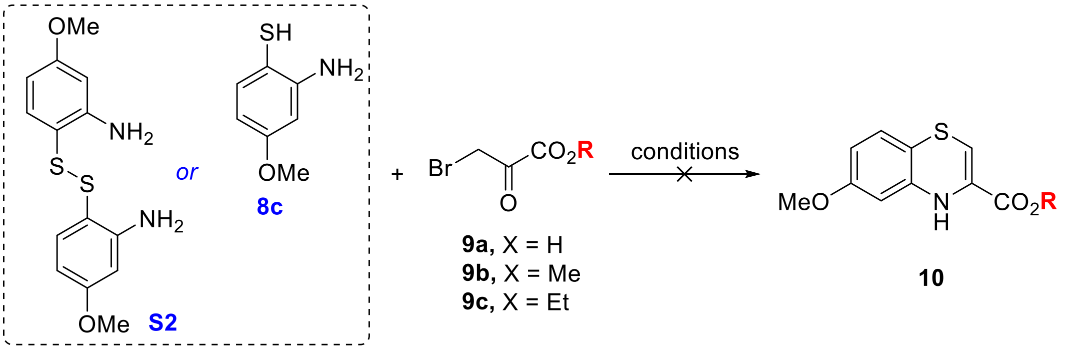

entry	8c/S2	R	conditions	yield
1	**S2**	Et	EtOH, MWI, 100 °C, 20 min	—
2	**S2**	Et	EtOH, reflux, 10 min	—
3	**S2**	Et	EtOH, rt, 10 min→24 h	—
4	**S2**	Et	EtOH, rt, 10 min, TsOH	—
5	**S2**	Et	EtOH, reflux, 10 min, $NaBH_4$	—
6	**S2**	Et	EtOH, MWI, 100 °C, 20 min (2 equiv of ester)	—
7	**S2**	Et	EtOH, rt, 24 h	—
8	**S2**	Et	Et_2O, rt, 24 h	—
9	**S2**	Et	MeOH, rt, 24 h	—
10	**S2**	Et	CH_2Cl_2, rt, 24 h	—
11	**S2**	Et	CH_2Cl_2, rt, 1 h	—
12	**S2**	Et	EtOH, MWI, 100 °C, 5 min	—
13	**S2**	Et	CH_2Cl_2, rt, 2 h (2 equiv of disulfide)	—
14	**S2**	Et	CH_2Cl_2, rt, 2 h	—
15	**S2**	Et	CH_2Cl_2, rt, 2 h (2 equiv of ester)	—
16	**S2**	Et	CH_2Cl_2, rt, 2 h (3 equiv of ester)	—
17	**S2**	Et	EtOH, rt, 1 h (2.5 equiv of ester)	—
18	**S2**	Et	CH_2Cl_2, rt, 1 h (2.5 equiv of ester)	—
19	**S2**	Et	Et_2O, rt, 1 h (2.5 equiv of ester)	—
20	**8c**	Et	EtOH, rt, 40 min	—
21	**8c**	Et	EtOH, 0 °C – rt, 1 h	—
22	**S2**	H	Et_2O, rt, 20 min	—
23	**S2**	H	Et_2O, rt, 24 h	—
24	**S2**	H	Et_2O, rt, 20 h (acid–base extraction)	—
25	**S2**	H	Et_2O, rt, 1 h	—
26	**S2**	H	Et_2O, rt, 1 h (acid–base extraction)	—
27	**8c**	H	Et_2O, 0 °C, 1 h	—
28	**S2**	H	EtOH, rt, 2 h	—
29	**S2**	H	EtOH, reflux, 10 min	—
30	**8c**	H	Et_2O, 0 °C, 1 h	—
31	**8c**	H	Et_2O, rt, 24 h	—
32	**8c**	H	EtOH, rt, 24 h	—
33	**8c**	H	EtOH, reflux, 1 h	—
34	**8c**	Me	EtOH, rt, 3 h	—
35	**S2**	Me	EtOH, rt, 3 h	—

Table S3. Reactions of **9** with 2-amino-4-chlorobenzenethiol (**8b**).

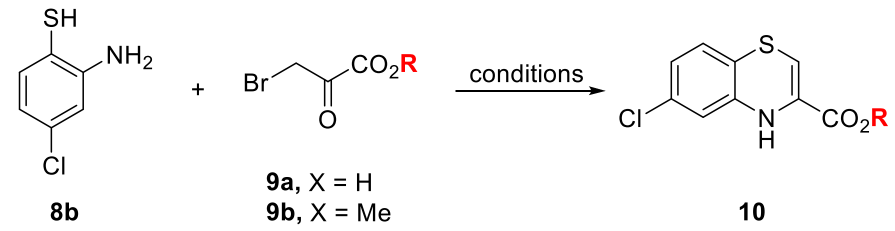

entry	R	conditions	yield (%)
1	Me	EtOH, rt, 30 min	50
2	Me	Et_2O, rt, 1 h	30
3	Me	EtOH, rt, 1 h	44
4	H	EtOH, rt, 30 min	43
5	H	EtOH, rt, 60 min	66

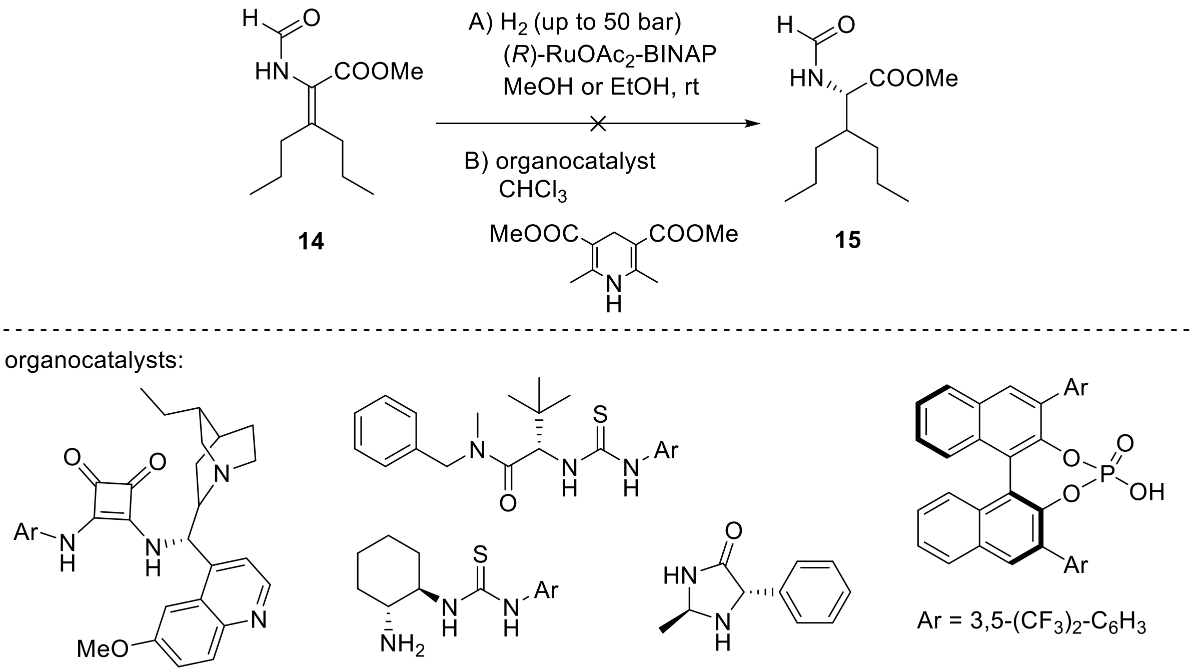

Scheme S1. Unsuccessful stereoselective syntheses of formyl-protected amino acid methyl ester **15**.

Table S4. Reaction of benzothiazine acid **10aa** with L-valine (**S3**).

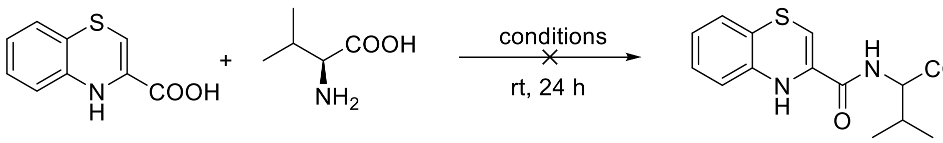

entry	**10aa** (equiv)	**S3** (equiv)	reagents (equiv)	solvent	yield (%)
1	1.0	1.0	EDC (1.0), DIPEA (1.0)	CH_2Cl_2	—
2	1.0	1.0	EDC (1.0), DIPEA (1.0)	CH_3CN	—
3	1.0	1.0	EDC (1.0), DIPEA (1.5)	CH_2Cl_2	—
4	1.5	1.0	$T_3P^{®}$ (1.2), DMAP (0.1), DIPEA (2.0)	CH_2Cl_2	—
5	1.7	1.1	HOBt (2.0), DIPEA (1,1)	CH_2Cl_2	—
6	1.0	1.1	HOBt (2.0), DIPEA (1,1)	CH_2Cl_2	—
7	1.0	1.1	HOBt (2.0), EDC (1.0), DIPEA (1.1)	CH_2Cl_2	—

Table S5. Reaction of benzothiazine acid **10aa** with methyl L-valinate **S3**·HCl.

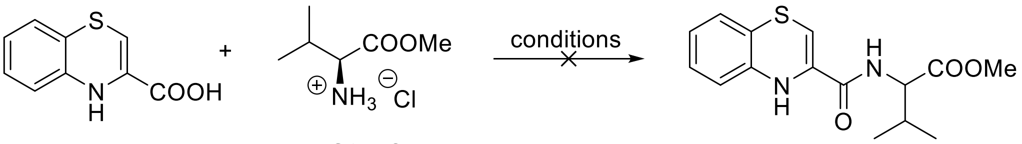

entry	**10aa** (equiv)	**S3**·HCl (equiv)	reagents (equiv)	solvent	*T* (°C)	*t* (h)	yield (%)
1	1.0	1.1	HOBt (1.0), DIPEA (1.1)	CH_2Cl_2	rt	24	—
2	1.2	1.0	EDC (1.0), HOBt (0.1), DMAP (1.0), DIPEA (1.0)	CH_3CN	rt	24	—
3	1.0	1.0	EDC (1.4), DCC (1.2), DIPEA (1.0)	BM (25 Hz), EtOAc (LAG)		10 min	—
4	1.0	1.0	EDC (1.4), DCC (1.2), DIPEA (1.0)	BM (25 Hz), EtOAc (LAG)		1	—
5	1.0	1.0	EDC (1.4), DCC (1.2), DIPEA (1.0)	EtOAc	0	24	—

Table S6. Unsuccessful connection of the amino acid methyl ester **16a**·HCl with the benzothiazine **10**.

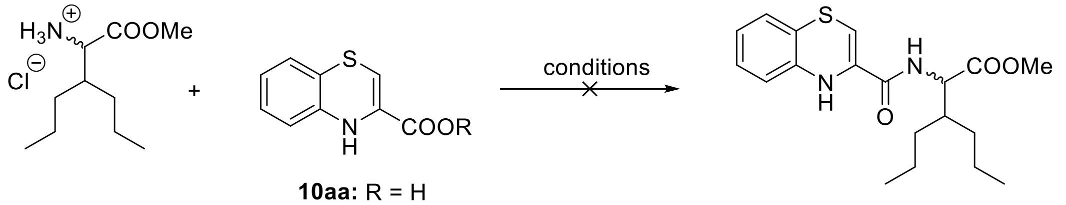

16·HCl **10aa:** R = H **10ac:** R = Et

entry	**16a**·HCl (equiv)	R	**10** (equiv)	reagents (equiv)	solvent	*T* (°C)	*t* (h)	yield (%)
1	1.2	Et	1.0	KO*t*-Bu (1.85)	BM (30 Hz), no solvent		2.5	—
2	1.0	H	1.0	EDC (1.0), DIPEA (1.0)	CH_2Cl_2	rt	o/n	—
3	1.0	H	1.0	EDC (1.2), HOBt (1.2), DIPEA (3.5)	DMF	0 °C→ rt	o/n	—
4	1.0	H	1.0	$SOCl_2$ (1.0), TEA (4.0)	CH_2Cl_2	rt	o/n	—
5	1.2	H	1.0	$SOCl_2$ (138.0), DMF (cat), DIPEA (3.0)	THF	rt	o/n	—
6	1.0	H	1.5	$(COCl)_2$, DMF (cat), TEA	CH_2Cl_2, DMF	rt	o/n	—
7	1.0	H	1.0	COMU (1.0), TEA (3.0)	DMF	rt	o/n	traces

Table S7. Reaction of benzothiazine ester **10bb** with racemic amino acid methyl ester **16a**.

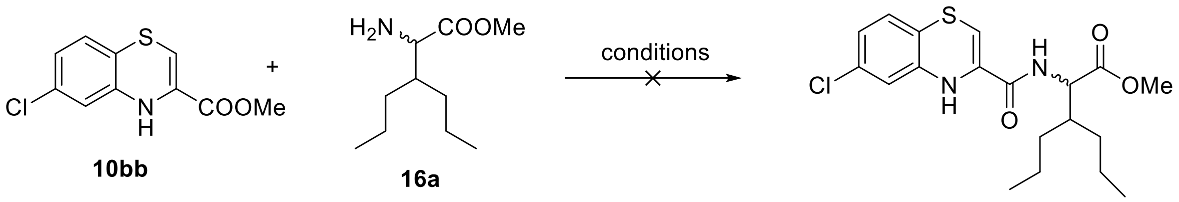

10bb **16a**

entry	**10bb** (equiv)	**16a** (equiv)	reagents (equiv)	solvent	*T* (°C)	*t* (h)	yield (%)
1	1.2	1.0	*t*-BuOK (0.85)	BM (30 Hz), no solvent		10	—
2	1.2	1.0	*t*-BuOK (0.85)	BM (30 Hz), EtOAc (LAG)		60	—
3	1.0	1.0	EDC (1.0), DIPEA (1.0)	CH_2Cl_2	rt	24	—
4	1.0	1.0	EDC (1.0), DIPEA (2.0)	CH_2Cl_2, DMF	rt	48	—
5	1.0	1.0	EDC (1.2), HOBt (1.2), DIPEA (1.0)	DMF	0 °C→ rt	24	—
6	1.0	1.0	EDC (1.0), HOBt (1.0), DIPEA (1.0)	CH_3CN, DMF	0 °C→rt	24	—

7	1.0	1.0	EDC (1.0), HOBt (1.0), DIPEA (1.0)	DMF	0 °C	15	—
8	1.0	1.0	TBTU (1.0), DIPEA (1.0)	DMF	0 °C	15	—

Table S8. Reaction of benzothiazine acid **10ba** with methyl L-valinate **S3**·HCl.

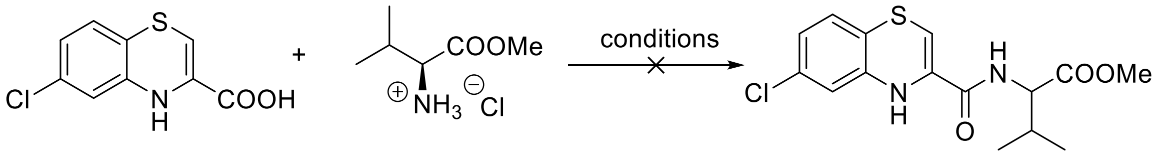

10ba **S3·HCl**

entry	**10ba** (equiv)	**S3**·HCl (equiv)	reagents (equiv)	solvent	*T* (°C)	*t* (h)	yield (%)
1	1.0	1.0	EDC (2.0), HOBt (2.0), DIPEA (2.0)	DMF	0 °C→ rt	24	—

Table S9. Unsuccessful reaction of bromopyruvic acid (**9a**) and **16a**·HCl.

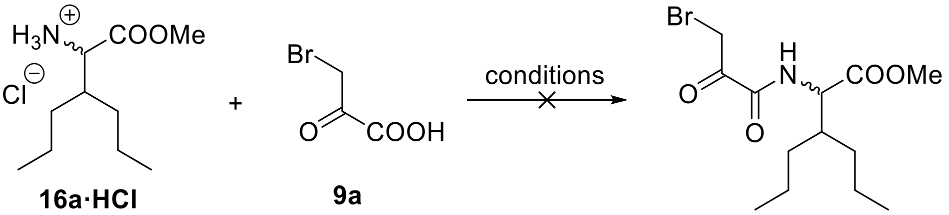

16a·HCl **9a**

entry	**16a**·HCl (equiv)	**9a** (equiv)	reagents (equiv)	solvent	*T* (°C)	*t* (h)	yield (%)
1	1.0	1.0	EDC (1.2), HOBt (1.2), DIPEA (3.5)	CH_2Cl_2	0 C°→ rt	o/n	—
2	1.0	1.1	EDC (1.2), TEA (1.0)	CH_3CN	0 °C→ rt	o/n	—

Table S10. Reaction of amine **16a** with pyruvic acid.

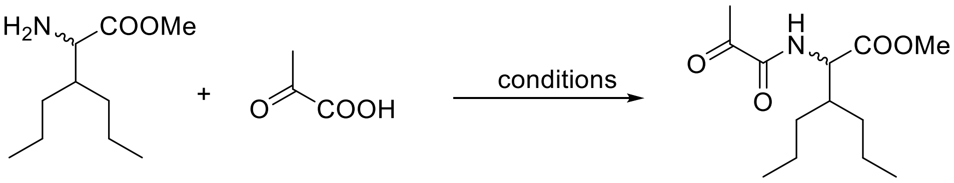

16a **17a**

entry	**16a** (equiv)	acid (equiv)	reagents (equiv)	solvent	*T* (°C)	*t* (h)	yield (%)
1	1.1	1.0	$T_3P^{®}$ (2.0), *N*-Me-morpholine (3.0)	CH_3CN	rt	o/n	34
2	1.0	1.2	DCC (2.2), DMAP (1.0)	CH_2Cl_2	0 °C→rt	24	18

3	1.0	1.0	$SOCl_2$ (1.1), TEA (3.0)	THF	reflux, then rt	o/n	19
4	1.1	1.0	$(COCl)_2$ (1.2), DMF (cat), TEA (3.0)	CH_2Cl_2	rt	o/n	10

3. Experimental procedures

General procedure for the synthesis of benzothiazine esters

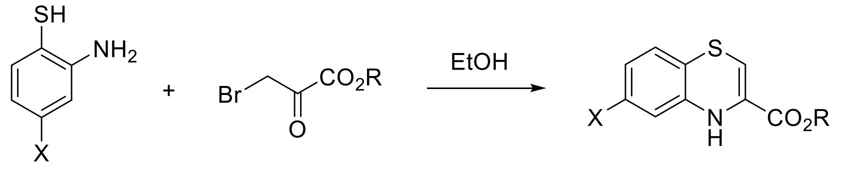

8a: X = H
8b: X = Cl

9b: R = Me
9c: R = Et

10ac: X = H, R = Et
10bb: X = Cl, R = Me

Aminothiol **8a** or **8b** (16.0 mmol) was dissolved in absolute EtOH (25 mL, 99.8% grade) at room temperature under nitrogen atmosphere and was cooled down with an ice bath. Next, ester **9b** or **9c** (19.0 mmol) was added dropwise, and the reaction mixture was stirred at the same temperature for 0.5–2 h. The conversion of the starting material was monitored by TLC analysis(hexane/EtOAc 5:1 or 7:1, anisaldehyde visualization). The reaction was quenched by the addition of cold distilled water (25 mL). Then, CH_2Cl_2 (30 mL) was added. The separated organic phase was washed with 1 M NaOH aqueous solution (1 × 25 mL), saturated NaCl solution (1 × 25 mL), and dried over $MgSO_4$. The solvent was evaporated under reduced pressure and the crude reaction mixture was purified by column chromatography (SiO_2, hexane/EtOAc 7:1 to 5:1). If necessary, the product was crystallized from MeOH in the freezer overnight. The products were obtained as orange or red solids.

Notes: The unsubstituted ethyl ester **10ac** is unstable, and the purification is problematic. Products decompose also when the reaction mixture is stirred for a longer time (e.g., overnight) or if a solution stands in an NMR tube for a few hours. Unexpected side products **11a** and **11b** were obtained when the reaction was proceeded under microwave irradiation or reflux.

General procedure for the synthesis of benzothiazine carboxylic acids

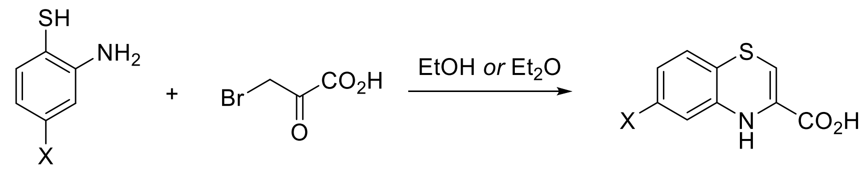

8a: X = H
8b: X = Cl

9a

10aa: X = H
10ba: X = Cl

Aminothiol **8a** or **8b** (4.0 mmol) was dissolved in absolute EtOH or dry Et_2O (15 mL) at room temperature under nitrogen atmosphere and was cooled down with an ice bath. Then, a solution of carboxylic acid **9a** (4.2 mmol) in EtOH or Et_2O (5 mL) was added dropwise, and the reaction was stirred vigorously at the same temperature for 0.5–1 h. The mixture was warmed to room temperature and the conversion of the starting material was monitored by TLC (hexane/EtOAc 5:1, anisaldehyde visualization). The reaction was quenched with saturated

$NaHCO_3$ solution (25 mL) and was extracted with CH_2Cl_2 (3 × 20 mL). The aqueous phase was transferred to a beaker and was stirred vigorously. Then, it was acidified dropwise (Pasteur pipette) with conc HCl to roughly pH 1 (as controlled with litmus paper). The resulting solid was filtered off, washed with distilled water and hexane and then dried under high vacuum. Products were obtained as red or orange solids.

Methyl 2-formamido-3-propylhex-2-enoate (14)

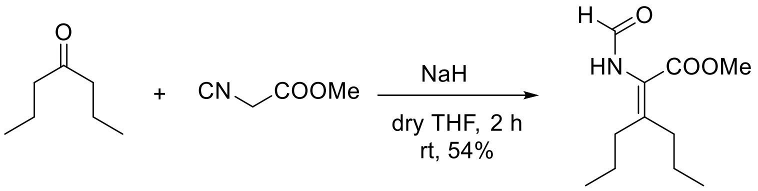

12 **13** **14**

A solution of methyl cyanoacetate (**13**, 910 µL, 10.0 mmol) and heptan-4-one (**12**, 1.4 mL, 10.0 mmol) in dry THF (10 mL) was added dropwise to a mixture of NaH (60% dispersion in mineral oil, 0.48 g, 12.0 mmol) in dry THF (10 mL). The reaction mixture was stirred at ambient temperature for 2 h. The progress of the reaction was monitored by TLC analysis (hexane/EtOAc 2:1, $KMnO_4$ visualization). Next, the mixture was cooled down with an ice bath, and water was added slowly dropwise until the remaining NaH was consumed. Then, the volatile components were removed by evaporation under vacuum. The remaining aqueous aliquot was extracted with CH_2Cl_2 (3 × 50 mL). The combined organic phase was dried over anhydrous Na_2SO_4, and the solvent was evaporated under reduced pressure. The crude product was purified by flash chromatography (SiO_2, hexane/EtOAc 1.5:1) to yield the product **14** as a colorless liquid (1.15 g, 54%). While standing in the fridge, the product slowly solidified.

Methyl 2-formamido-3-propylhexanoate (15)

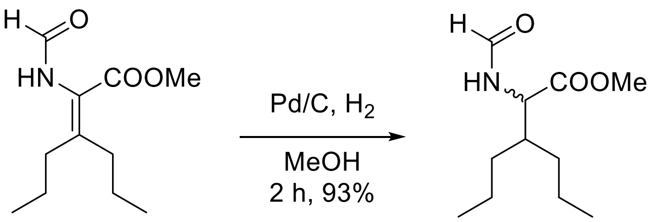

14 **15**

Method A: The starting material methyl 2-formamido-3-propylhex-2-enoate (**14**, 1.15 g, 5.4 mmol) was dissolved in MeOH (50 mL). Next, 10% Pd/C (280 mg, 25 wt %) was added, and the mixture was hydrogenated (using a balloon) for 2 h at room temperature. The reaction mixture was filtered through celite, and the solvent was evaporated under reduced pressure to gain the title compound **15** as a colorless oil (1.07 g, 93%). The product was used without further purification. While standing in the fridge, the product slowly solidified. The rotation of the amide bond was partially restricted. Based on the ^{1}H NMR, the isomeric ratio was 9:1.

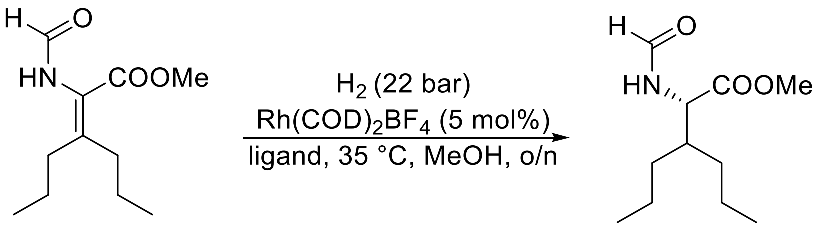

14 (*S*)-**15**

Method B (general procedure for stereoselective hydrogenation): The starting material methyl 2-formamido-3-propylhex-2-enoate (**14**, 50 mg, 0.23 mmol), ligand (0.06 equiv), and $Rh(COD)_2BF_4$ (5 or 6 mol %) were dissolved in degassed MeOH (2 mL, Ar). The mixture was hydrogenated (22 bar) for 24 h at 35 °C. The reaction mixture was filtered through celite, and the solvent was evaporated under reduced pressure. The crude product was purified by flash chromatography (SiO_2, hexane/EtOAc 2:1) to yield the product **15** as a colorless oil.

Methyl 2-amino-3-propylhexanoate (16a)

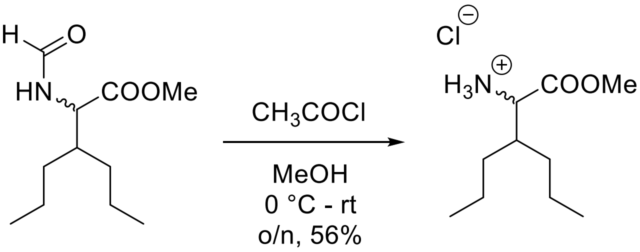

15 **16a·HCl**

Method A: First, the starting material methyl 2-formamido-3-propylhexanoate (**15**, 1.068 g, 4.9 mmol) was dissolved in MeOH (20 mL), and the solution was cooled with an ice bath. Next, acetyl chloride (1.3 mL, 18.3 mmol) was added dropwise to the reaction mixture. After the addition, the ice bath was removed, and the reaction mixture was stirred at ambient temperature overnight. Next, a few drops of water were added to discharge the remaining acetyl chloride, and the volatile components were removed under reduced pressure. The remaining material was dissolved in CH_2Cl_2 (50 mL) and washed with water (3 × 50 mL). The organic phase was dried over anhydrous Na_2SO_4, and the solvent was evaporated under reduced pressure. The crude product was purified by flash chromatography (SiO_2, CH_2Cl_2 with up to 5% MeOH) to yield **16a**·HCl as an off-white solid (620 mg, 56%).

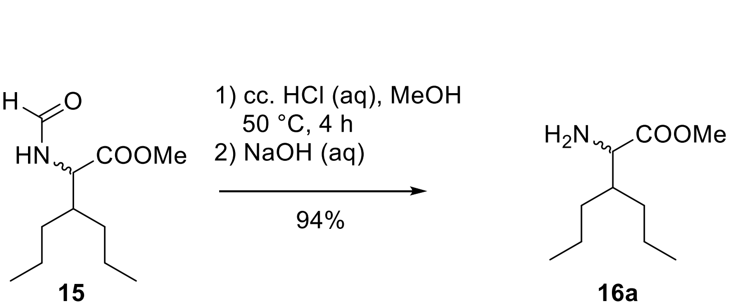

15 **16a**

Method B: First, the starting material methyl 2-formamido-3-propylhexanoate (**15**, 3 g, 13.9 mmol) was dissolved in MeOH (70 mL). Next, conc HCl (7 mL) was added dropwise. The reaction mixture was stirred at 50 °C for 4 h. After the reaction, 10% NaOH (aq) was added

(roughly pH 12), and the mixture was extracted with CH_2Cl_2 (3 × 50 mL). The combined organic phase was dried over anhydrous Na_2SO_4, and the solvent was evaporated under reduced pressure to gain the title compound **16a** as a colorless oil (2.466 g, 94%). The product was used without further purification.

Methyl 2-(2-oxopropanamido)-3-propylhexanoate (17a)

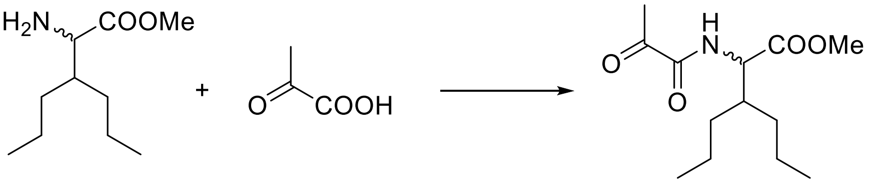

16a **17a**

Method A: First, methyl 2-amino-3-propylhexanoate (**16a**, 100 mg, 0.5 mmol) and *N*-methylmorpholine (160 µL, 1.0 mmol) were dissolved in dry MeCN (2 mL). Pyruvic acid (34 µL, 0.5 mmol) and $T_3P^{®}$ (50 wt % in EtOAc, 577 µL, 1.0 mmol) were then added to this mixture, and the reaction mixture was stirred at room temperature for 24 h. Next, the mixture was diluted with EtOAc (50 mL) and washed first with saturated $NaHCO_3$ (aq, 20 mL) and then with water (2 × 50 mL). The organic phase was dried over anhydrous Na_2SO_4, and the solvent was evaporated under reduced pressure. The crude product was purified by flash chromatography (SiO_2, hexane with EtOAc up to 10%) to yield the title compound (**17a**) as a colorless oil (42.4 mg, 34%).

Method B: Pyruvic acid (705 µL, 10.0 mmol) was dissolved in dry CH_2Cl_2 (5 mL). Then, $SOCl_2$ (798 µL, 11.0 mmol) was added, and the mixture was refluxed for 2 h. Then, the volatile components were removed under reduced pressure to gain pyruvoyl chloride, which was used without further purification in the next step. Next, methyl 2-amino-3-propylhexanoate (**16a**, 100 mg, 0.5 mmol) and triethyl amine (223 µL, 1.6 mmol) were dissolved in dry THF (1 mL). A solution of the previously prepared pyruvoyl chloride (110 mg, 1.1 mmol) in dry THF (1 mL) was added, and the mixture was stirred at room temperature for 24 h. Next, the volatile components were evaporated under reduced pressure, and the remaining mixture was diluted with water (50 mL) and extracted with EtOAc (3 × 50 mL). The combined organic phase was dried over anhydrous Na_2SO_4, and the solvent was evaporated under reduced pressure. The crude product was purified by flash chromatography (SiO_2, hexane with up to 10% EtOAc) to yield the title compound **17a** as a colorless oil (24.3 mg, 19%).

Method C: Pyruvic acid (270 µL, 3.8 mmol) was dissolved in dry CH_2Cl_2 (20 mL), and the mixture was cooled down with an ice bath. Next, DCC (792 mg, 3.8 mmol), DMAP (78 mg, 0.6 mmol), and a solution of methyl 2-amino-3-propylhexanoate (**16a**, 600 mg, 3.2 mmol) in dry CH_2Cl_2 (10 mL) were added, and the reaction mixture was stirred at ambient temperature. The next day, an additional amount of DCC (660 mg, 3.2 mmol) and DMAP (312 mg, 2.6 mmol) were added, and the stirring was continued for 2 days. The mixture was diluted with water (50 mL) and extracted with CH_2Cl_2 (3 × 50 mL). The combined organic phase was washed with saturated $NaHCO_3$ (aq, 30 mL), dried over anhydrous Na_2SO_4, and the solvent was evaporated under reduced pressure. The crude product was purified by flash chromatography (SiO_2,

hexane with EtOAc up to 10%) to yield the title compound **17a** as a colorless oil (146 mg, 18%).

Method D: Pyruvic acid (342 µL, 4.9 mmol) and a few drops of DMF were dissolved in dry CH_2Cl_2 (20 mL). Next, oxalyl chloride (505 µL, 5.9 mmol) was added, and the mixture was stirred for 2 h. The volatile components were removed under reduced pressure. The remaining oil was taken up in dry CH_2Cl_2 (20 mL) and was dropwise added to a solution of methyl 2-amino-3-propylhexanoate (**16a**, 1 g, 5.3 mmol) and triethyl amine (2 mL, 14.6 mmol) in CH_2Cl_2 (20 mL). The reaction mixture was stirred at room temperature overnight. The mixture was diluted with water (50 mL) and extracted with CH_2Cl_2 (3 × 50 mL). The combined organic phase was dried over anhydrous Na_2SO_4, and the solvent was evaporated under reduced pressure. The crude product was purified by flash chromatography (SiO_2, hexane with up to 10% EtOAc) to yield the title compound **17a** as a colorless oil (120 mg, 10%).

General procedure for the peptide couplings (17a and 17b):

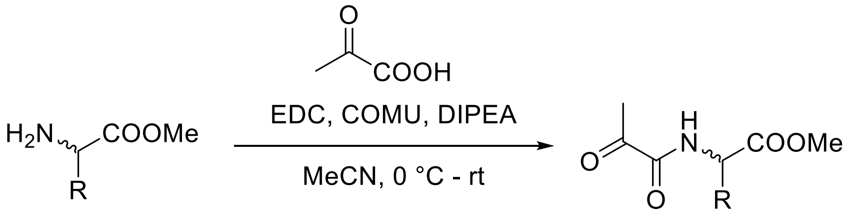

16a: R = $CHPr_2$ (rac)
16b: R = CH_2Ph (*S*)

17a: R = $CHPr_2$ (rac)
17b: R = CH_2Ph (*S*)

The amino acid methyl ester **16a** or **16b** (2.0 mmol), pyruvic acid (138 µL, 2.0 mmol), and COMU (1.3 g, 2.4 mmol) were dissolved in dry CH_3CN (20 mL), and the mixture was cooled with an ice bath to 0 °C. Next, a mixture of EDC·HCl (460 mg, 2.4 mmol) and DIPEA (1.22 mL, 7.0 mmol) in CH_3CN (20 mL) was slowly added to the reaction mixture. After 1 h, the ice bath was removed, and the mixture was stirred at ambient temperature for 2 days. After that, water (50 mL) and EtOAc (50 mL) were added, and the phases were separated. The organic phase was washed with water, dried over anhydrous Na_2SO_4, and concentrated under reduced pressure. The crude product was purified by flash chromatography (SiO_2, hexane/EtOAc 6:1 to 3:1) to yield the product as a colorless oil.

General procedure for the α-bromination reactions

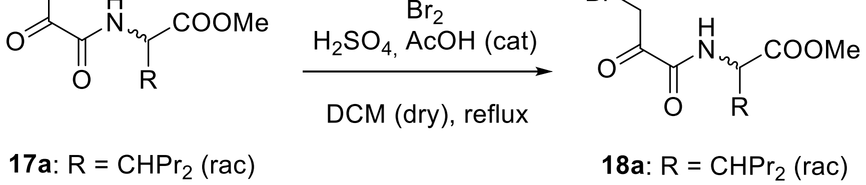

17a: R = $CHPr_2$ (rac)
17b: R = CH_2Ph (*S*)

18a: R = $CHPr_2$ (rac)
18b: R = CH_2Ph (*S*)

Starting material **17a** or **17b** (0.6 mmol) was dissolved in dry CH_2Cl_2 (20 mL). Then, bromine (60 µL, 1.1 mmol), three drops of conc H_2SO_4, and three drops of acetic acid were added. The reaction mixture was refluxed for 8 h. Then, 5 wt % $Na_2S_2O_3$ (aq, 10 mL) was added, and this

was stirred for 10 min, followed by extraction with CH_2Cl_2 (3 × 50 mL). The combined organic phase was washed with saturated $NaHCO_3$ (50 mL), water (50 mL), and dried over anhydrous Na_2SO_4. The solvent was evaporated under reduced pressure. The crude product was purified by flash chromatography (SiO_2, hexane:EtOAc 10:1) to yield the title compound **18a** or **18b** as a yellow oil.

General procedure for the cyclization reactions

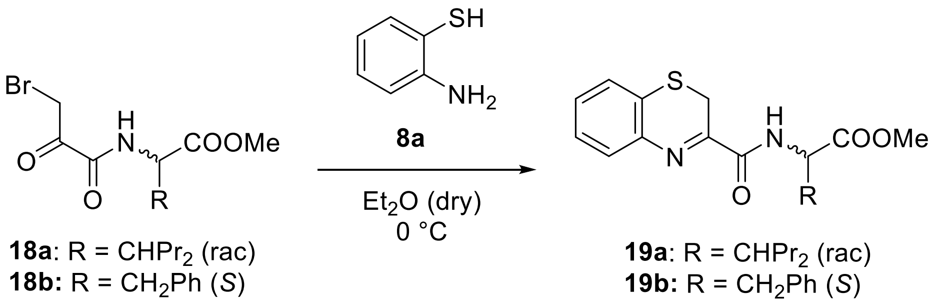

18a: R = $CHPr_2$ (rac)
18b: R = CH_2Ph (*S*)

19a: R = $CHPr_2$ (rac)
19b: R = CH_2Ph (*S*)

Bromine derivative **18a** or **18b** (0.4 mmol) was dissolved in freshly distilled dry Et_2O (20 mL) in an Ar atmosphere and cooled down with an ice bath. Then, 2-aminothiophenol (**8a**, 48 µL, 0.4 mmol) was added, and this was stirred at the same temperature for 2 h. The reaction mixture was concentrated under reduced pressure, and the crude product was purified by flash chromatography (SiO_2, hexane/EtOAc 10:1) to yield the title compound **19a or 19b** as a yellow oil.

4. Characterization data

4*H*-Benzo[*b*][1,4]thiazine-3-carboxylic acid (10aa)

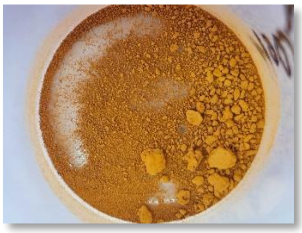
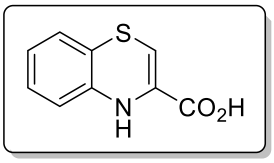

Orange solid, **Mp** 140 – 143 °C. **^{1}H NMR** (600 MHz, DMSO): δ 13.08 (br s, 1H), 7.27 (d, *J* = 1.9 Hz, 1H), 6.84 (m, 1H), 6.73 (d, *J* = 7.5 Hz, 1H), 6.65 (m, 2H), 5.72 (d, *J* = 1.8 Hz, 1H) ppm. **^{13}C NMR** (150 MHz, DMSO) δ 162.0, 142.5, 132.0, 128.5, 126.5, 123.4, 115.7, 114.7, 102.9 ppm. **2D NMR**: HSQC, COSY, HMBC. **IR** (ATR): 3340, 2816, 2718, 2626, 2528, 2475, 1654, 1617, 1576, 1437, 734 cm^{-1}. **MS** (APCI): *m/z* $[M+H]^+$ calcd. for $C_9H_8NO_2S$: 194.0, found: 194.0; $[M-CO_2H]^+$ calcd. for C_8H_6NS: 148.0, found: 148.0. **HRMS** (HESI): *m/z* $[M+H]^+$ calcd for $C_9H_8NO_2S$: 194.0270; found: 194.0271; $[M]^+$ calcd for $C_9H_7NO_2S$: 193.0192, found: 193.0193.

Ethyl 4*H*-benzo[*b*][1,4]thiazine-3-carboxylate (10ac)

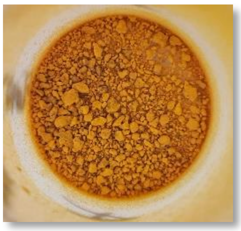
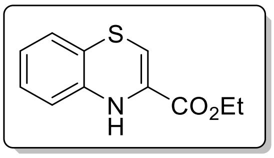

Orange solid, **Mp** 77 – 78 °C. **^{1}H NMR** (600 MHz, $CDCl_3$): δ 6.91 (m, 1H), 6.80 – 6.66 (m, 2H), 6.37 (d, *J* = 7.9 Hz, 1H), 5.86 (s, 2H), 4.24 (q, *J* = 7.1 Hz, 2H), 1.30 (t, *J* = 7.1 Hz, 3H) ppm. **^{13}C NMR** (300 MHz, $CDCl_3$) δ 160.7, 141.5, 130.7, 128.2, 126.6, 123.6, 115.2, 114.8, 105.2, 61.8, 14.2 ppm. **IR** (ATR): 3366, 2974, 1692, 1578, 1462, 1440, 1370, 1321, 1286, 1226, 1090, 761, 721 cm^{-1}. **MS** (APCI): *m/z* $[M+H]^+$ calcd. for $C_{11}H_{12}NO_2S$: 222.1, found: 222.0; $[M-CO_2Et]^+$ calcd. for C_8H_6NS: 148.0, found: 148.0. **HRMS** (HESI): *m/z* $[M+H]^+$ calcd for $C_{11}H_{12}NO_2S$: 222.0583; found: 222.0539; $[M]^+$ calcd for $C_{11}H_{11}NO_2S$: 221.0505, found: 221.0505.

Diethyl 4*H*,4'*H*-[2,2'-bibenzo[*b*][1,4]thiazine]-3,3'-dicarboxylate (11a)

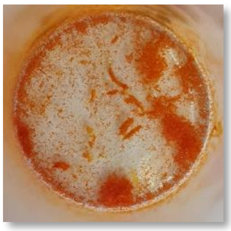
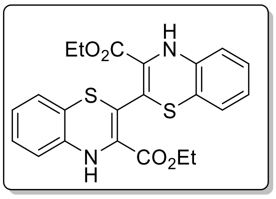

Pale red solid, **Mp** 188 – 192 °C. **^{1}H NMR** (600 MHz, $CDCl_3$): δ 6.98 (td, *J* = 7.6, 1.5 Hz, 1H), 6.91 (dd, *J* = 7.7, 1.5 Hz, 1H), 6.84 (td, *J* = 7.5, 1.2 Hz, 1H), 6.55 (dd, *J* = 7.8, 1.3 Hz, 1H), 6.44 (br s, 1H), 4.23 – 4.10 (m, 2H), 1.15 (t, *J* = 7.2 Hz, 3H) ppm. **^{13}C NMR** (150 MHz, $CDCl_3$): δ 161.3, 141.8, 129.6, 127.8, 126.5, 123.7, 118.1, 115.1, 114.6, 62.3, 13.8 ppm. **2D NMR:** HSQC, COSY, HMBC. **IR** (ATR): 3359, 1669, 1576, 1466, 1427, 1369, 1293, 1176, 1007, 746 cm^{-1}. **MS** (APCI): *m/z* $[M+H]^+$ calcd. for $C_{22}H_{21}N_2O_4S_2$: 441.1, found: 440.8; $[M-OEt]^+$ calcd. for

$C_{20}H_{15}N_2O_3S_2$: 395.1, found: 394.7. **HRMS** (HESI): *m/z* $[M+H]^+$ calcd. for $C_{22}H_{21}N_2O_4S_2$: 441.0937, found: 441.0942; $[M+Na]^+$ calcd. for $C_{22}H_{20}N_2O_4S_2Na$: 463.0757, found: 463.0756; $[M+K]^+$ calcd. for $C_{22}H_{20}N_2O_4S_2K$: 479.0496, found: 479.0498.

Diethyl 2*H*,2'*H*-[2,2'-bibenzo[*b*][1,4]thiazine]-3,3'-dicarboxylate (11b)

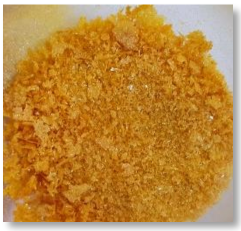

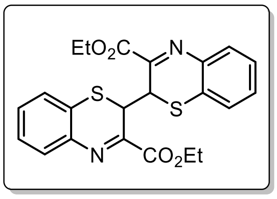

Yellow solid, **Mp** 205 – 209 °C. **^{1}H NMR** (600 MHz, $CDCl_3$): δ 7.75 (dd, *J* = 7.4, 1.8 Hz, 2H), 7.43 – 7.26 (m, 6H), 4.44 (dq, *J* = 10.8, 7.1 Hz, 2H), 4.32 – 4.19 (m, 4H), 1.33 (t, *J* = 7.1 Hz, 6H) ppm. **^{13}C NMR** (150 MHz, $CDCl_3$): δ 162.4, 146.5, 139.9, 129.0, 128.7, 127.0, 126.2, 120.4, 61.8, 29.1, 13.1 ppm. **IR** (ATR): 2979, 1709, 1441, 1330, 1286, 1224, 1084, 1063, 862, 762 cm^{-1}. **MS** (APCI): *m/z* $[M+H]^+$ calcd. for $C_{22}H_{21}N_2O_4S_2$: 441.1, found: 440.7; $[M-OEt]^+$ calcd. for $C_{20}H_{15}N_2O_3S_2$: 395.1, found: 394.6. **HRMS** (HESI): *m/z* $[M+H]^+$ calcd. for $C_{22}H_{21}N_2O_4S_2$: 441.0937, found: 441.0937; $[M+Na]^+$ calcd. for $C_{22}H_{20}N_2O_4S_2Na$: 463.0757, found: 463.0757; $[M+K]^+$ calcd. for $C_{22}H_{20}N_2O_4S_2K$: 479.0496, found: 479.0495.

6-chloro-4*H*-benzo[*b*][1,4]thiazine-3-carboxylic acid (10ba)

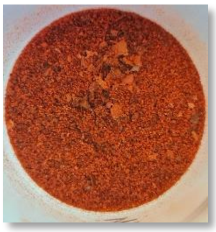

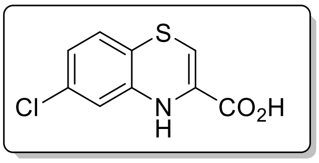

Red solid, **Mp** 192 – 195 °C. **^{1}H NMR** (600 MHz, DMSO): δ 7.51 (s, 1H), 6.81 (d, *J* = 2.1 Hz, 1H), 6.69 – 6.62 (m, 2H), 5.74 (d, *J* = 1.8 Hz, 1H) ppm. **^{13}C NMR** (150 MHz, DMSO): δ 161.7, 144.2, 132.9, 131.4, 127.6, 122.7, 115.0, 113.9, 103.5 ppm. **IR** (ATR): 3354, 1653, 1623, 1573, 1437, 1235, 1090, 880, 843, 796, 729 cm^{-1}. **MS** (APCI): *m/z* $[M+H]^+$ calcd. for $C_9H_7ClNO_2S$: 228.0, found: 227.8; $[M-CO_2H]^+$ calcd. for C_8H_5ClNS: 182.0, found: 181.8. **HRMS** (HESI): *m/z* $[M]^+$ calcd for $C_9H_6ClNO_2S$: 226.9802, found: 226.9803; (APPI): $[M+H]^+$ calcd for $C_9H_7ClNO_2S$: 227.9881; found: 227.9882.

Methyl 6-chloro-4*H*-benzo[*b*][1,4]thiazine-3-carboxylate (10bb)

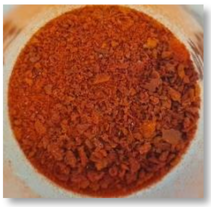
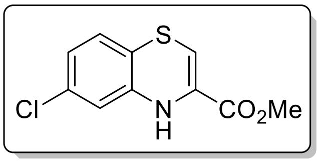

Red solid, **Mp** 144 – 146 °C. ^{1}**H NMR** (600 MHz, $CDCl_3$): δ 6.70 (dd, *J* = 8.0, 2.0 Hz 1H), 6.61 (d, *J* = 8.0 Hz, 1H), 6.37 (d, *J* = 2.0 Hz, 1H), 5.86 (br s, 2H), 3.79 (s, 3H) ppm. ^{13}**C NMR** (150 MHz, $CDCl_3$): δ 160.9, 142.7, 133.9, 129.8, 127.2, 123.3, 114.2, 113.8, 105.9, 52.7 ppm. **IR** (ATR): 3373, 1696, 1628, 1472, 1432, 1384, 1253, 1094, 860, 802, 771, 727 cm^{-1}. **MS** (APCI): *m/z* $[M+H]^+$ calcd. for $C_{10}H_9ClNO_2S$: 242.0, found: 241.9. **HRMS** (HESI): *m/z* $[M]^+$ calcd for $C_{10}H_8ClNO_2S$: 240.9959; found: 240.9961; $[M+Na]^+$ calcd for $C_{10}H_8ClNO_2SNa$: 263.9857, found: 263.9814.

Methyl 2-formamido-3-propylhex-2-enoate (14)

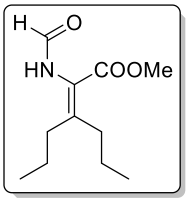

White solid, **Mp** 56 – 58 °C. ^{1}**H NMR** (600 MHz, $CDCl_3$): δ 8.20 (d, *J* = 1.4 Hz, 0.6H), 7.95 (d, *J* = 11.6 Hz, 0.4H), 6.67 (br s, 0.6H), 6.55 (d, *J* = 11.6 Hz, 0.4H), 3.76 (s, 1.2H), 3.75 (s, 1.8H), 2.54 (m, 0.8H), 2.47 (m, 1.2H), 2.25 (m, 0.8H), 2.15 (m, 1.2H), 1.56 – 1.43 (m, 4H), 1.00 – 0.92 (m, 6H) ppm. ^{13}**C NMR** (150 MHz, $CDCl_3$): δ 165.96, 164.94, 164.82, 159.81, 157.26, 153.30, 120.61, 120.04, 52.07, 51.91, 35.62, 35.41, 34.33, 34.12, 22.24, 22.15, 21.17, 20.74, 14.37, 14.35, 14.29 ppm. **IR** (ATR): 3266, 2957, 2932, 2870, 1723, 1685, 1657, 1506, 1463, 1435, 1385, 1316, 1274, 1251, 1203, 1134, 1095, 1034, 1000, 939, 873, 789, 746, 722, 677, 633, 548, 479 cm^{-1}. **MS** (MM-ES+APCI): *m/z* $[M+H]^+$ calcd. for $C_{11}H_{20}NO_3$: 214.1, found: 214.1; $[M-H]^+$ calcd. for $C_{11}H_{18}NO_3$: 212.1, found: 212.1. **HRMS** (HESI): *m/z* $[M+H]^+$ calcd. for $C_{11}H_{20}NO_3$: 214.14377, found: 214.14382; $[M+Na]^+$ calcd. for $C_{11}H_{19}NO_3Na$: 236.1257, found: 236.1255; $[M+K]^+$ calcd. for $C_{11}H_{19}NO_3K$: 252.0997, found: 252.0995.

Methyl 2-formamido-3-propylhexanoate (15)

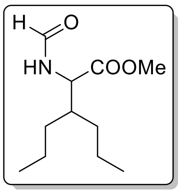

White solid, **Mp** 53 – 56 °C. The rotation of the amide bond is restricted. Based on the ^{1}H NMR, the isomeric ratio is 9:1. ^{1}**H NMR** (600 MHz, $CDCl_3$): δ 8.25 (d, *J* = 1.6 Hz, 0.9H), 7.99 (d, *J* = 11.8 Hz, 0.1H), 6.02 (d, *J* = 8.5 Hz, 0.9H), 5.92 (m, 0.1H), 4.85 (dd, *J* = 9.2, 3.7 Hz, 0.9H), 4.14 (dd, *J* = 10.2, 4.1 Hz, 0.1H), 3.77 (s, 0.35H), 3.75 (s, 2.65H), 1.91 (m, 1H), 1.50 – 1.14 (m, 8H), 0.96 – 0.85 (m, 6H) ppm. ^{13}**C NMR** (150 MHz, $CDCl_3$): δ 172.57, 171.92, 163.62, 160.78, 56.90,

52.57, 52.52, 52.33, 40.71, 40.42, 32.85, 32.41, 32.31, 31.67, 20.27, 20.25, 20.10, 20.03, 14.18, 14.15, 14.07 ppm. **IR** (ATR): 3234, 3047, 2958, 2931, 2872, 1743, 1644, 1544, 1458, 1439, 1377, 1345, 1322, 1284, 1242, 1204, 1130, 1116, 1049, 1003, 932, 787, 736, 669, 597, 454, 430 cm^{-1}. **MS** (APCI): *m/z* $[M+H]^+$ calcd. for $C_{11}H_{22}NO_3$: 216.2, found: 216.0; $[M-CO_2CH_3]^+$ calcd. for $C_9H_{18}NO$: 156.1, found: 155.9; $[M-H]^+$ calcd. for $C_{11}H_{20}NO_3$: 214.2, found: 213.8. **HRMS** (HESI): *m/z* $[M+H]^+$ calcd. for $C_{11}H_{22}NO_3$: 216.1594, found: 216.1595; $[M+Na]^+$ calcd. for $C_{11}H_{21}NO_3Na$: 238.1414, found: 238.1412; $[M+K]^+$ calcd. for $C_{11}H_{21}NO_3K$: 254.1153, found: 254.1152. **HPLC:** Chiralpak IA, hexane/i-PrOH 90:10, 1.0 mL min^{-1}, 215 nm, t_R(minor) = 5.536 min, t_R(major) = 7.360 min.

Methyl 2-amino-3-propylhexanoate·HCl (16a·HCl)

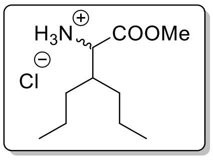

Off-white solid. **M.p.** 114.0 – 117.4 °C**.** **^{1}H NMR** (600 MHz, $CDCl_3$): δ 8.91 (s, 3H), 4.05 (s, 1H), 3.82 (s, 3H), 2.14 (m, 1H), 1.67 (m, 1H), 1.52 – 1.29 (m, 7H), 0.99 – 0.86 (m, 6H) ppm. **^{13}C NMR** (100 MHz, $CDCl_3$): δ 169.03, 55.40, 53.03, 39.09, 32.00, 31.95, 20.28, 20.22, 13.97, 13.92 ppm. **IR** (ATR): 2953, 2929, 2867, 2611, 1738, 1579, 1507, 1465, 1449, 1436, 1375, 1305, 1229, 1170, 1128, 1068, 1045, 1003, 967, 933, 901, 880, 814, 785, 740, 643, 544, 503, 438 cm^{-1}. **MS** (APCI): *m/z* $[M]^+$ calcd. for $C_{10}H_{22}NO_2$: 188.2, found: 188.0. **HRMS** (HESI): *m/z* $[M+H]^+$ calcd. for $C_{10}H_{22}NO_2$: 188.1645, found: 188.1643.

Methyl 2-amino-3-propylhexanoate (16a)

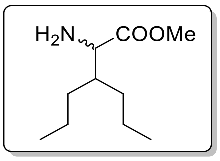

Transparent oil. **^{1}H NMR** (600 MHz, $CDCl_3$): δ 3.72 (s, 3H), 3.52 (d, *J* = 3.8 Hz, 1H), 1.78 (m, 1H), 1.48 – 1.16 (m, 8H), 0.92 (t, *J* = 6.8 Hz, 3H), 0.87 (t, *J* = 7.0 Hz, 3H) ppm. **^{13}C NMR** (100 MHz, $CDCl_3$): δ176.81, 56.32, 51.79, 41.23, 33.11, 31.72, 20.45, 20.41, 14.32, 14.26 ppm. **IR** (ATR): 3387, 2956, 2929, 2871, 1732, 1601, 1458, 1436, 1378, 1206, 1167, 1095, 1004, 922, 840, 743 cm^{-1}. **MS** (APCI): *m/z* $[M+H]^+$ calcd. for $C_{10}H_{22}NO_2$: 187.2, found: 188.0; $[M-CO_2CH_3]^+$ calcd. for $C_8H_{18}N$: 128.1, found: 128.0. **HRMS** (HESI): *m/z* $[M+H]^+$ calcd. for $C_{10}H_{22}NO_2$: 188.1645, found: 188.1644.

Methyl 2-(2-oxopropanamido)-3-propylhexanoate (17a)

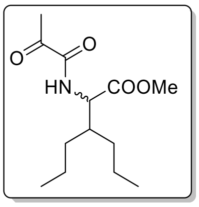

Colorless oil. **^{1}H NMR** (600 MHz, $CDCl_3$): δ 7.31 (d, *J* = 9.3 Hz, 1H), 4.66 (dd, *J* = 9.4, 3.7 Hz, 1H), 3.75 (s, 3H), 2.49 (s, 3H), 1.96 (m, 1H), 1.45 – 1.17 (m, 8H), 0.90 (t, *J* = 7.3 Hz, 6H) ppm. **^{13}C NMR** (100 MHz, $CDCl_3$): δ 196.39, 171.82, 159.92, 54.26, 52.37, 40.40, 32.85, 32.26, 24.44, 20.27, 20.24, 14.15, 14.12 ppm. **IR** (ATR): 3402, 2957, 2931, 2872, 1741, 1686, 1514, 1458, 1436, 1353, 1251, 1208, 1168, 1015, 917, 822, 734, 592 cm^{-1}. **MS** (APCI): *m/z* $[M+H]^+$ calcd. for $C_{13}H_{24}NO_4$: 258.2, found: 258.2; $[M-CO_2CH_3]^+$ calcd. for $C_{11}H_{20}NO_2$: 198.2, found: 198.2; $[M-H]^-$ calcd. for $C_{13}H_{22}NO_4$: 256.2, found: 256.1. **HRMS** (HESI): *m/z* $[M+H]^+$ calcd. for $C_{13}H_{24}NO_4$: 258.1700, found: 258.1699; $[M+Na]^+$ calcd. for $C_{13}H_{23}NO_4Na$: 280.1519, found: 280.1518.

Methyl (2-oxopropanoyl)-L-phenylalaninate (17b)

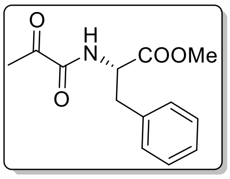

Colorless oil. **^{1}H NMR** (400 MHz, $CDCl_3$): δ 7.36 – 7.22 (m, 4H), 7.14 – 7.07 (m, 2H), 4.81 (dt, *J* = 8.5, 6.2 Hz, 1H), 3.72 (s, 3H), 3.14 (qd, *J* = 13.9, 6.1 Hz, 2H), 2.44 (s, 3H) ppm. **^{13}C NMR** (150 MHz, $CDCl_3$): δ 196.01, 170.91, 159.53, 135.29, 129.11 (2 x C_{Ar}), 128.73 (2 × C_{Ar}), 127.34, 53.22, 52.50, 37.89, 24.33 ppm. **IR** (ATR): 3339, 3028, 2953, 1741, 1680, 1517, 1498, 1437, 1355, 1248, 1219, 1197, 1165, 1105, 1079, 1030, 1016, 989, 746, 701, 609, 589 cm^{-1}. **MS** (APCI): *m/z* $[M+H]^+$ calcd. for $C_{13}H_{16}NO_4$: 250.1, found: 249.9; $[M-CO_2CH_3]^+$ calcd. for $C_{11}H_{12}NO_2$: 190.1, found: 189.8; $[M-H]^-$ calcd. for $C_{13}H_{14}NO_4$: 248.1, found: 247.6. **HRMS** (HESI): *m/z* $[M+H]^+$ calcd. for $C_{13}H_{16}NO_4$: 250.1074, found: 250.1073; $[M+Na]^+$ calcd. for $C_{13}H_{15}NO_4Na$: 272.0893, found: 272.0894.
Spectral data are fully consistent with those reported in the literature [1].

Methyl 2-(3-bromo-2-oxopropanamido)-3-propylhexanoate (18a)

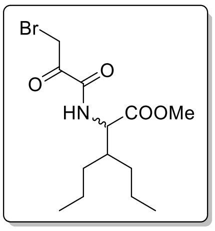

Colorless oil. The rotation of the amide bond is partially restricted which leads to the duplication of some signals. Based on the ^{1}H NMR, the isomeric ratio is 7:3. **^{1}H NMR** (600 MHz, $CDCl_3$): δ 7.36 (d, *J* = 9.2 Hz, 0.3H), 7.31 (d, *J* = 9.3 Hz, 0.7H), 4.72 (dd, *J* = 9.2, 3.7 Hz, 0.3H), 4.69

(dd, J = 9.3, 3.7 Hz, 0.7H), 4.49 (dd, J = 58.4, 30.9 Hz, 2H), 3.78 (s, 0.9H), 3.76 (s, 2.1H), 1.98 (m, 1H), 1.45 – 1.15 (m, 8H), 0.90 (t, J = 7.2 Hz, 6H) ppm. **^{13}C NMR** (100 MHz, $CDCl_3$): δ 188.98, 182.44, 171.50, 171.31, 158.19, 156.40, 54.77, 54.46, 52.58, 52.51, 40.58, 40.47, 36.99, 32.84, 32.82, 32.24, 30.10, 20.25, 20.23, 14.14, 14.11 ppm. **IR** (ATR): 3396, 2956, 2930, 2871, 1740, 1682, 1519, 1458, 1436, 1380, 1355, 1264, 1207, 1176, 1143, 1093, 1008, 938, 910, 734, 645 cm^{-1}. **MS** (APCI): *m/z* $[M+H]^+$ calcd. for $C_{17}H_{23}BrNO_4$: 336.1 and 338.1 (97.3%), found: 336.0 and 338.0; $[M-CO_2CH_3]^+$ calcd. for $C_{11}H_{19}BrNO_2$: 276.1 and 278.1, found: 275.9 and 277.9; $[M-H]^-$ calcd. for $C_{13}H_{21}BrNO_4$: 334.1 (100%) and 336.1, found: 333.8 and 335.7. **HRMS** (HESI): *m/z* $[M+H]^+$ calcd. for $C_{13}H_{23}BrNO_4$: 336.0805 and 338.0785, found: 336.0805 and 338.0784; $[M+Na]^+$ calcd. for $C_{13}H_{22}BrNO_4Na$: 358.0624 and 360.0604, found: 358.0624 and 360.0603.

Methyl (3-bromo-2-oxopropanoyl)-L-phenylalaninate (18b)

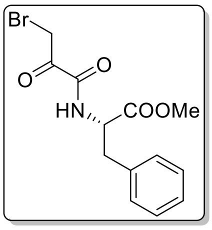

Yellow oil. **^{1}H NMR** (400 MHz, $CDCl_3$): δ 7.37 – 7.25 (m, 4H), 7.14 – 7.08 (m, 2H), 4.84 (ddd, J = 8.4, 6.5, 5.7 Hz, 1H), 4.44 (dd, J = 38.7, 11.4 Hz, 2H), 3.74 (s, 3H), 3.24 – 3.09 (m, 2H) ppm. **^{13}C NMR** (100 MHz, $CDCl_3$): δ 188.68, 170.64, 157.86, 135.08, 129.15, 128.82, 127.47, 53.41, 52.67, 37.77, 29.97 ppm. **IR** (ATR): 3346, 3060, 3028, 2951, 1812, 1738, 1676, 1520, 1497, 1437, 1363, 1271, 1205, 1176, 1120, 1081, 1047, 1030, 911, 882, 732, 700, 648, 594, 487 cm^{-1}. **MS** (APCI): *m/z* $[M+H]^+$ calcd. for $C_{13}H_{15}BrNO_4$: 328.0 (100%) and 329.0 (97.3%), found: 327.7 and 329.6; $[M-COOCH_3]^+$ calcd. for $C_{11}H_{11}BrNO_2$: 268.0 and 270.0, found: 267.7 and 269.7. **HRMS** (HESI): *m/z* $[M+H]^+$ calcd. for $C_{13}H_{15}BrNO_4$: 328.0179 and 330.0159, found: 328.0178 and 330.0158; $[M+Na]^+$ calcd. for $C_{13}H_{14}BrNO_4Na$: 349.9998 and 351.9978, found: 349.9997 and 351.9976.

Methyl 2-(2*H*-benzo[*b*][1,4]thiazine-3-carboxamido)-3-propylhexanoate (19a)

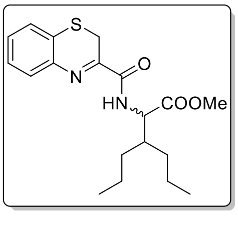

Yellow oil. **^{1}H NMR** (600 MHz, $CDCl_3$): δ 8.02 (d, J = 9.3 Hz, 1H), 7.47 (dd, J = 7.8, 1.5 Hz, 1H), 7.31 (dd, J = 7.7, 1.5 Hz, 1H), 7.23 (td, J = 7.6, 1.6 Hz, 1H), 7.19 (td, J = 7.5, 1.5 Hz, 1H), 4.81 (dd, J = 9.3, 3.9 Hz, 1H), 3.76 (s, 3H), 3.64 (dd, J = 90.1, 15.2 Hz, 2H), 2.01 (m, 1H), 1.48 – 1.25 (m, 9H), 0.91 (m, 6H) ppm. **^{13}C NMR** (100 MHz, $CDCl_3$): δ 172.49, 162.64, 150.41, 141.83, 129.12, 128.80, 127.32, 126.53, 125.20, 54.60, 52.28, 40.52, 32.91, 32.36, 20.30 (2 × C), 19.97, 14.22, 14.18 ppm. **IR** (ATR): 3360, 2956, 2928, 2871, 1738, 1670, 1618, 1592, 1512, 1477, 1465, 1437, 1376, 1351, 1314, 1261, 1203, 1154, 1028, 908, 731, 647 cm^{-1}. **MS** (APCI): *m/z* $[M+H]^+$ calcd. for $C_{19}H_{27}N_2O_3S$: 363.2, found: 363.1; $[M-CO_2CH_3]^+$ calcd. for

$C_{17}H_{23}N_2O_3S$: 303.2, found: 303.1; $[M-H]^-$ calcd. for $C_{19}H_{25}N_2O_3S$: 361.2, found: 361.0. **HRMS** (HESI): *m/z* $[M+H]^+$ calcd. for $C_{19}H_{27}N_2O_3S$: 363.1737, found: 363.1737; $[M+Na]^+$ calcd. for $C_{19}H_{26}N_2O_3SNa$: 385.1556, found: 385.1556.

Methyl (2*H*-benzo[*b*][1,4]thiazine-3-carbonyl)-L-phenylalaninate (19b)

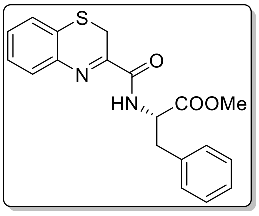

Yellow oil. **^{1}H NMR** (400 MHz, $CDCl_3$): δ 8.02 (d, *J* = 8.4 Hz, 1H), 7.41 (m, 1H), 7.34 – 7.26 (m, 4H), 7.25 – 7.16 (m, 4H), 4.96 (dt, *J* = 8.4, 6.2 Hz, 1H), 3.74 (s, 3H), 3.62 (q, *J* = 15.2 Hz, 2H), 3.21 (dd, *J* = 6.2, 1.7 Hz, 2H) ppm. **^{13}C NMR** (100 MHz, $CDCl_3$): δ 171.56, 162.33, 150.20, 141.76, 135.74, 129.28, 129.07, 128.85, 128.67, 127.33, 127.25, 126.55, 125.15, 53.59, 52.41, 38.25, 19.88 ppm. **IR** (ATR): 3378, 3058, 3027, 3001, 2950, 2928, 2850. 1740, 1670, 1616, 1508, 1442, 1410, 1364, 1304, 1262, 1211, 1174, 1127, 1077, 1057, 1030, 909, 843, 763, 730, 701, 650, 598, 541,494, 466, 437 cm^{-1}. **MS** (APCI): *m/z* $[M+H]^+$ calcd. for $C_{19}H_{19}N_2O_3S$: 355.1, found: 354.8; $[M-CO_2CH_3]^+$ calcd. for $C_{17}H_{15}N_2O_3S$: 295.1, found: 294.8; $[M-H]^-$ calcd. for $C_{19}H_{17}N_2O_3S$: 353.1, found: 352.5. **HRMS** (HESI): *m/z* $[M+H]^+$ calcd. for $C_{19}H_{19}N_2O_3S$: 355.1111, found: 355.1110; $[M+Na]^+$ calcd. for $C_{19}H_{18}N_2O_3SNa$: 377.0930, found: 377.0930; $[M+K]^+$ calcd. for $C_{19}H_{18}N_2O_3SK$: 393.0670, found: 393.0668.

5. Pictures of NMR, MS, HRMS, and HPLC spectra

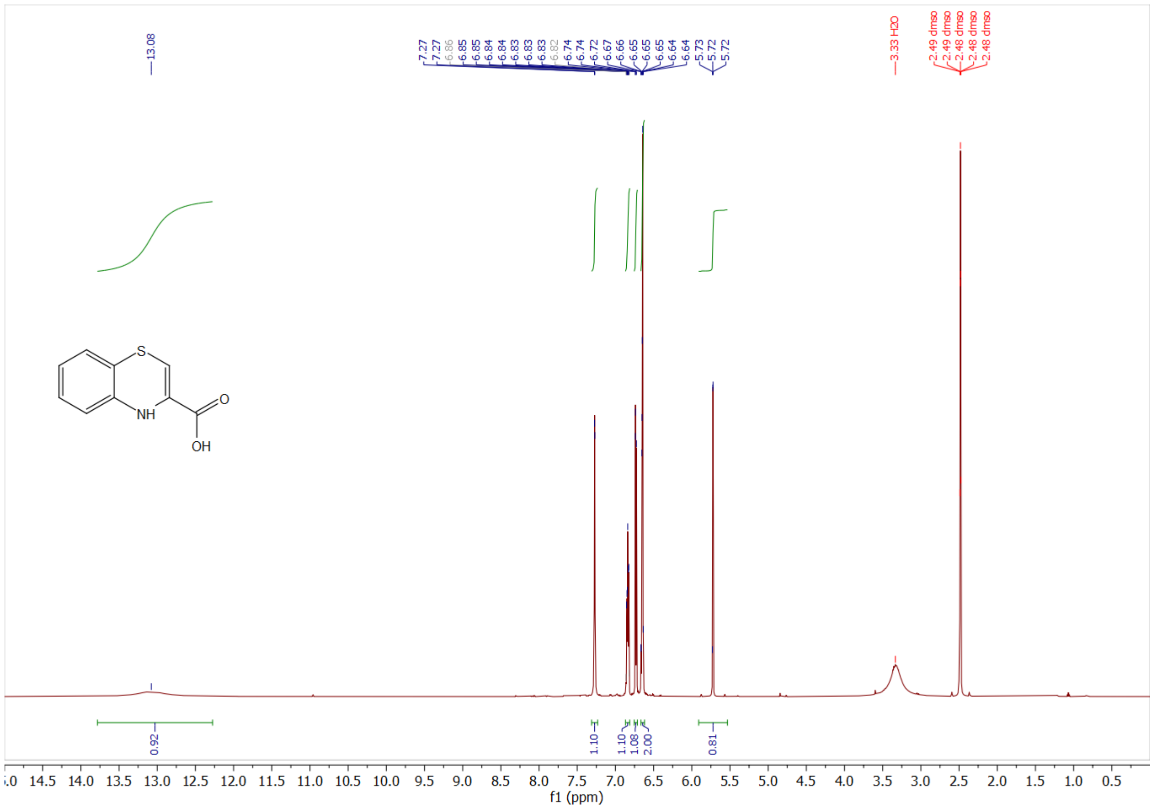

Figure S1. ^{1}H NMR spectrum of compound **10aa**.

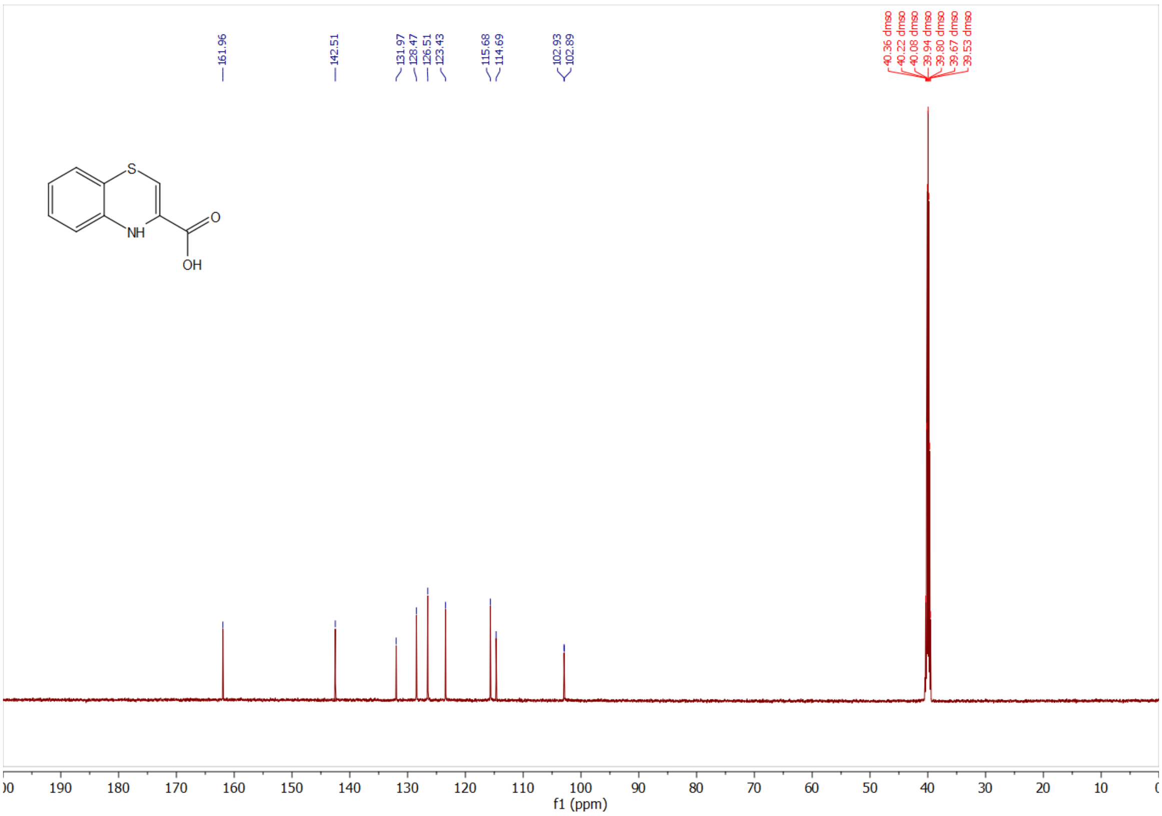

Figure S2. ^{13}C NMR spectrum of compound **10aa**.

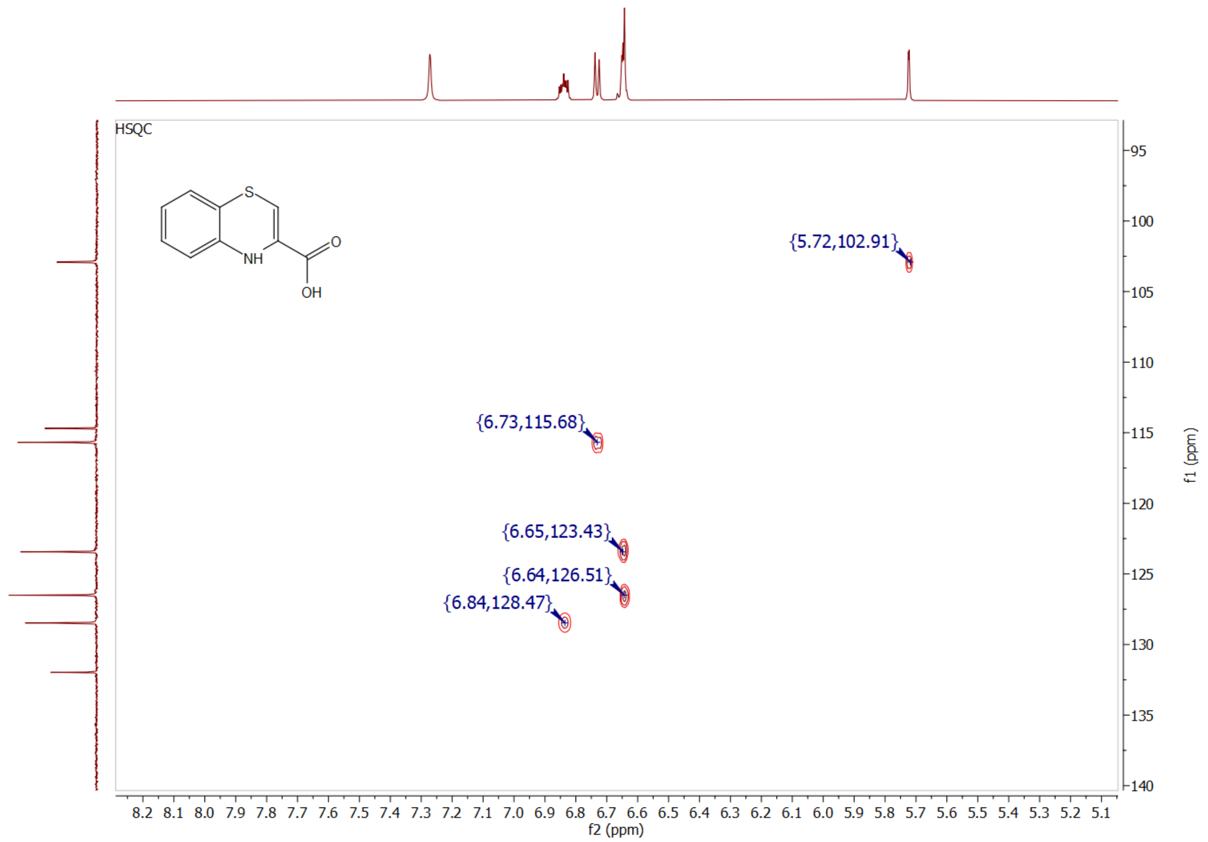

Figure S3. HSQC spectrum of compound **10aa**.

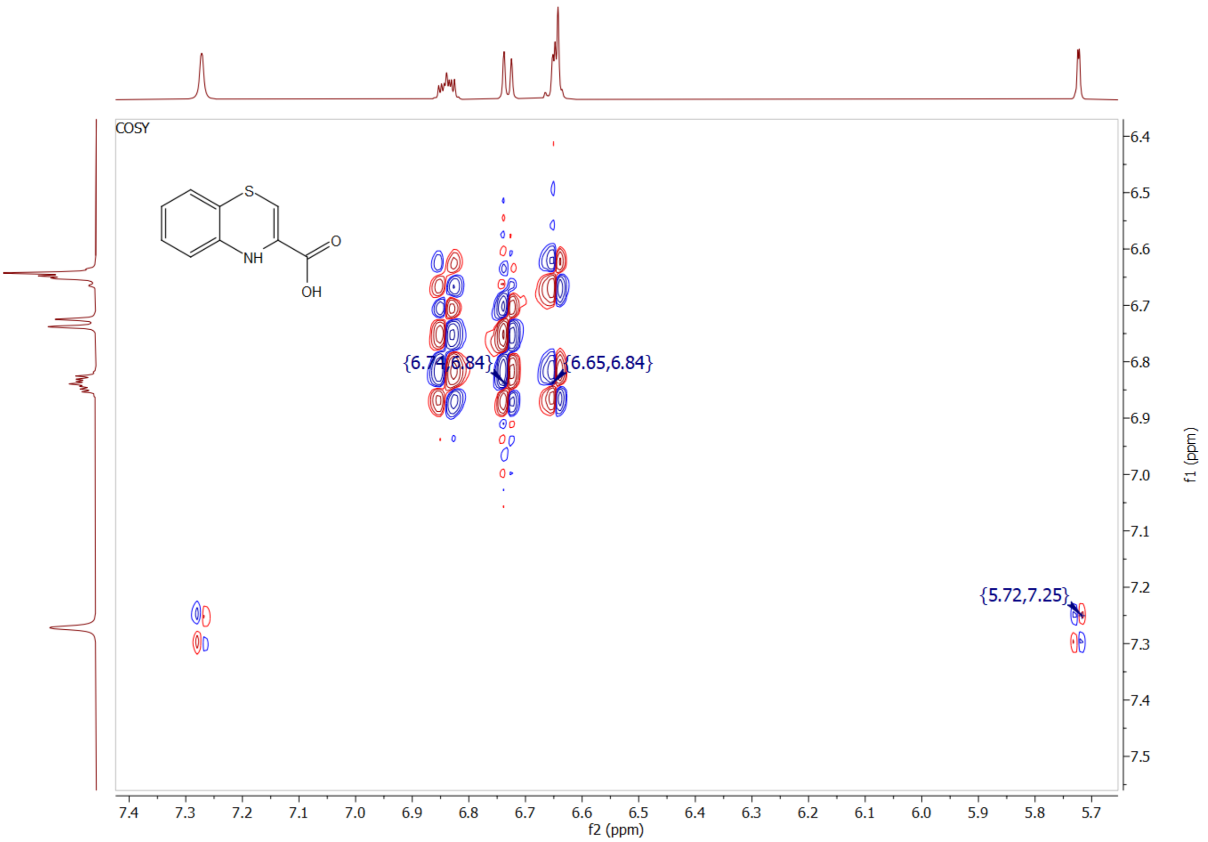

Figure S4. COSY spectrum of compound **10aa**.

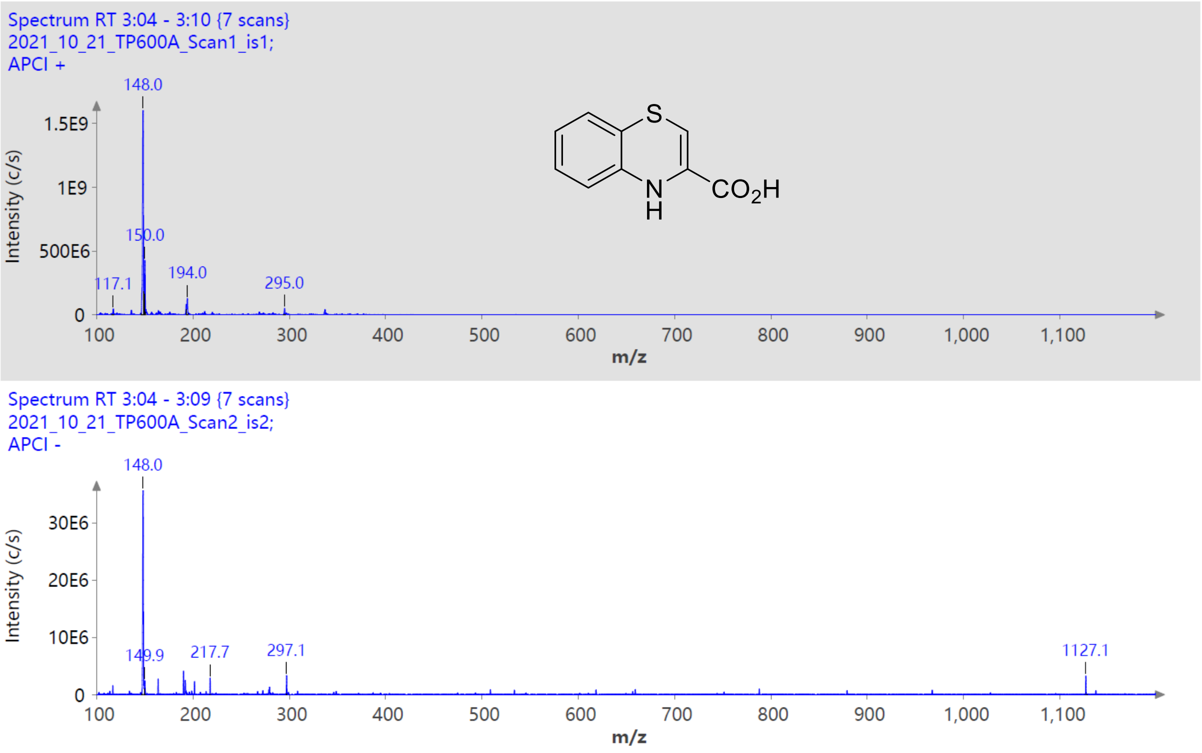

Figure S5. MS spectrum of compound **10aa**.

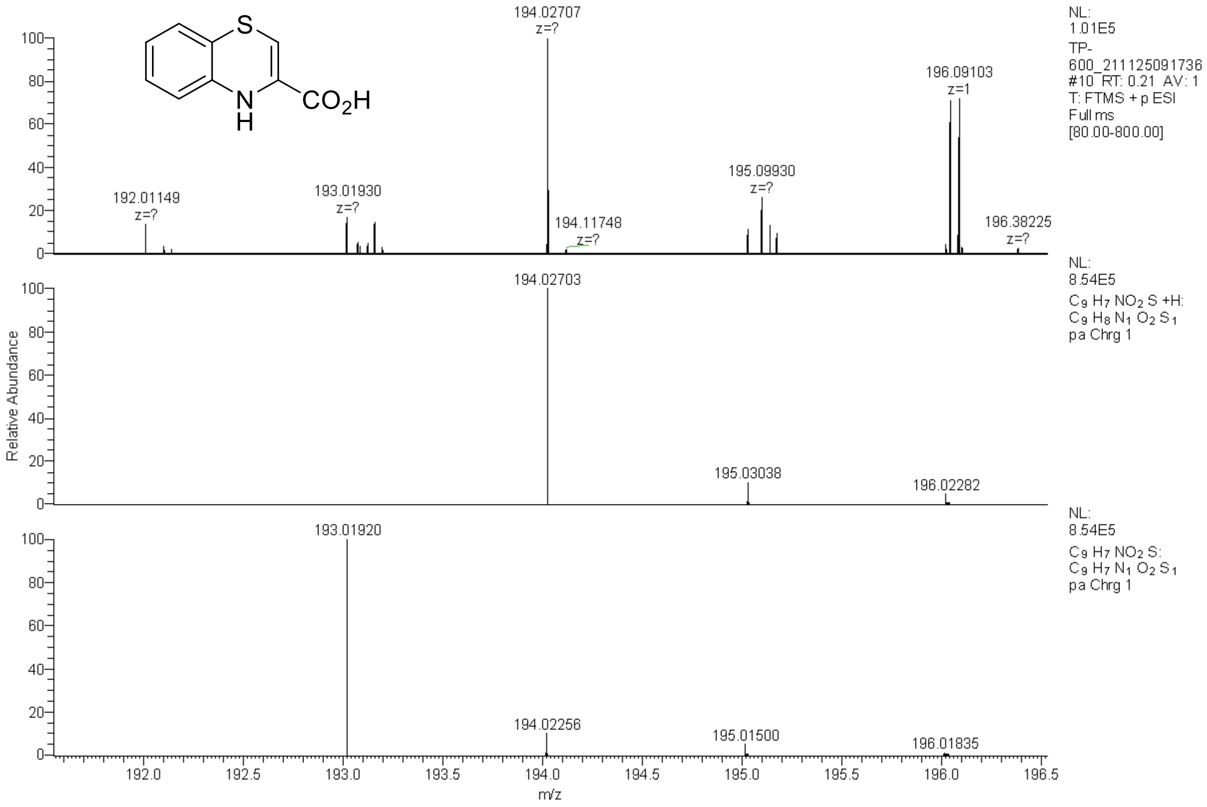

Figure S6. HRMS spectrum of compound **10aa**.

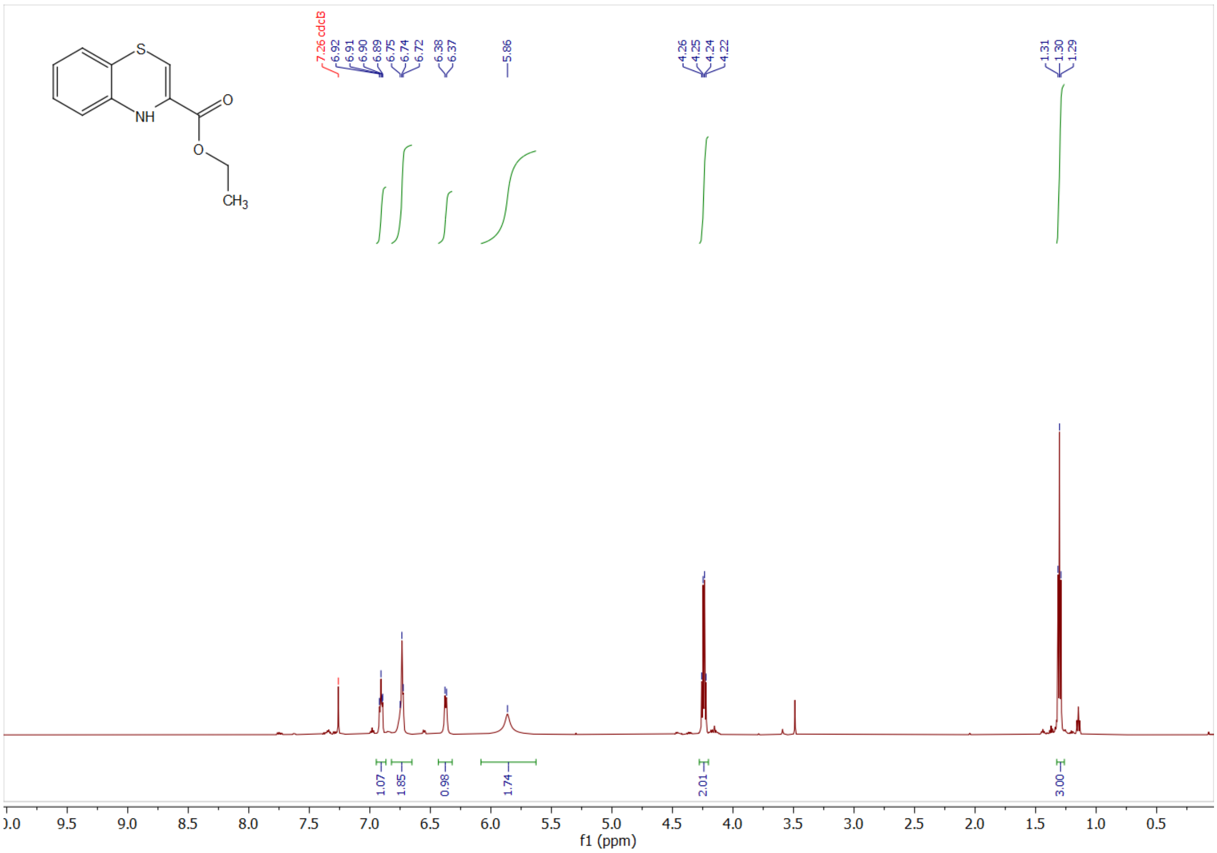

Figure S7. ^{1}H NMR spectrum of compound **10ac**.

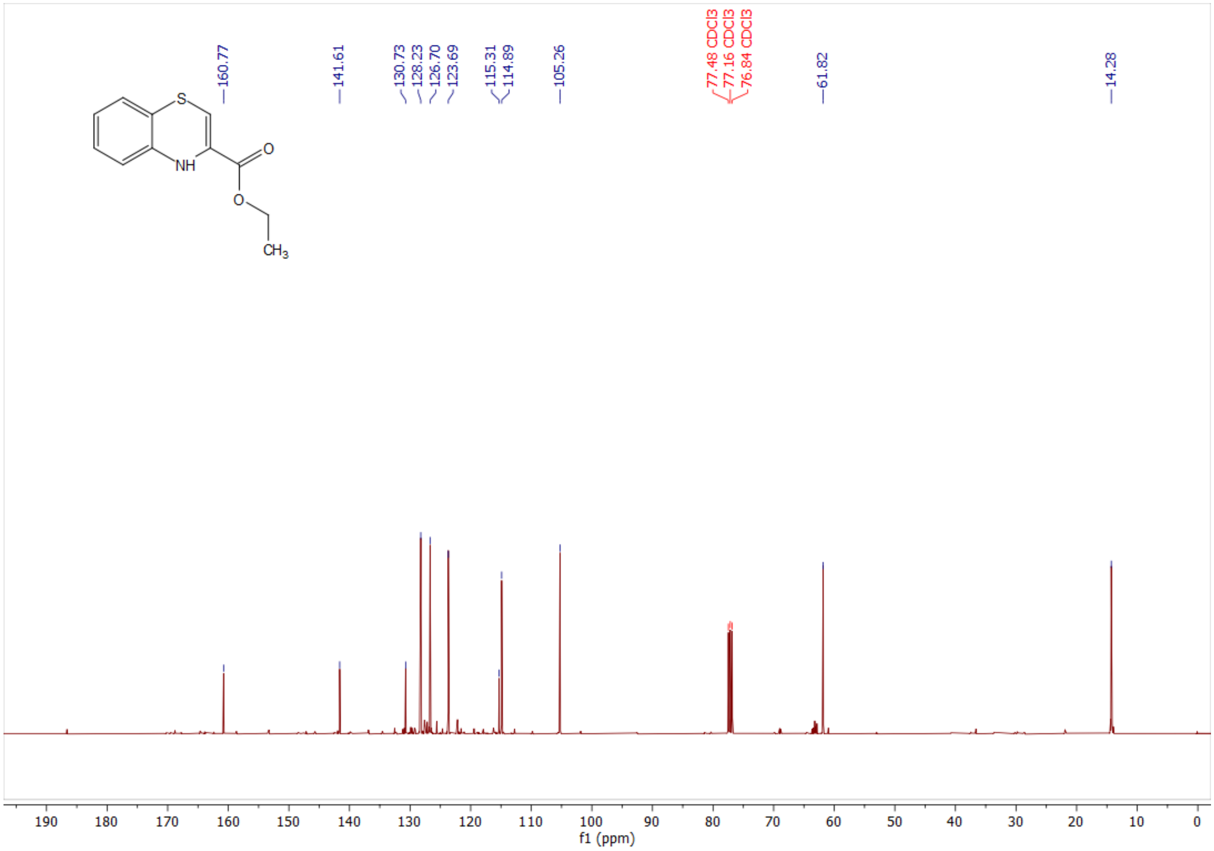

Figure S8. ^{13}C NMR spectrum of compound **10ac**.

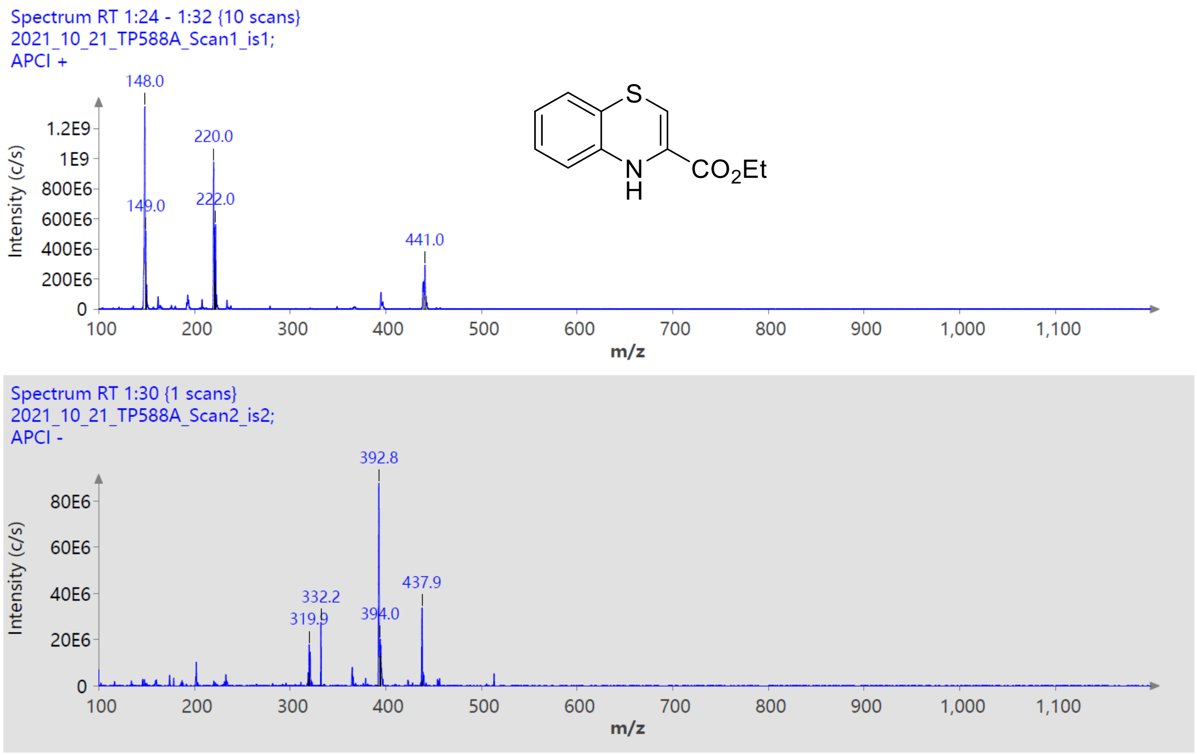

Figure S9. MS spectrum of compound **10ac**.

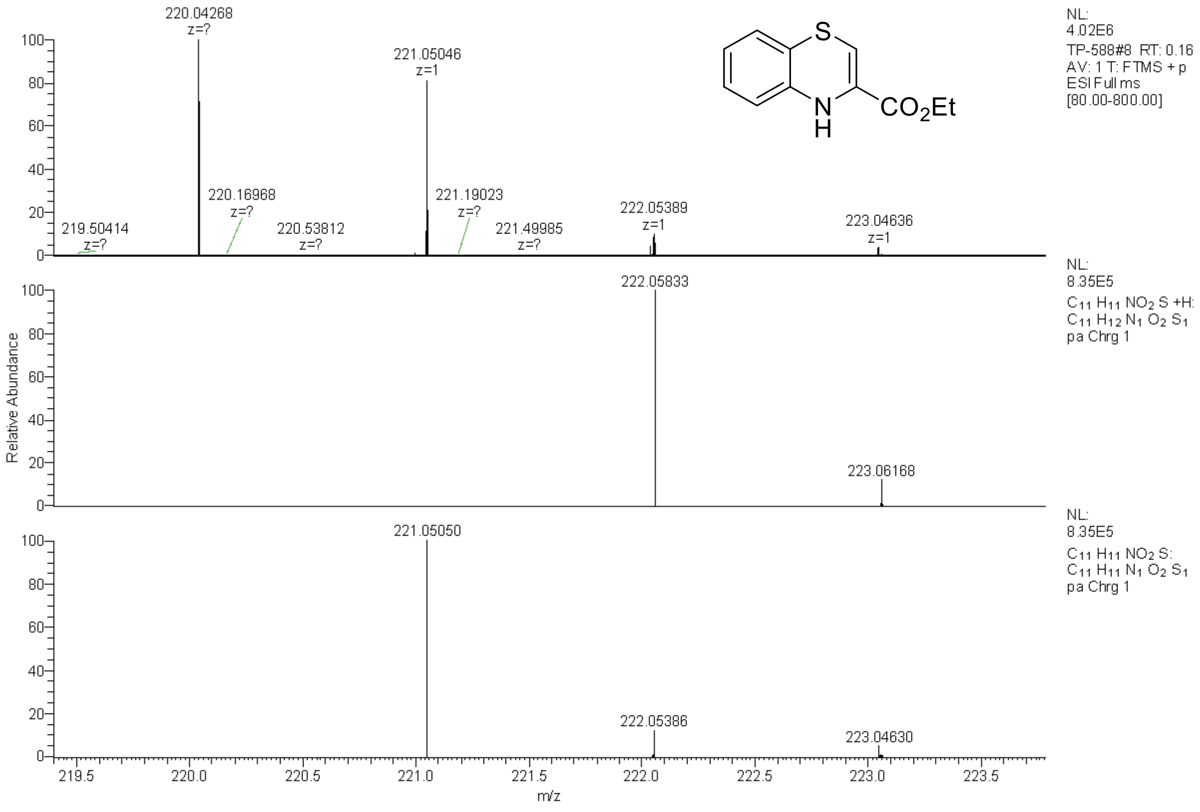

Figure S10. HRMS spectrum of compound **10ac**.

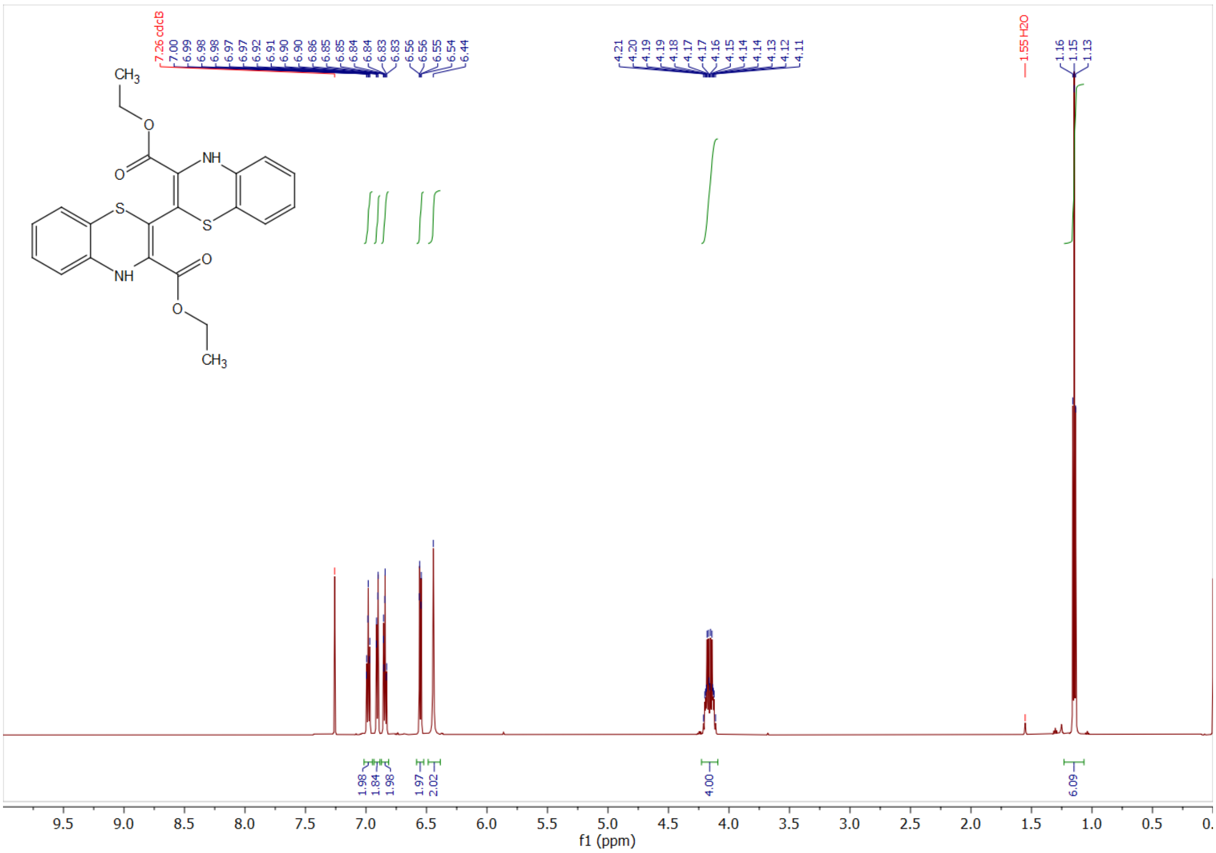

Figure S11. ^{1}H NMR spectrum of compound **11a**.

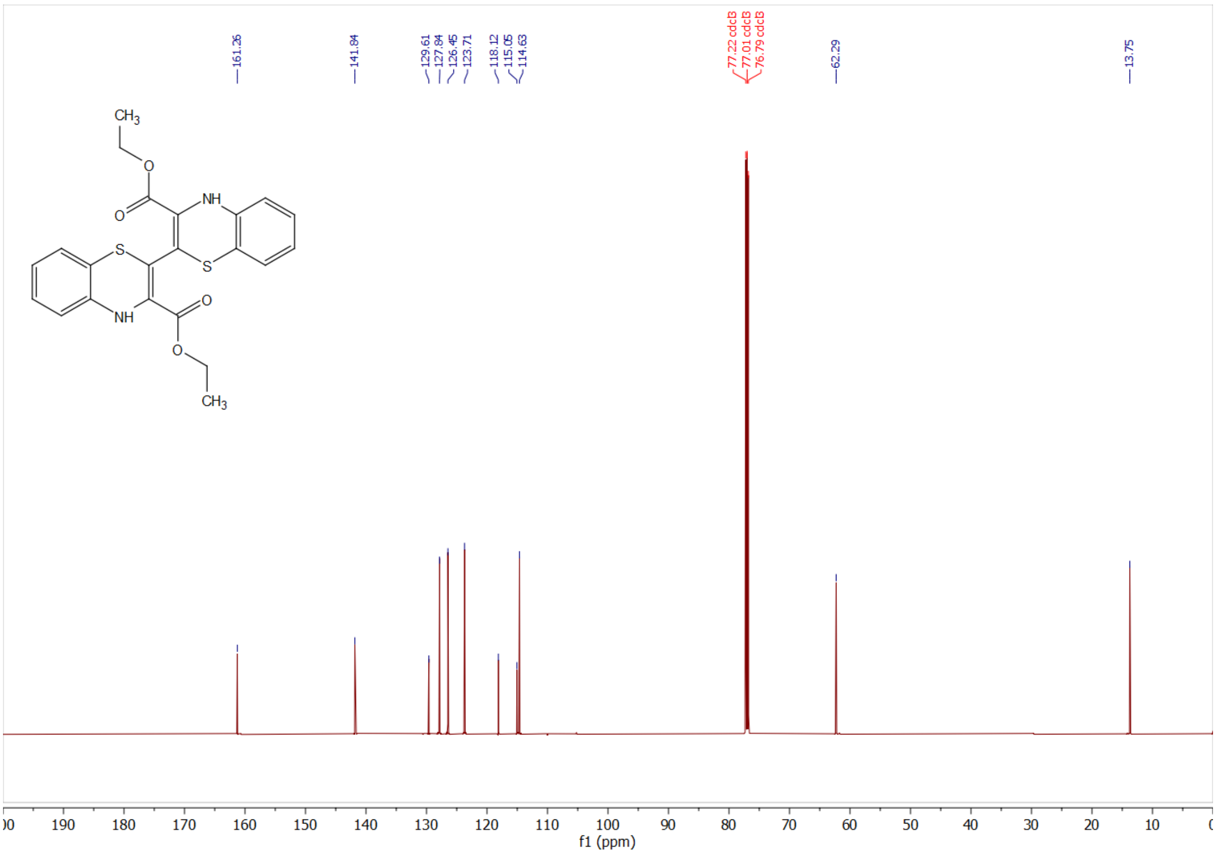

Figure S12. ^{13}C NMR spectrum of compound **11a**.

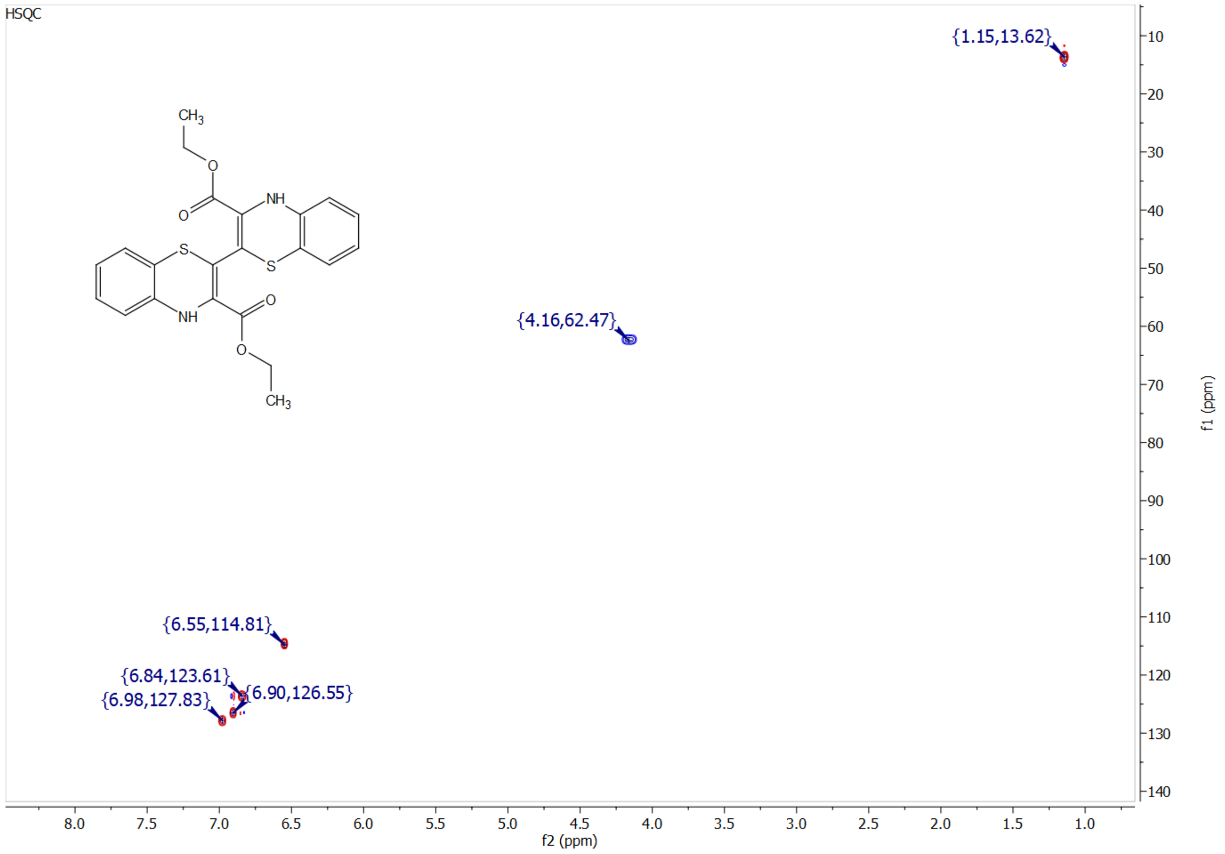

Figure S13. HSQC spectrum of compound **11a**.

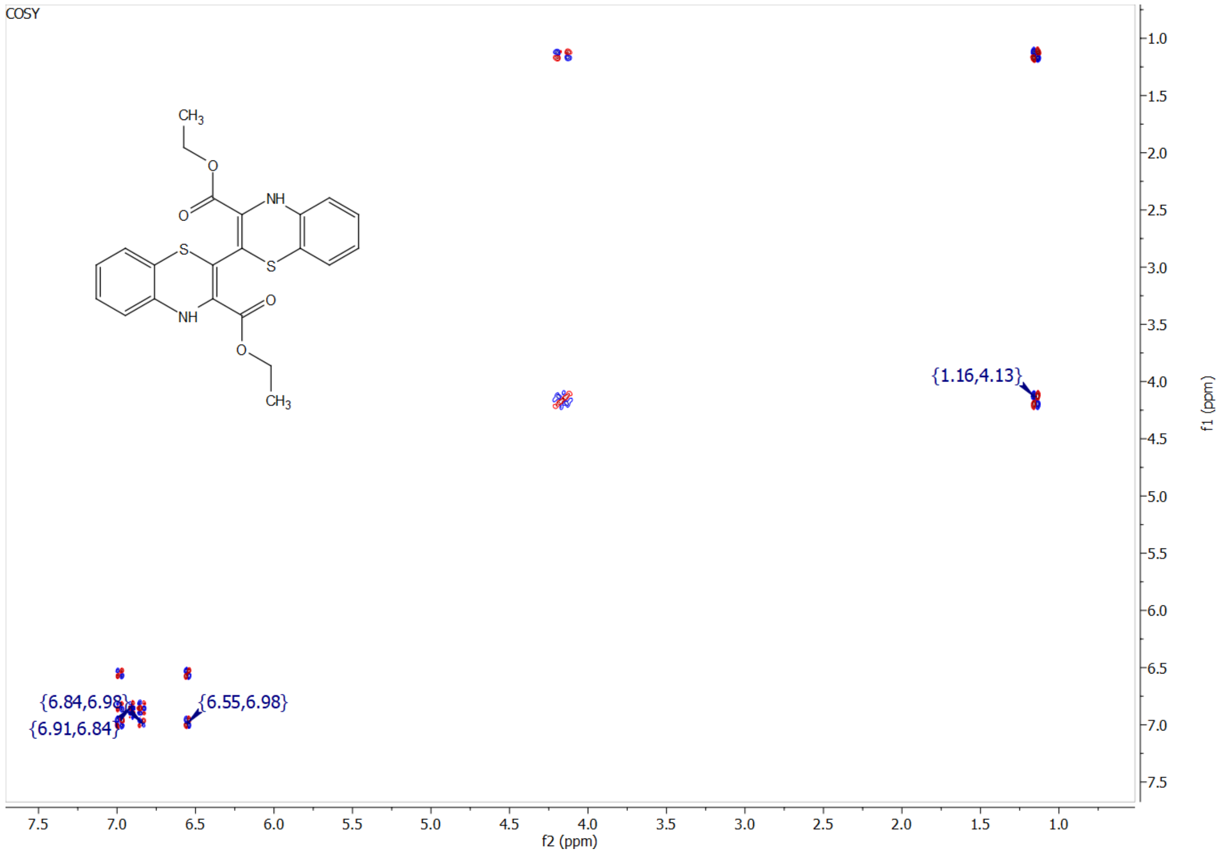

Figure S14. COSY spectrum of compound **11a**.

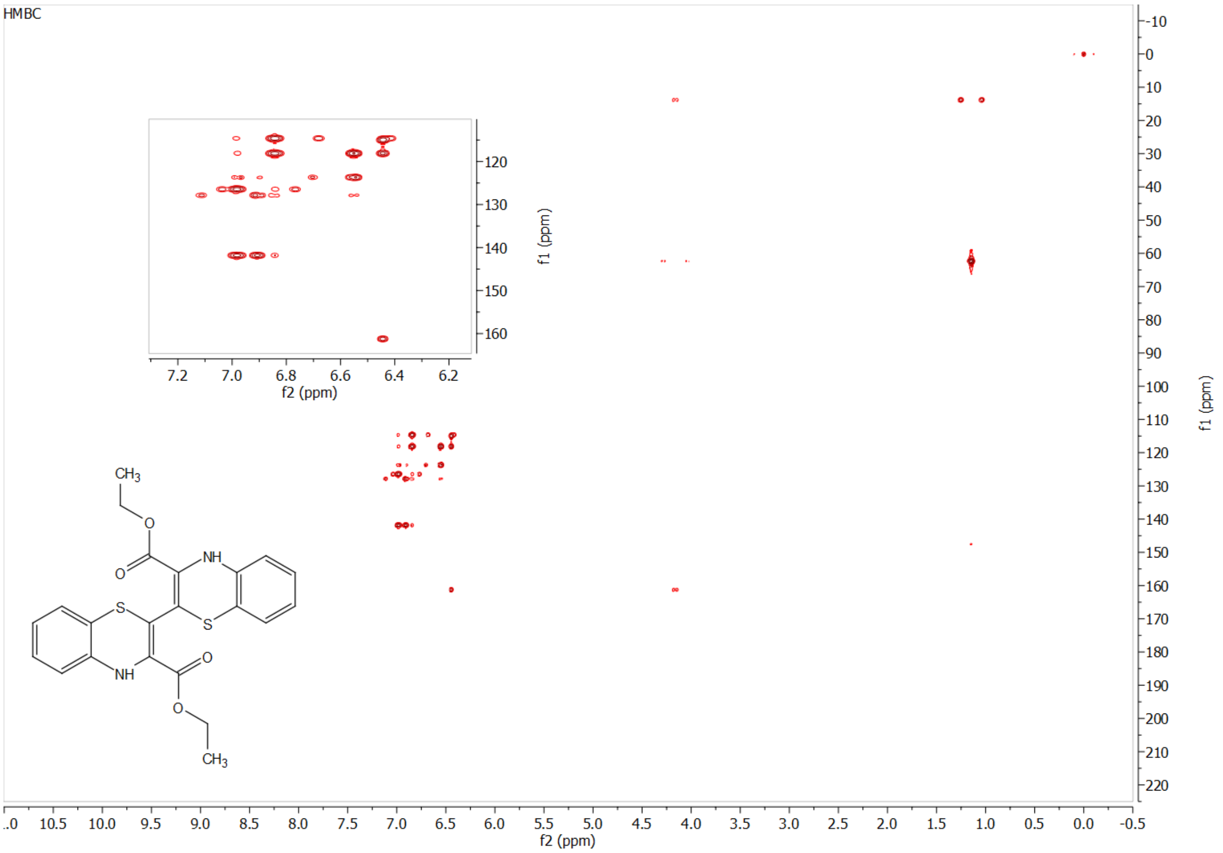

Figure S15. HMBC spectrum of compound **11a**.

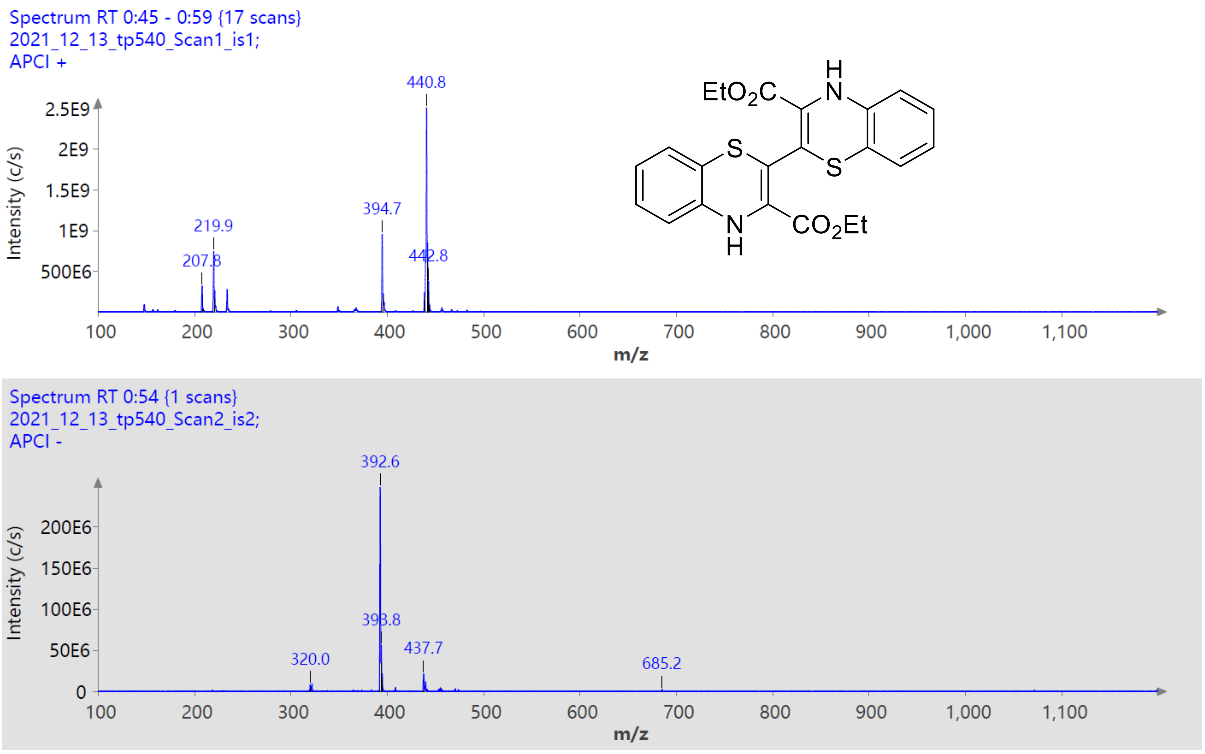

Figure S16. MS spectrum of compound **11a**.

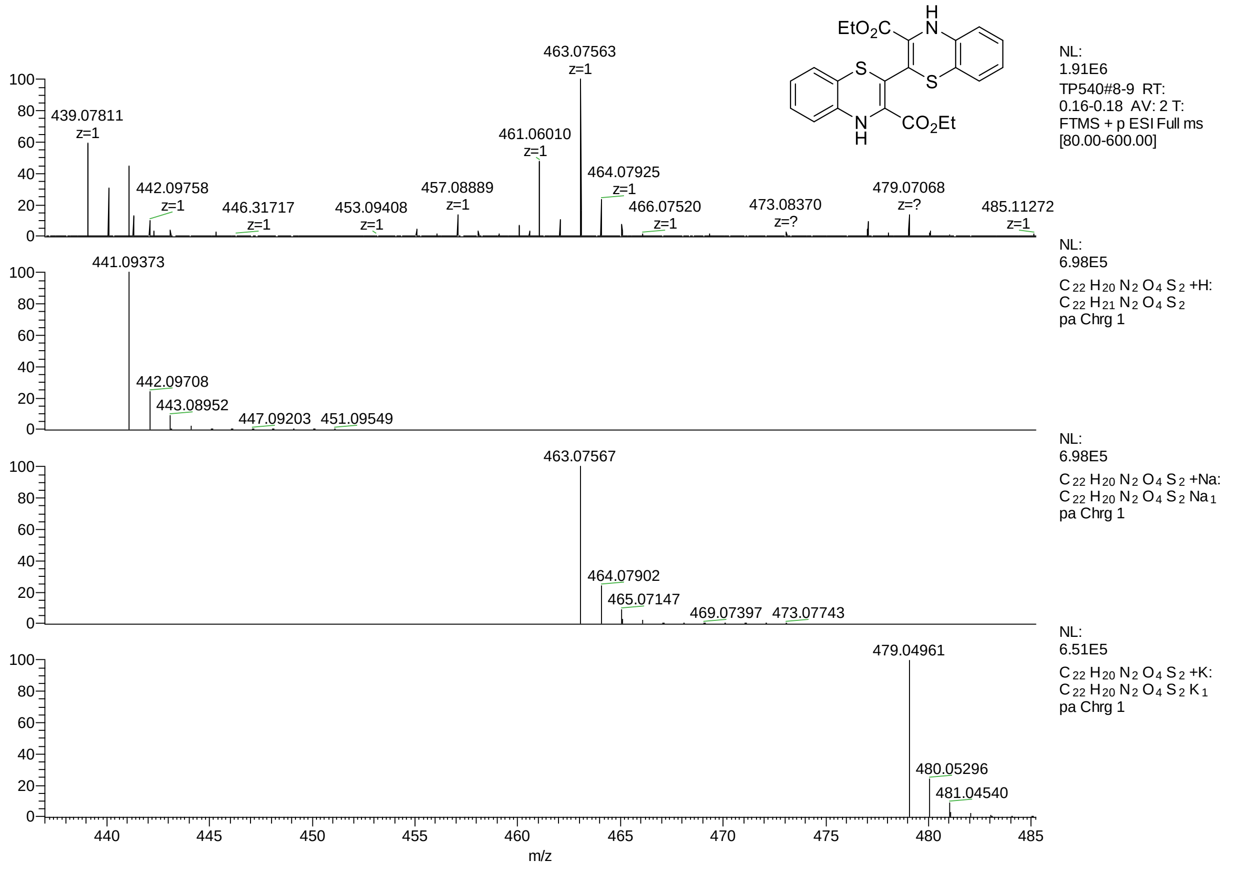

Figure S17. HRMS spectrum of compound **11a**.

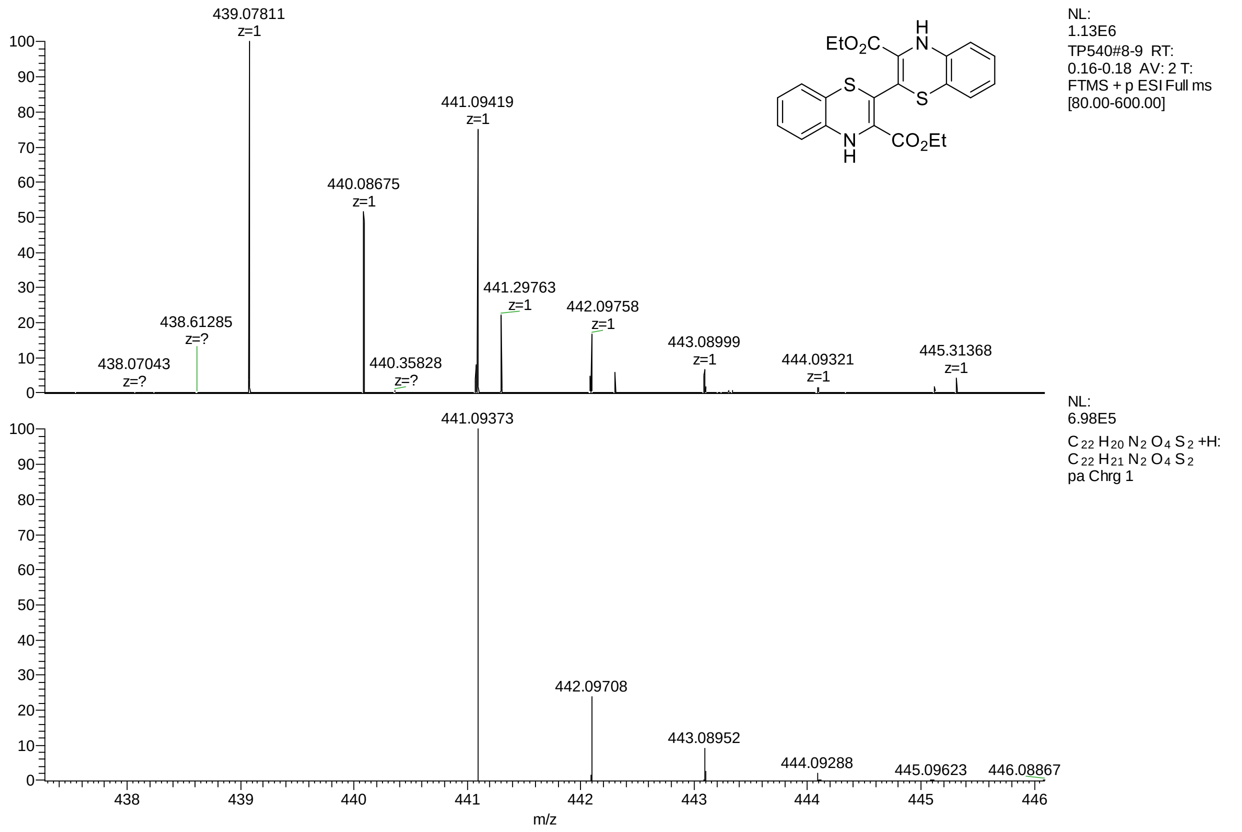

Figure S18. HRMS spectrum of compound **11a** (enlarged for $[M+H]^+$).

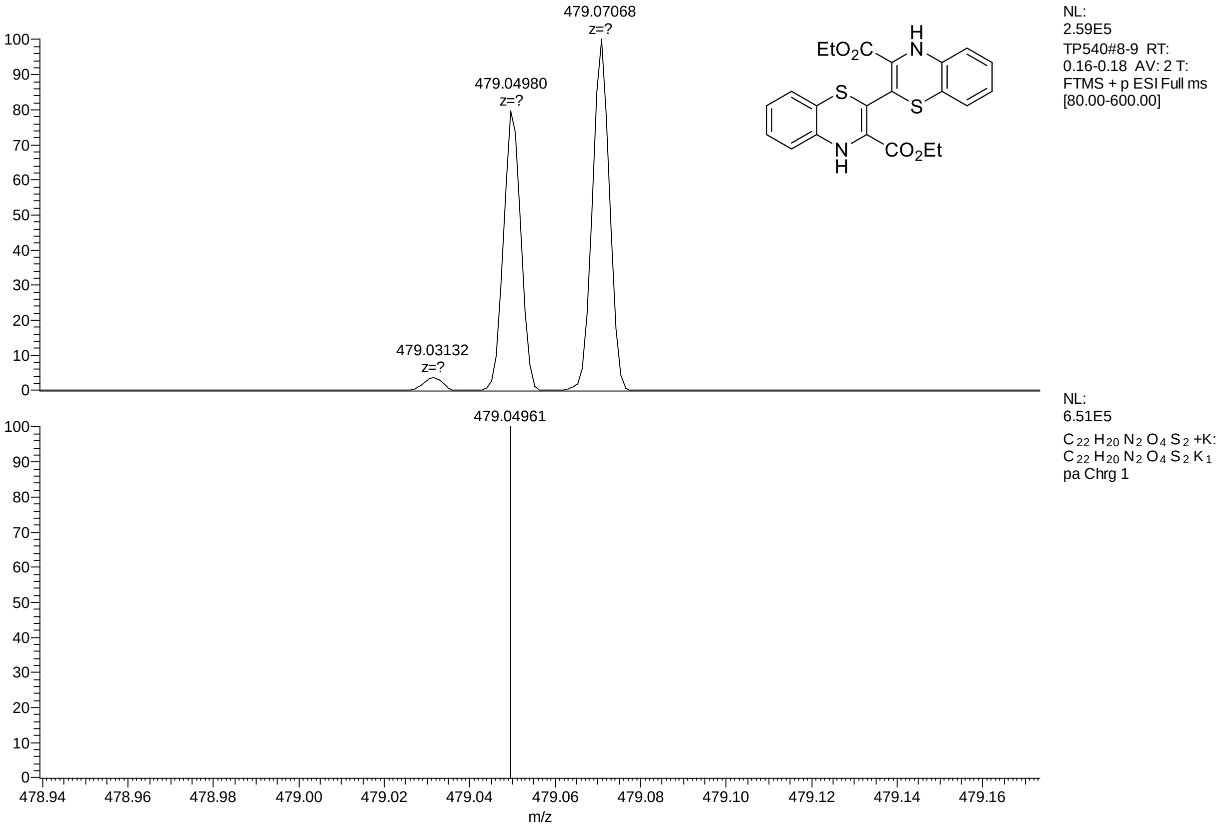

Figure S19. HRMS spectrum of compound **11a** (enlarged for [M+K]$^{+}$).

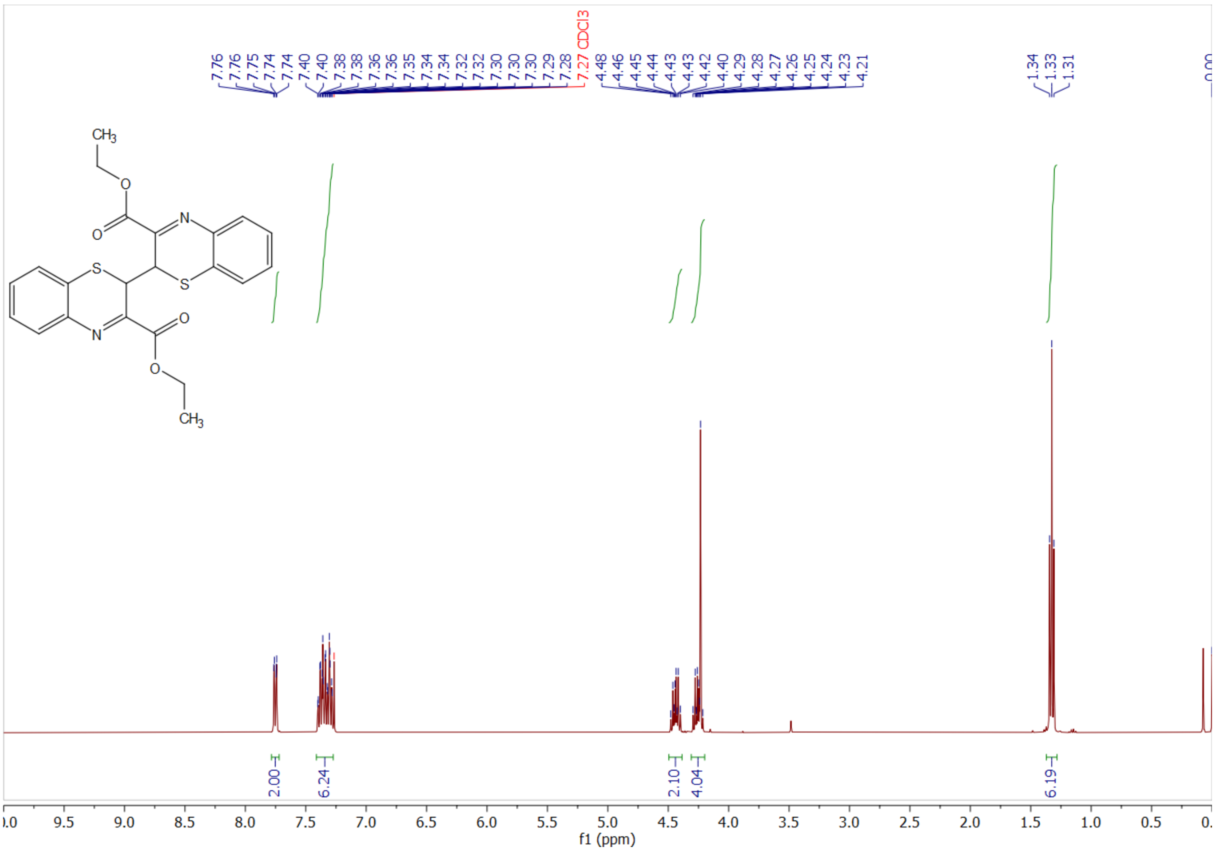

Figure S20. ^{1}H NMR spectrum of compound **11b**.

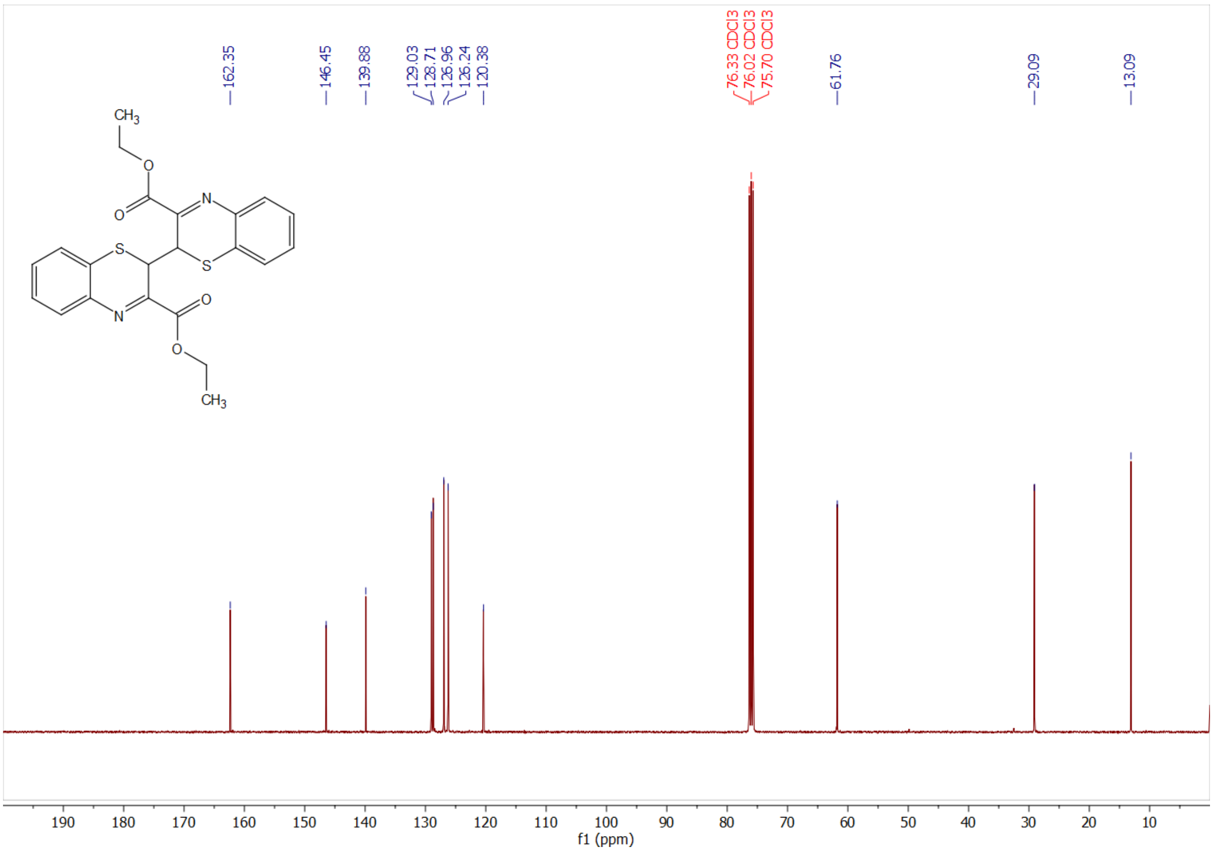

Figure S21. ^{13}C NMR spectrum of compound **11b**.

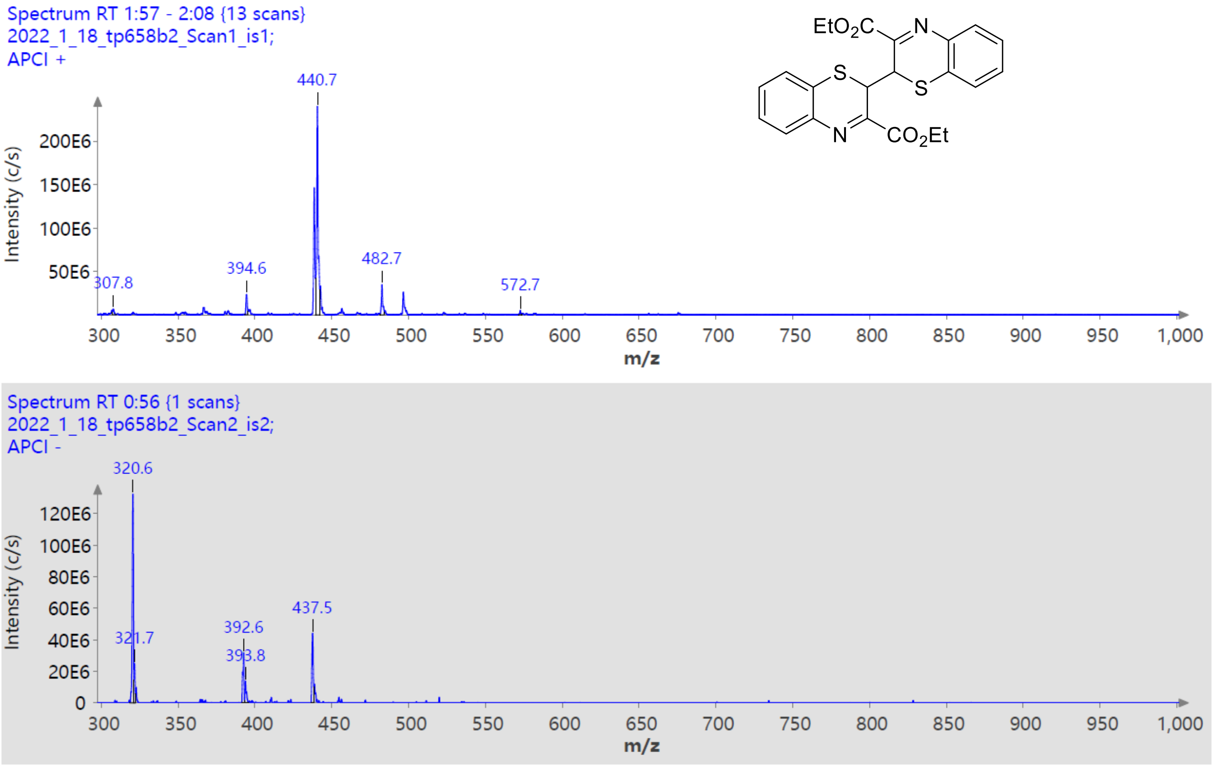

Figure S22. MS spectrum of compound **11b**.

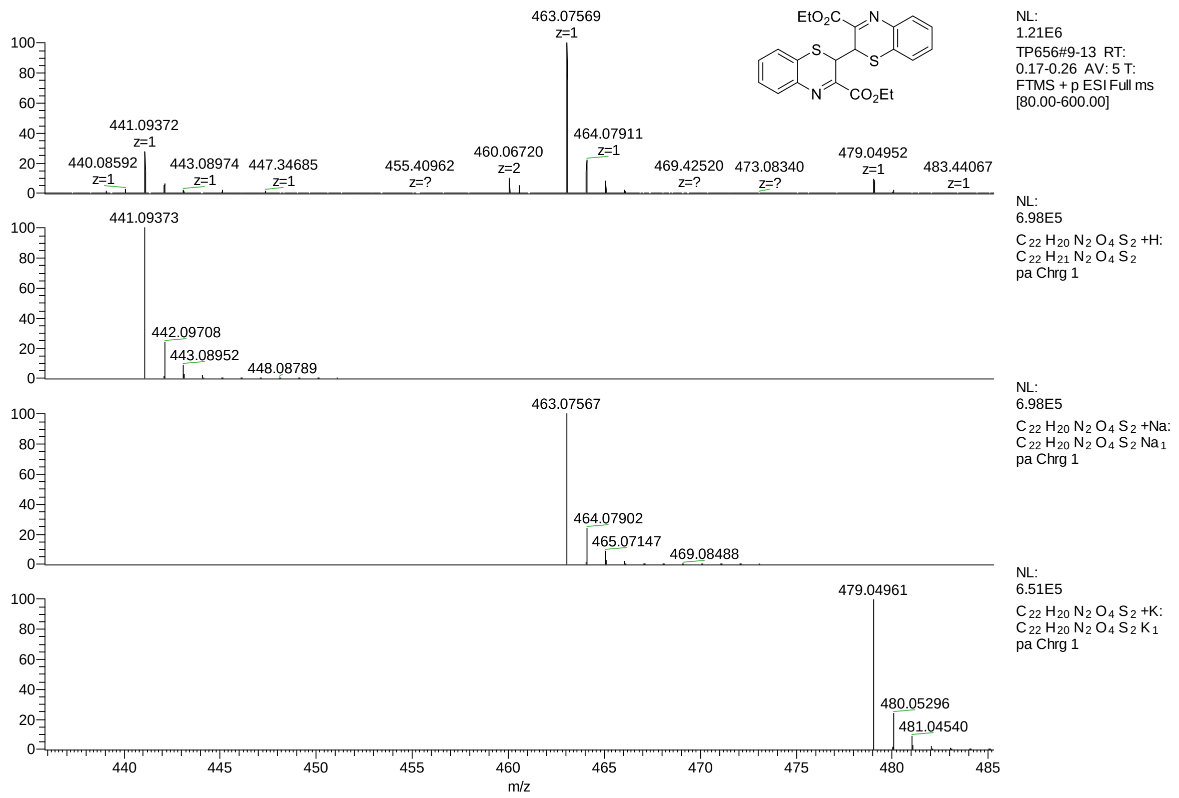

Figure S23. HRMS spectrum of compound **11b**.

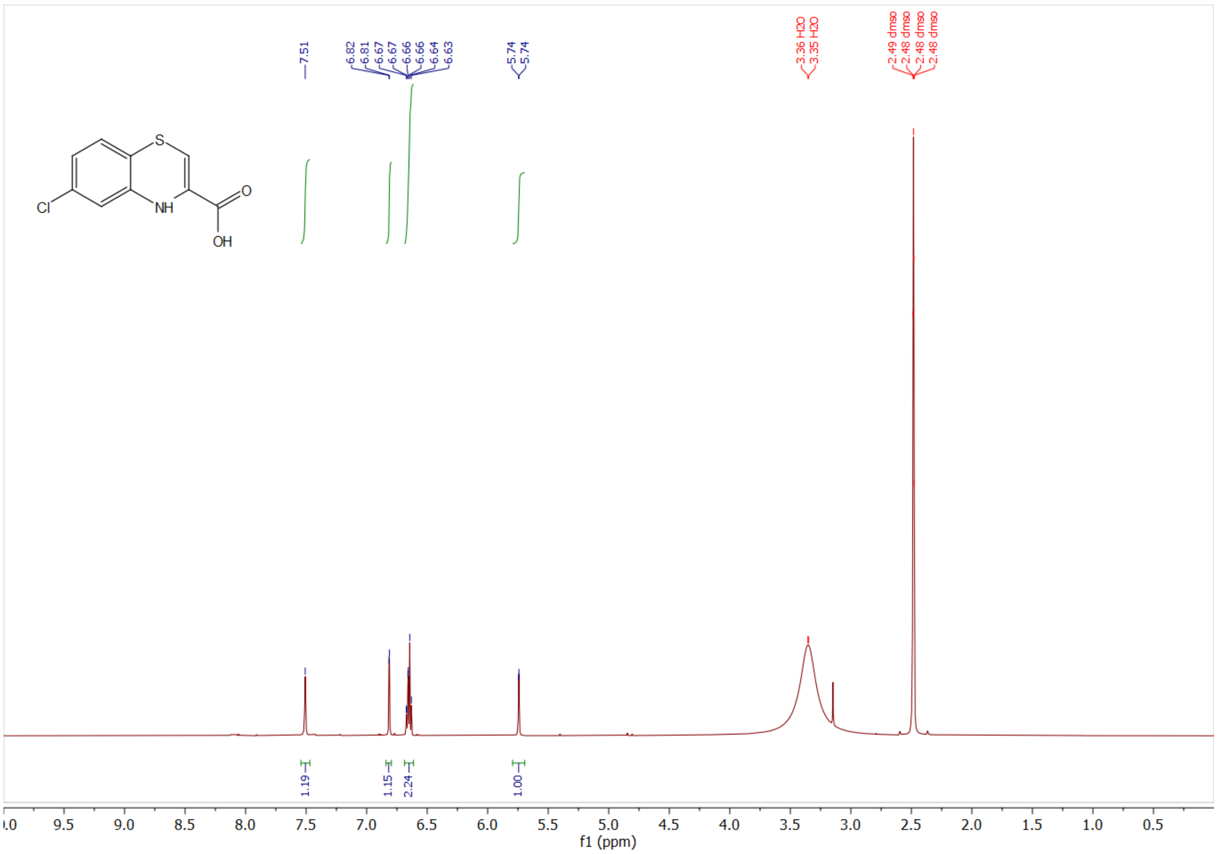

Figure S24. ^{1}H NMR spectrum of compound **10ba**.

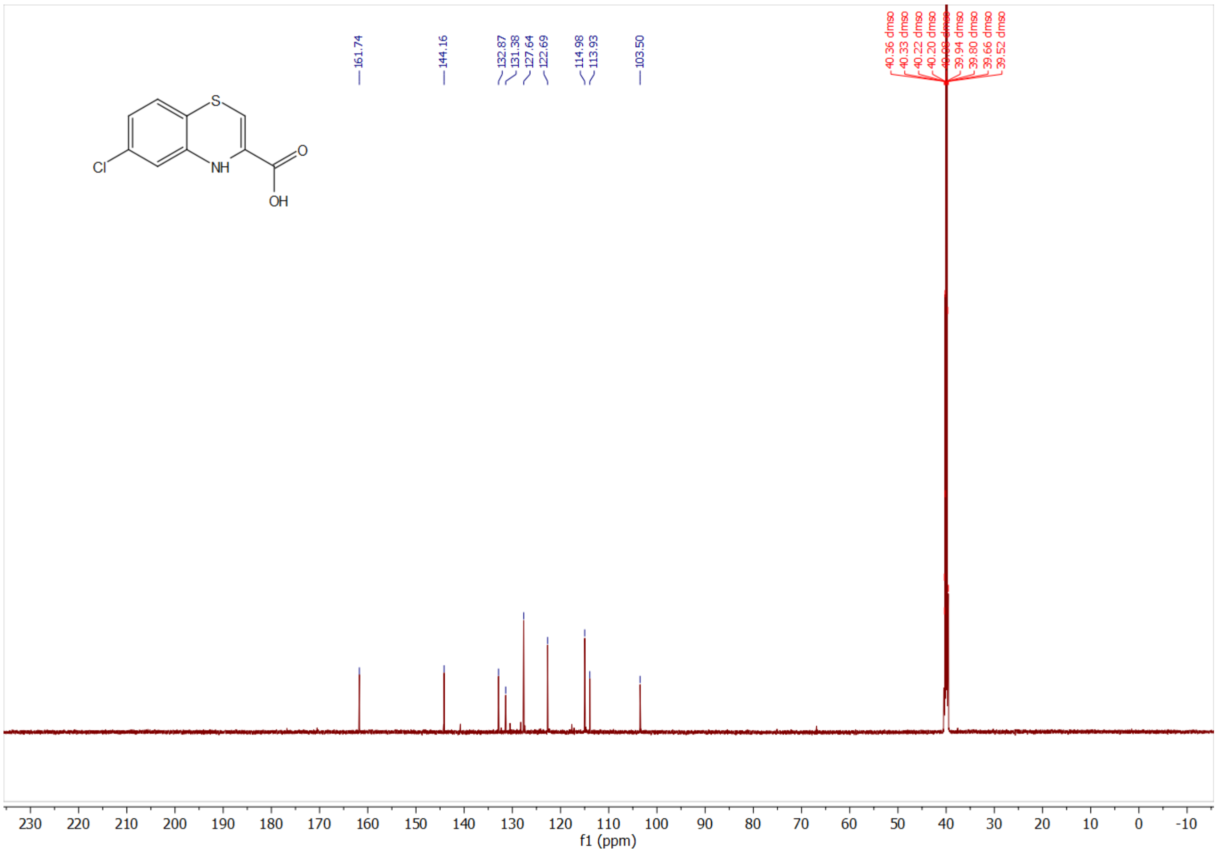

Figure S8. ^{13}C NMR spectrum of compound **10ba**.

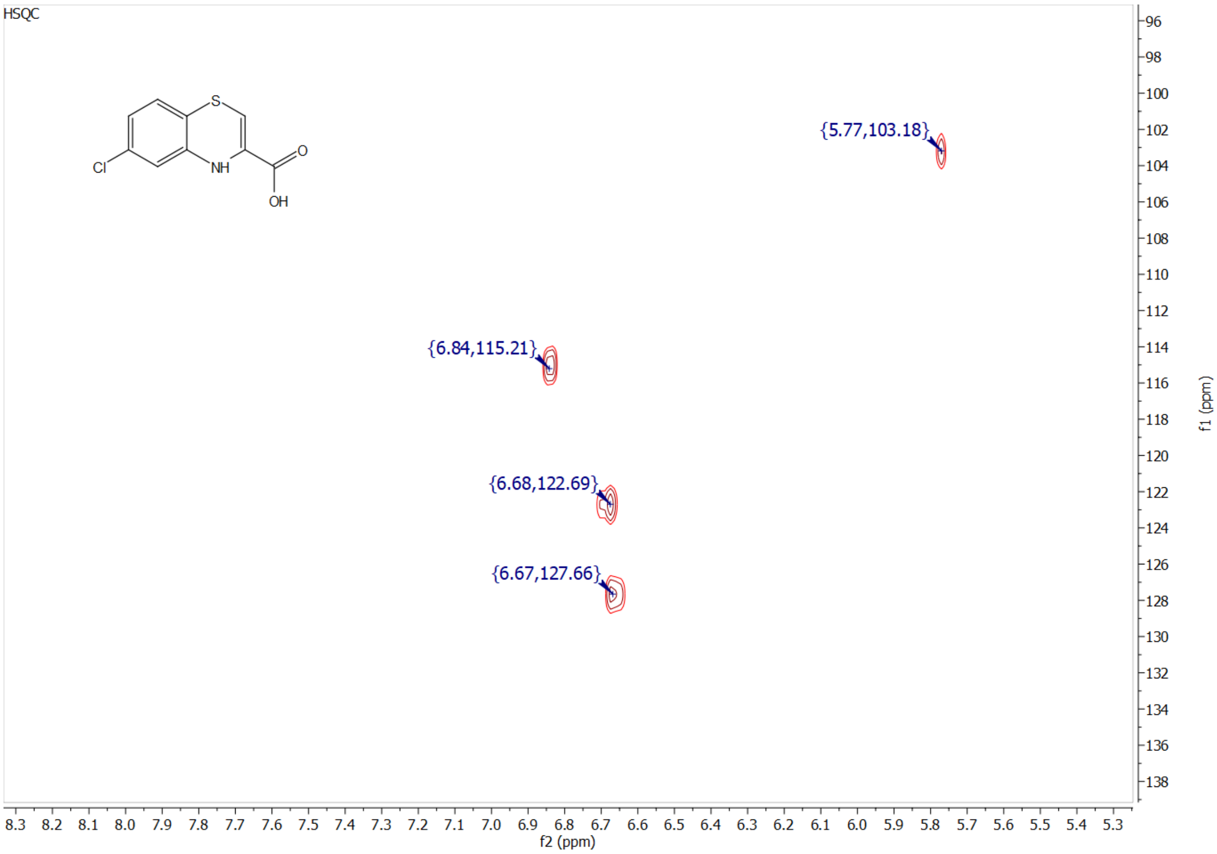

Figure S9. HSQC spectrum of compound **10ba**.

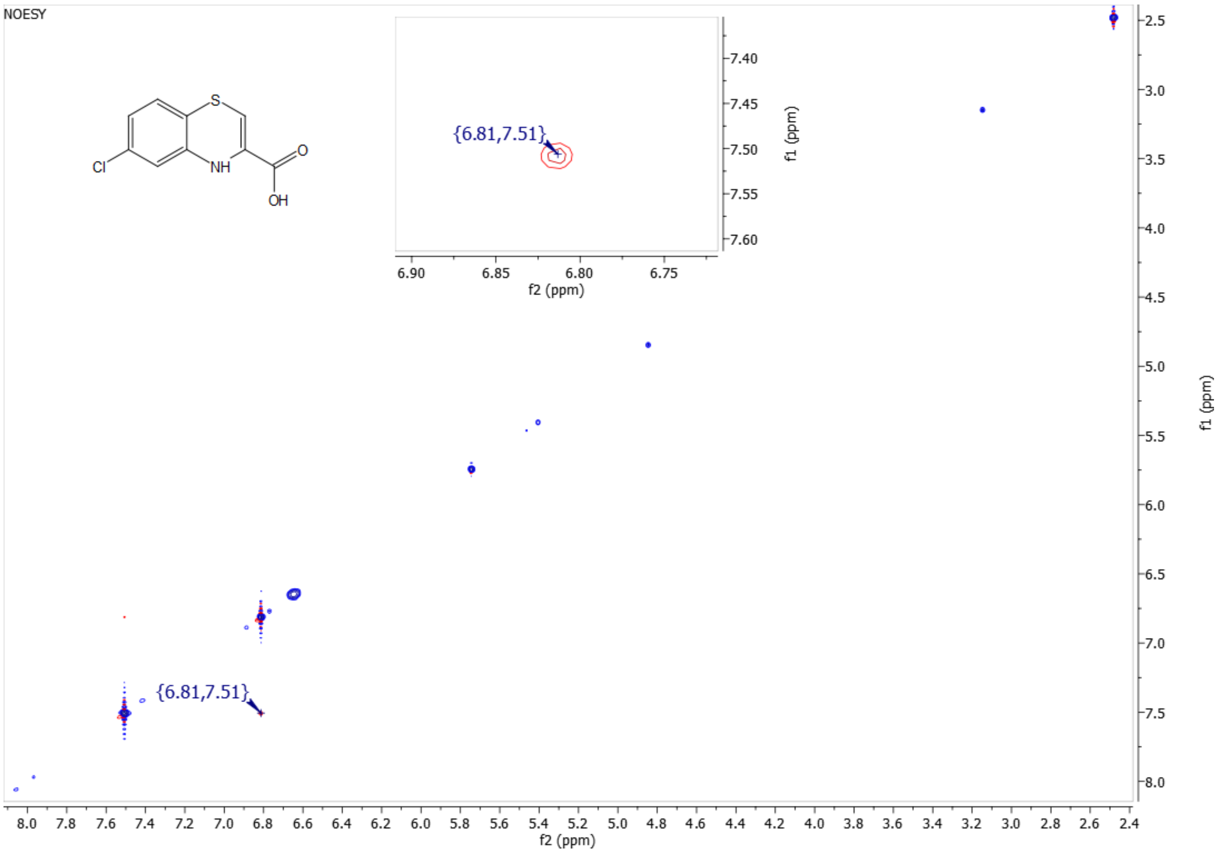

Figure S10. NOESY spectrum of compound **10ba**.

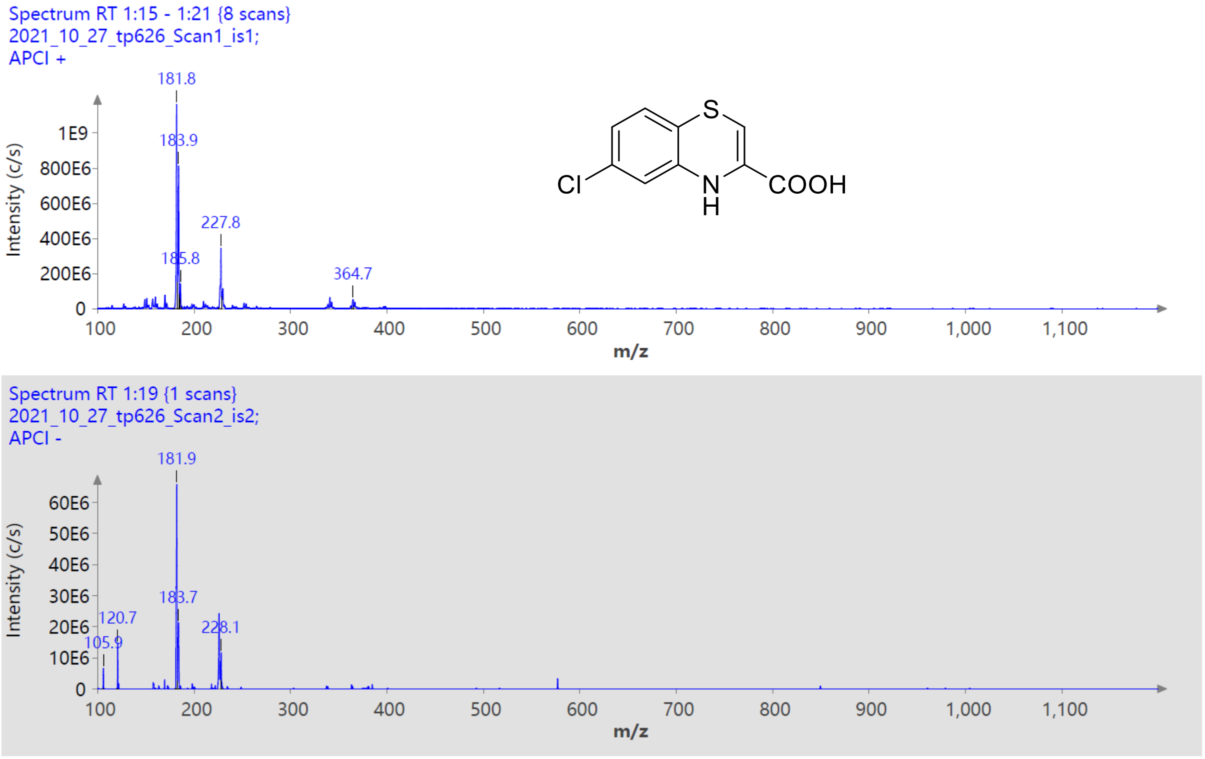

Figure S11. MS spectrum of compound **10ba**.

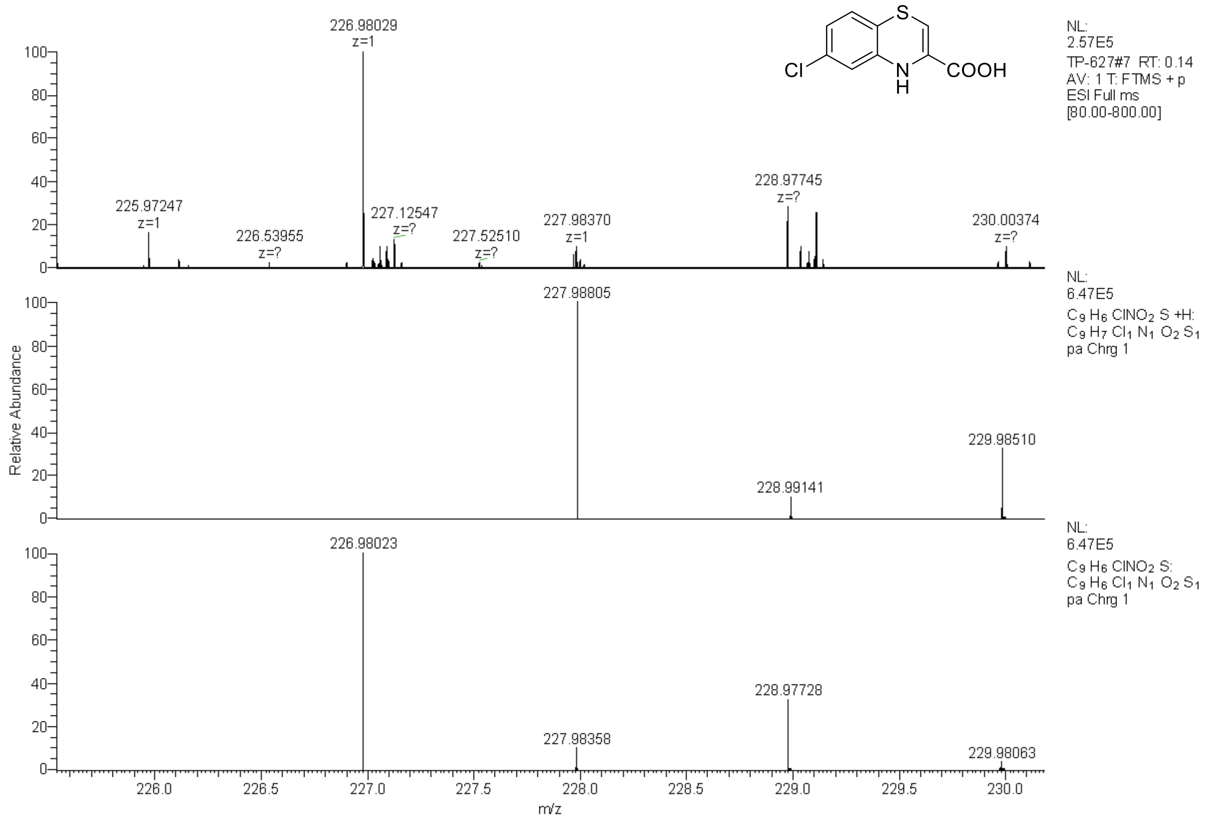

Figure S29. HRMS spectrum of compound **10ba** (HESI).

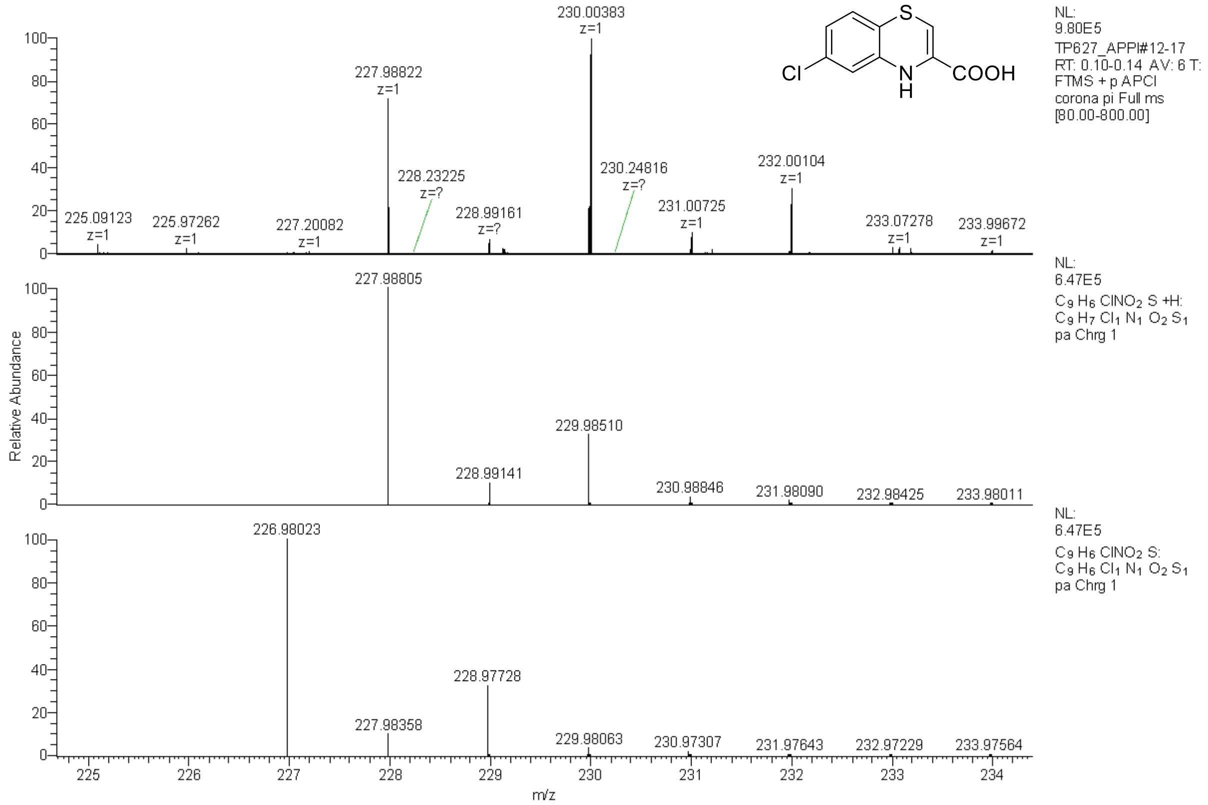

Figure S30. HRMS spectrum of compound **10ba** (APPI).

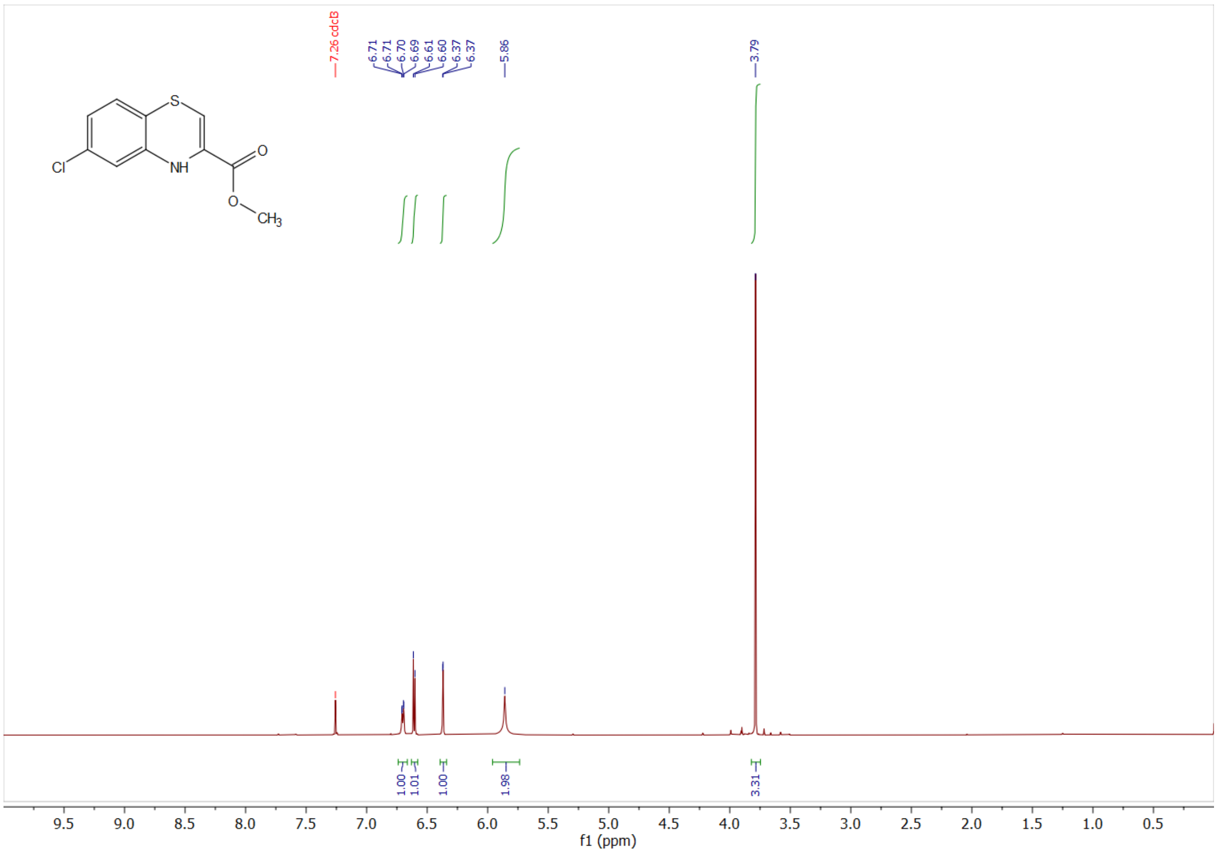

Figure S31. ^{1}H NMR spectrum of compound **10bb**.

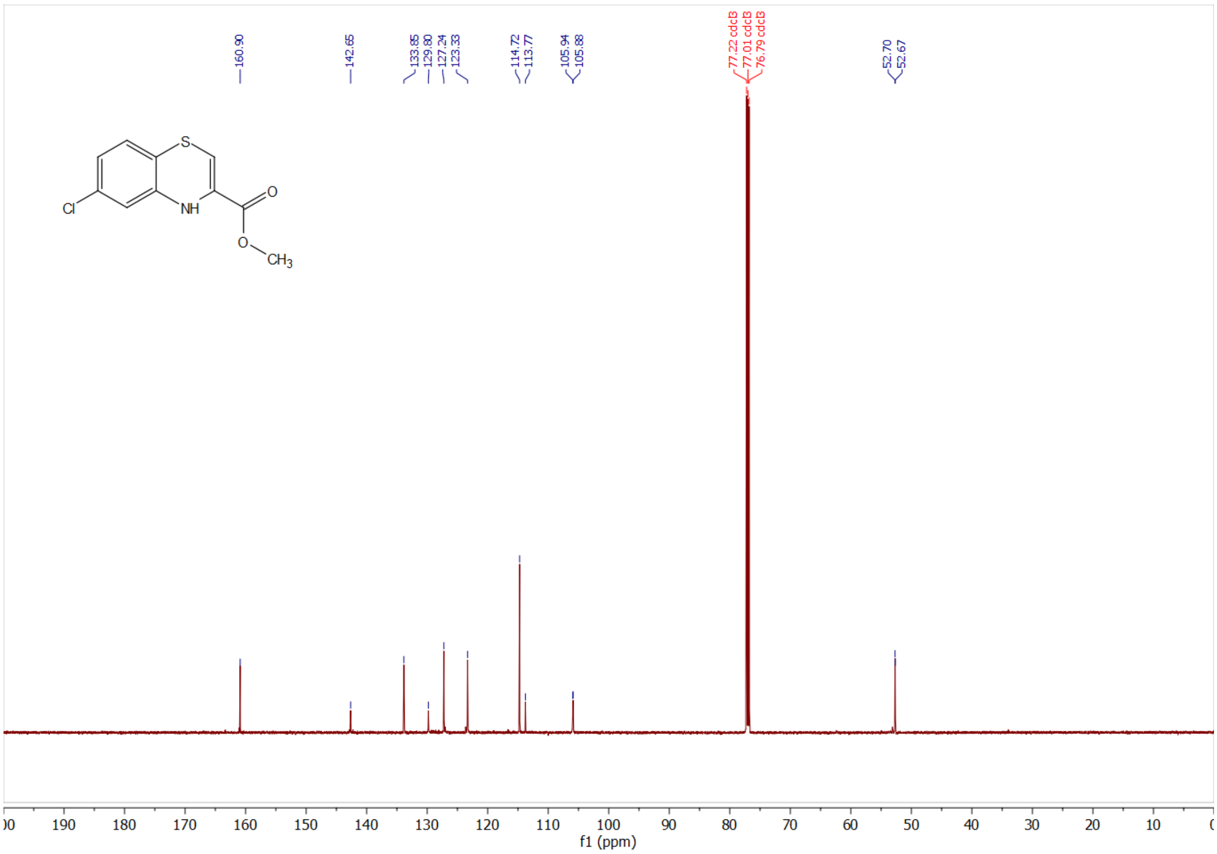

Figure S32. ^{13}C NMR spectrum of compound **10bb**.

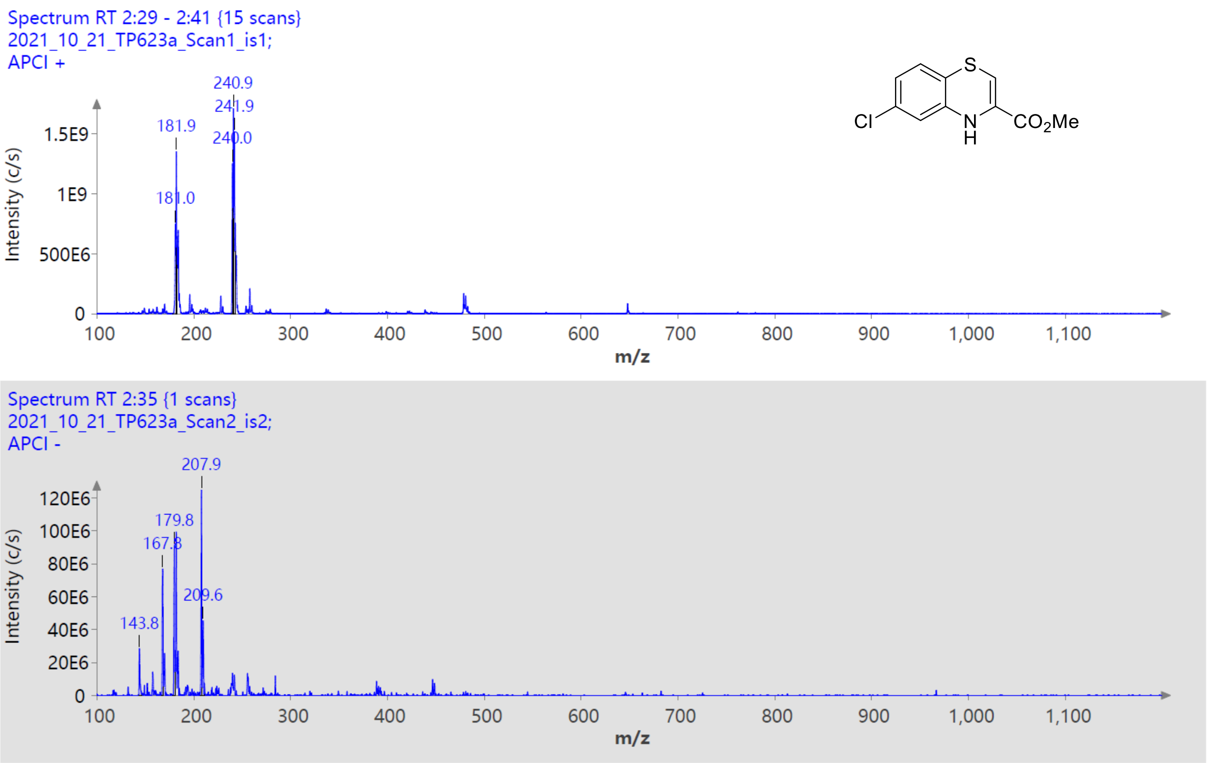

Figure S33. MS spectrum of compound **10bb**.

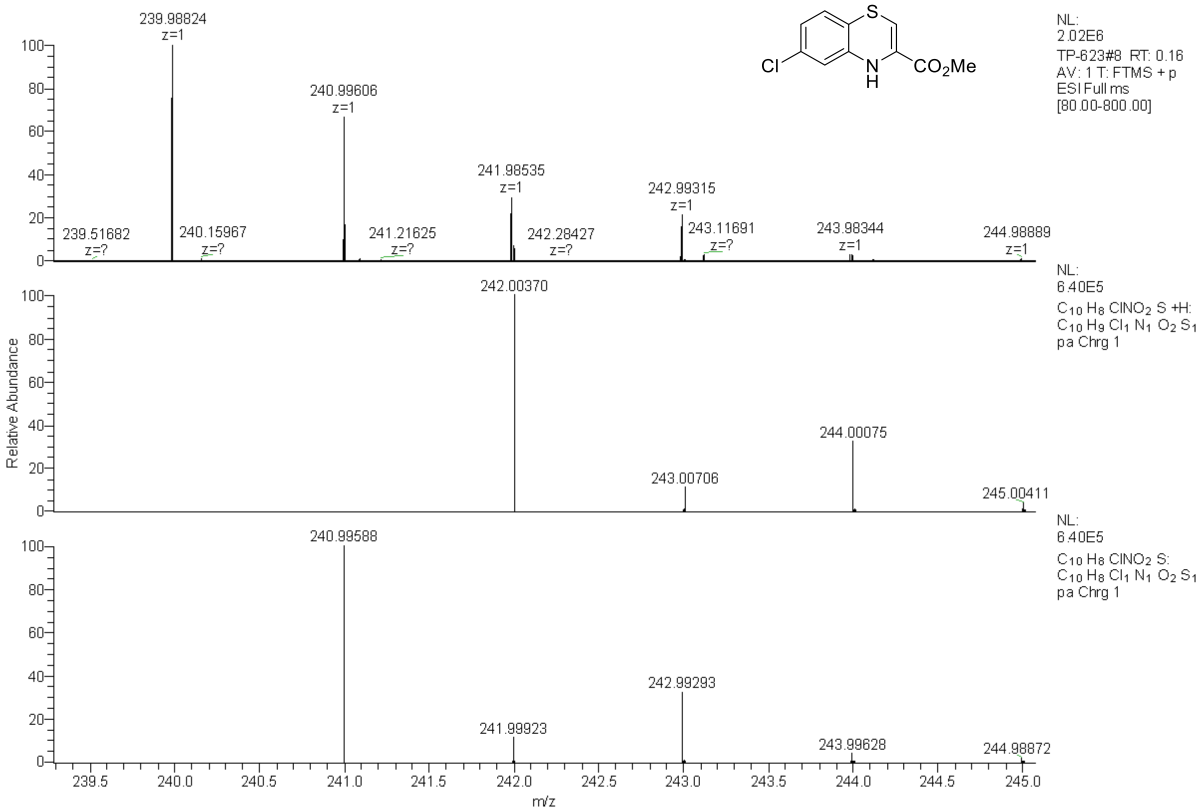

Figure S34. HRMS spectrum of compound **10bb**.

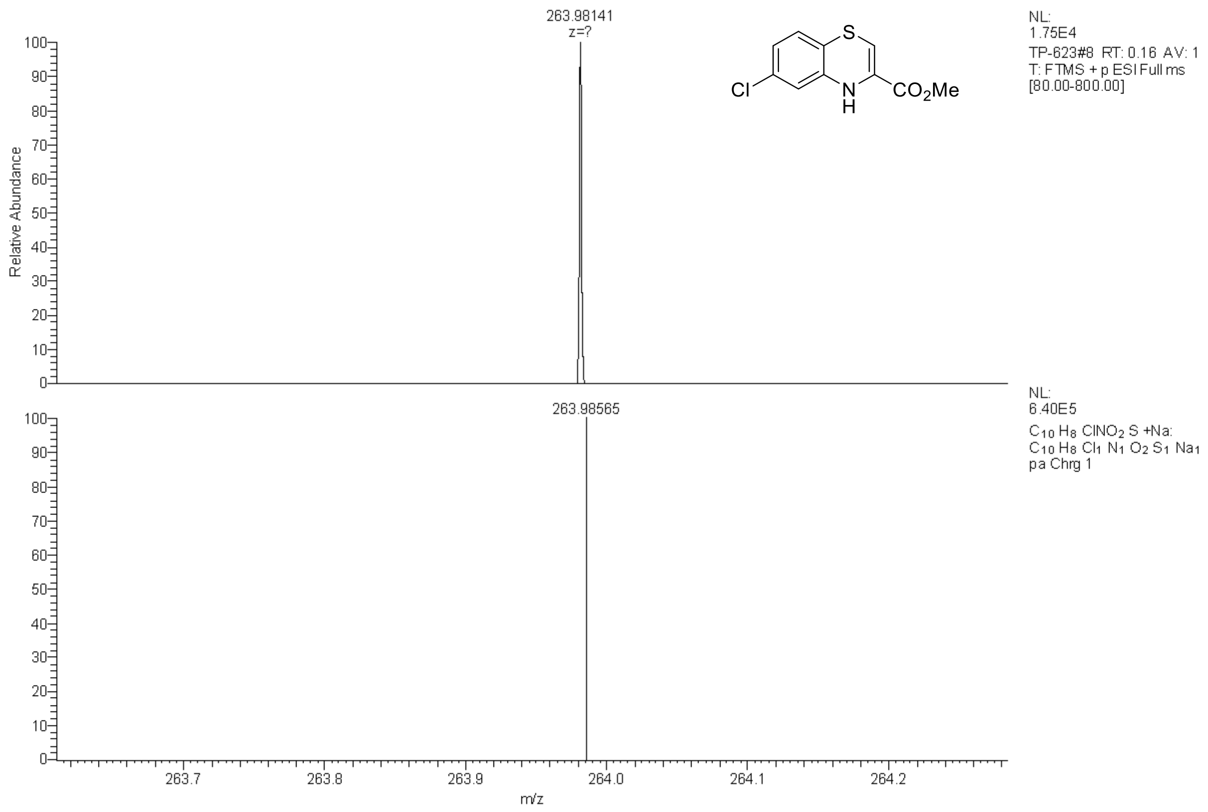

Figure S35. HRMS spectrum of compound **10bb**.

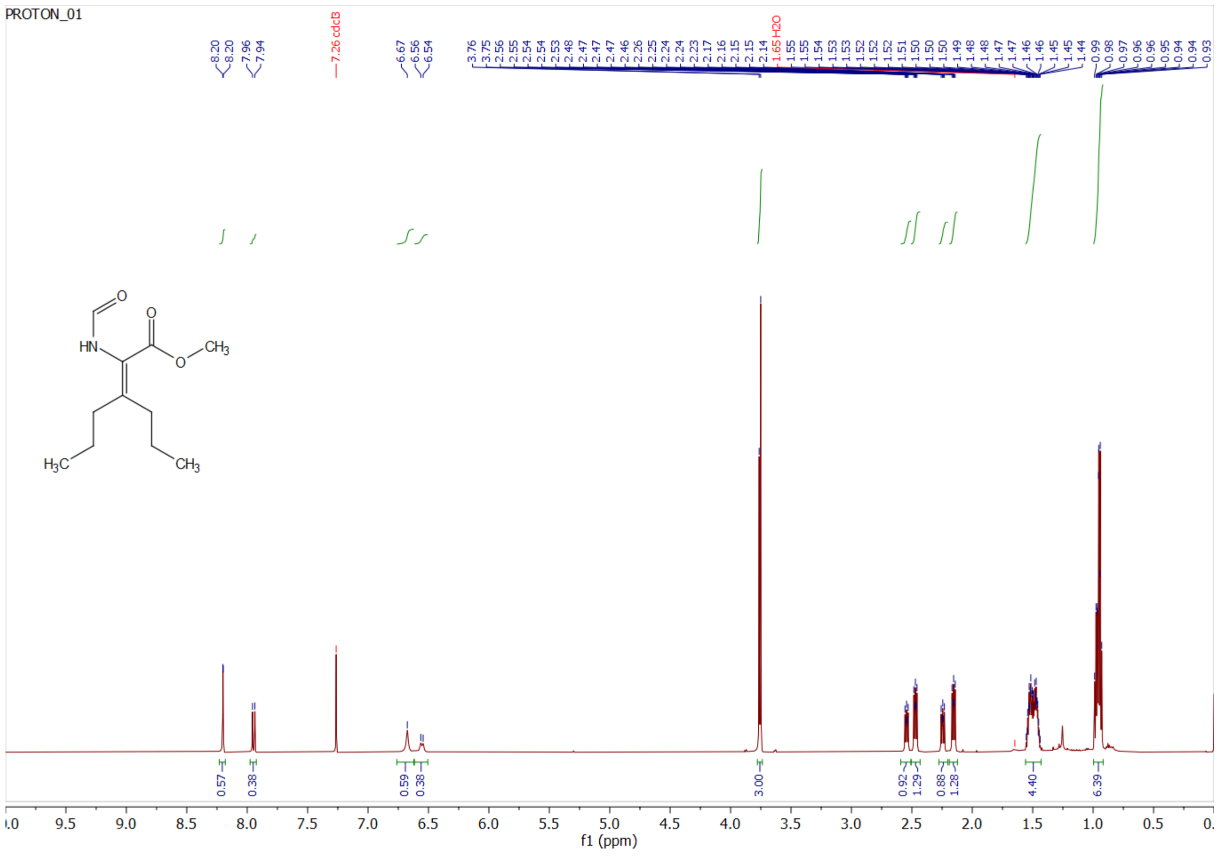

Figure S36. ^{1}H NMR spectrum of compound **14**.

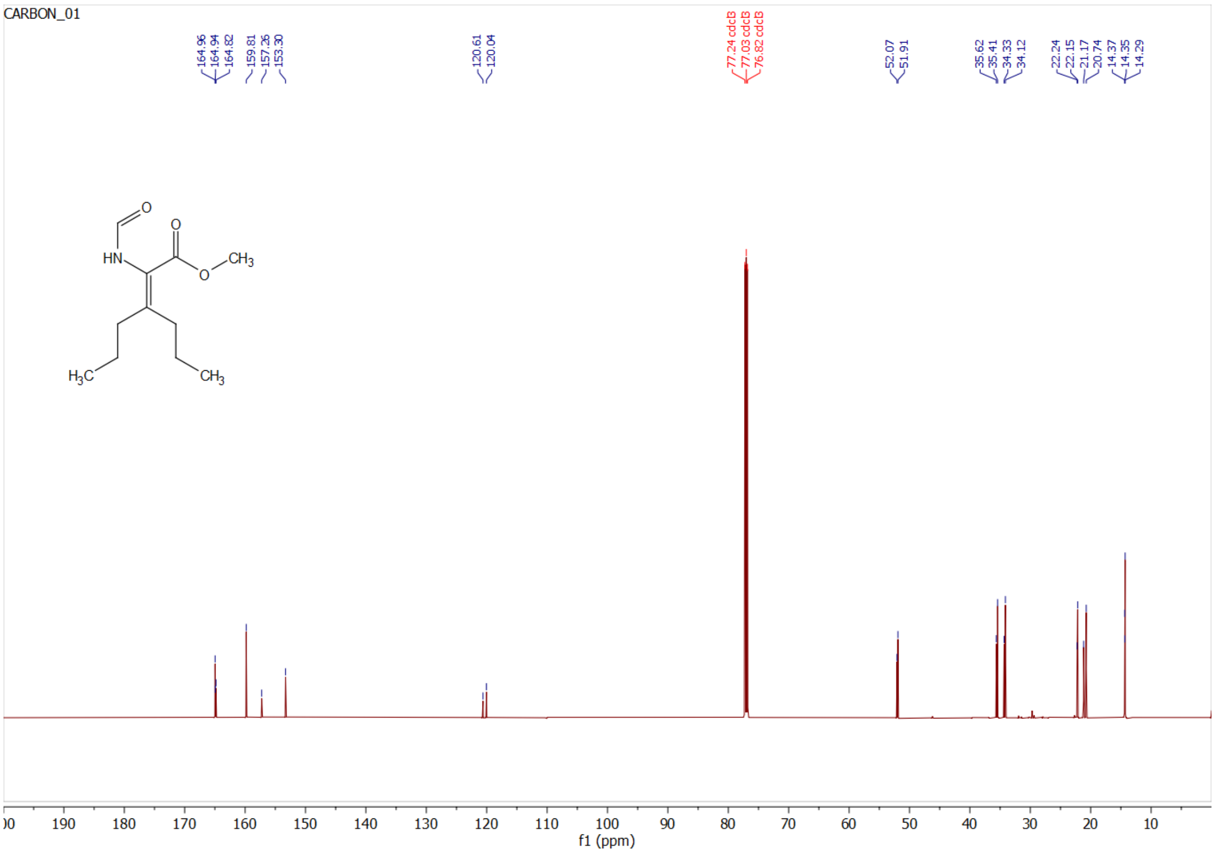

Figure S37. ^{13}C NMR spectrum of compound **14**.

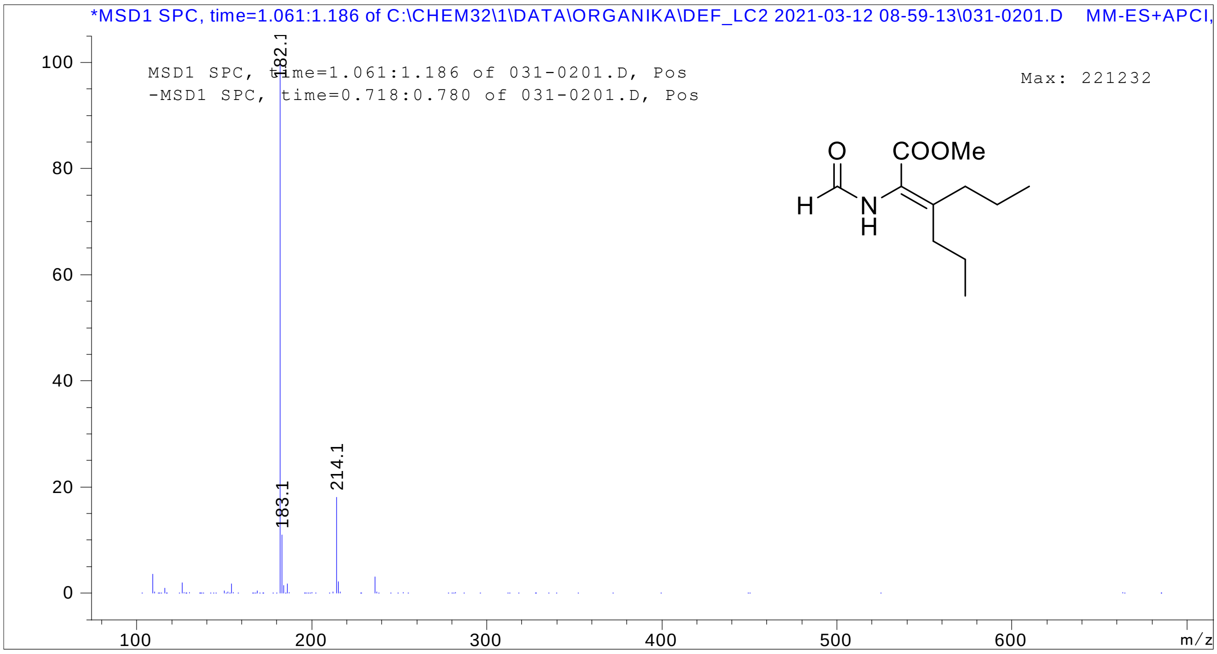

Figure S38. MS spectrum of compound **14** (MM-ES+APCI positive mode).

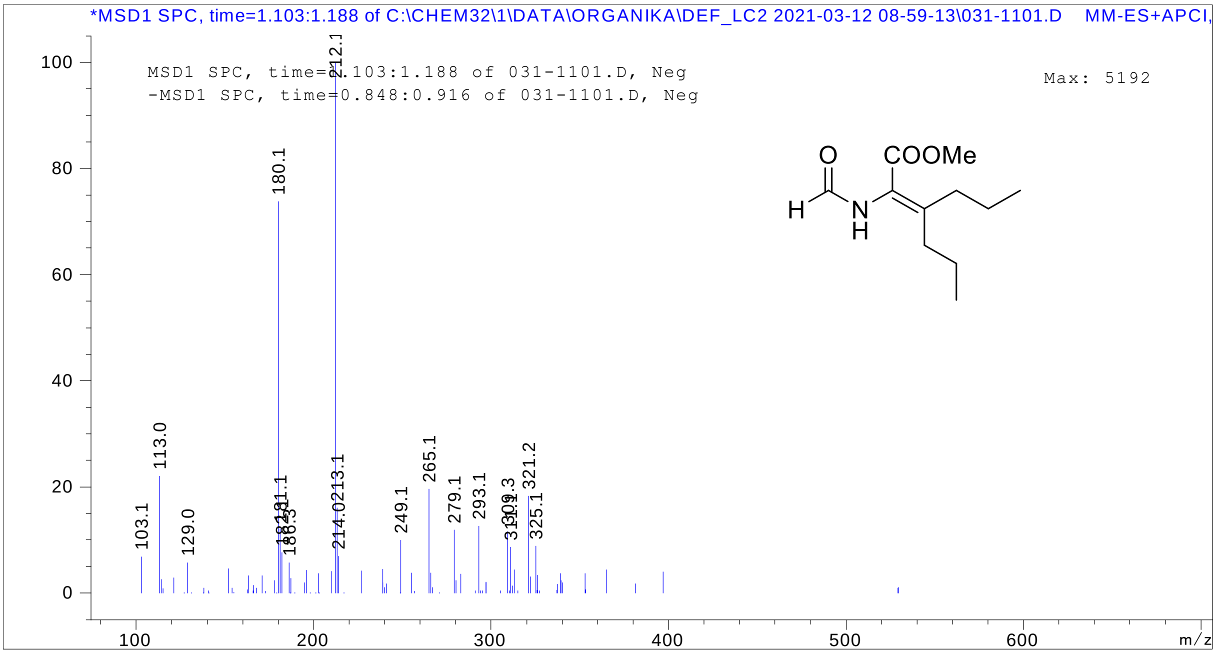

Figure S39. MS spectrum of compound **14** (MM-ES+APCI negative mode).

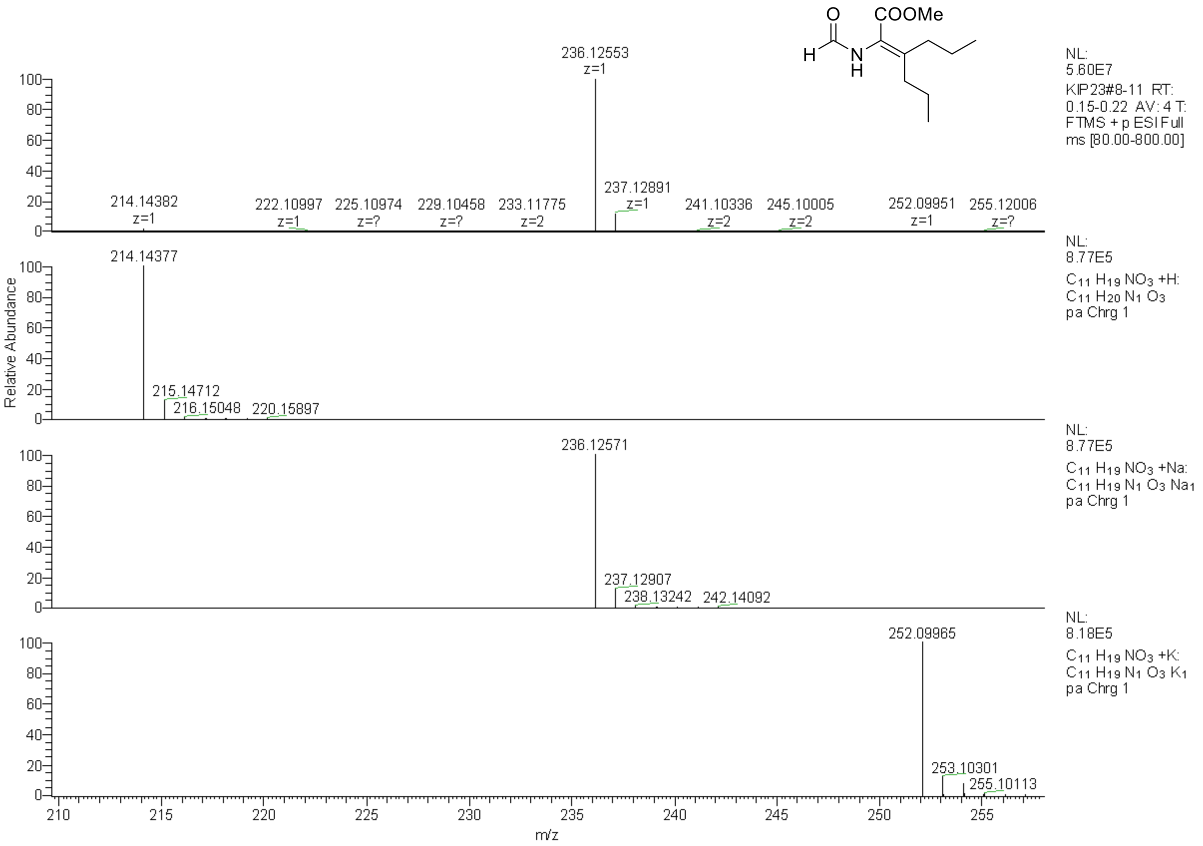

Figure S40. HRMS spectrum of compound **14**.

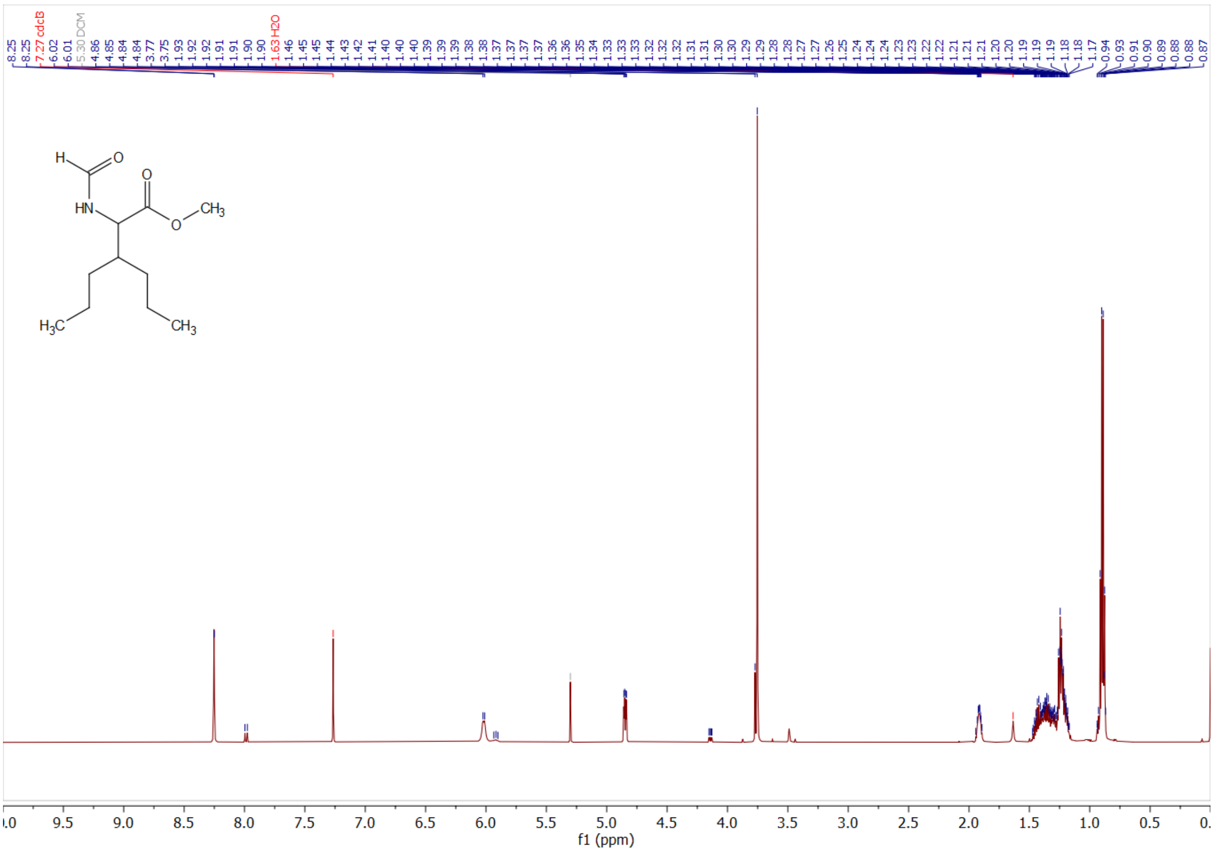

Figure S41. ^{1}H NMR spectrum of compound **15**.

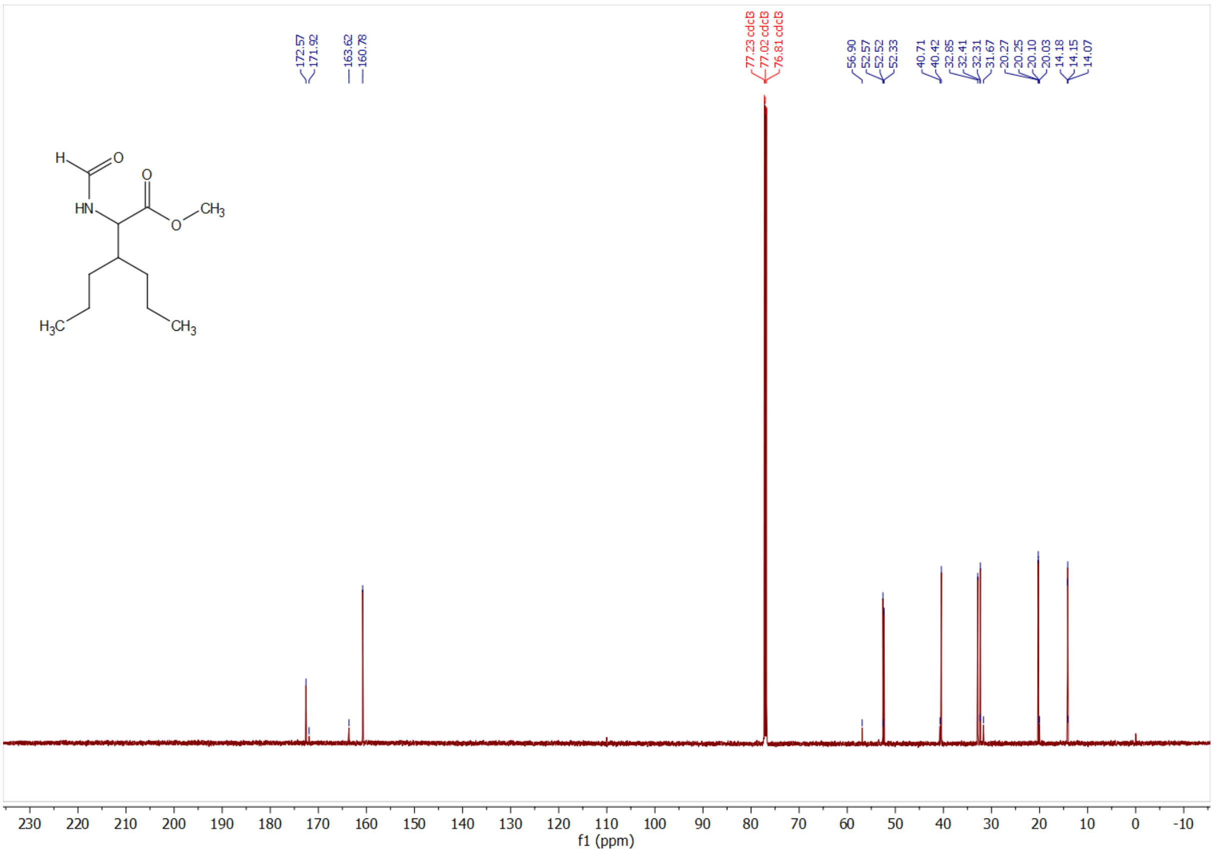

Figure S42. ^{13}C NMR spectrum of compound **15**.

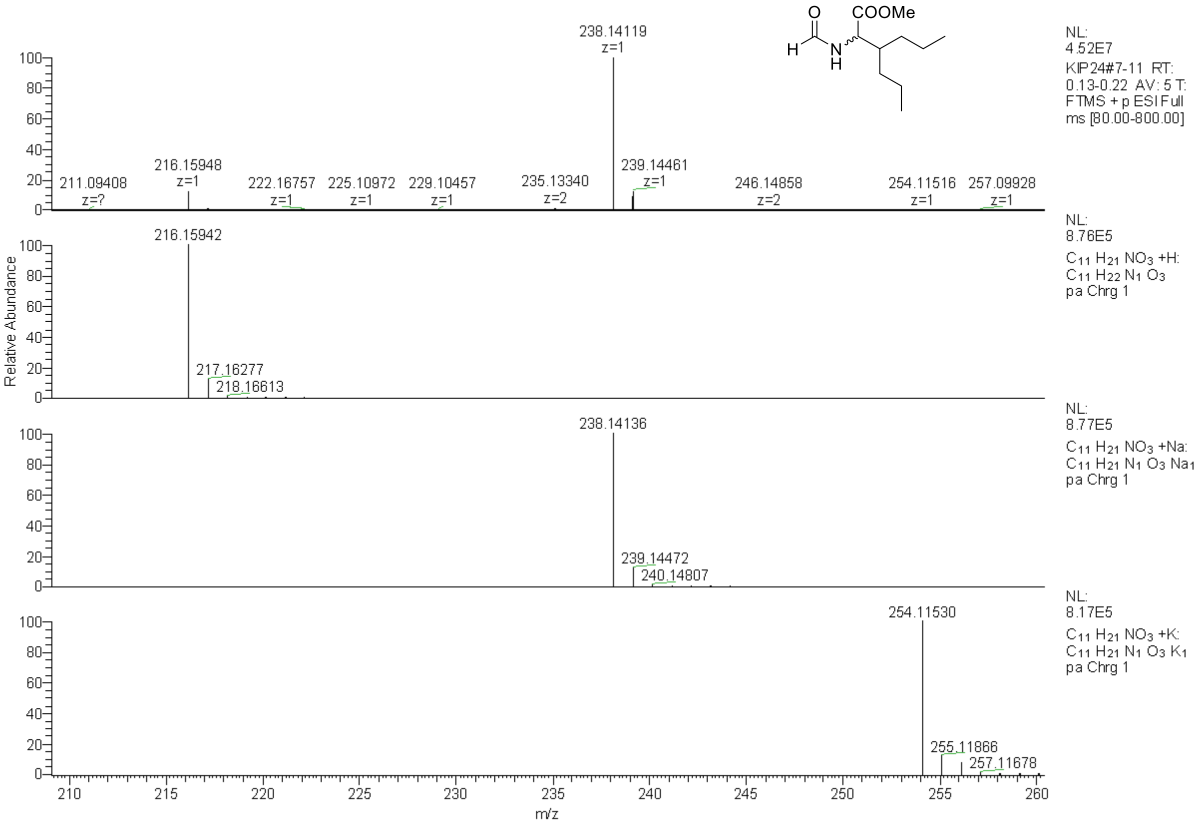

Figure S43. HRMS spectrum of compound **15**.

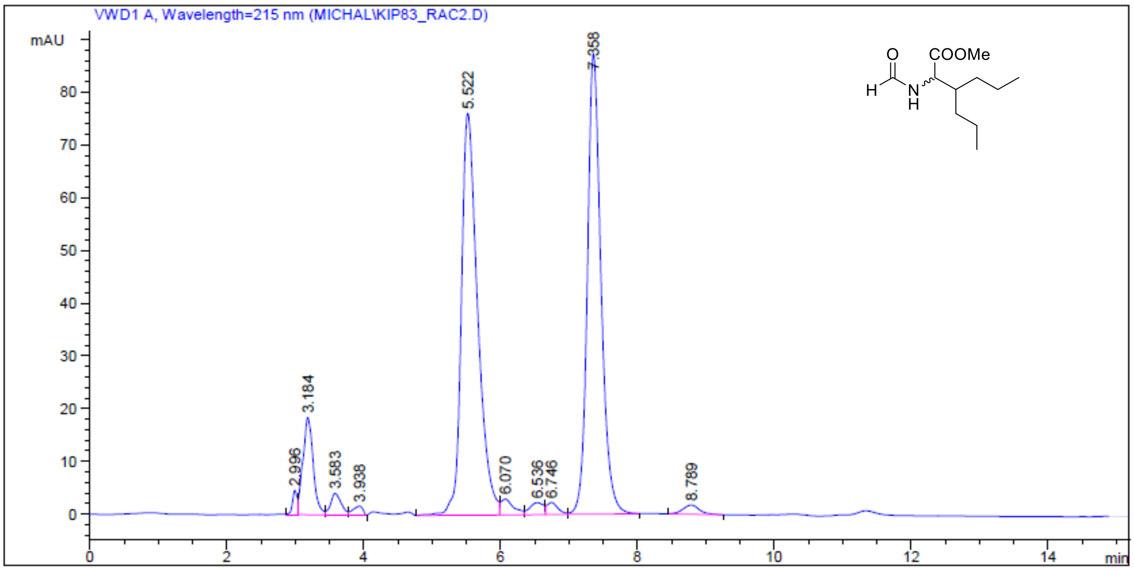

Peak #	RetTime [min]	Type	Width [min]	Area mAU *s	Height [mAU]	Area %
1	2.996	BV	0.0702	21.29345	4.58167	0.7403
2	3.184	VV	0.1562	193.70996	18.41876	6.7347
3	3.583	VV	0.1659	44.07107	4.10908	1.5322
4	3.938	VV	0.1585	17.48224	1.71171	0.6078
5	5.522	VV	0.2489	1264.67139	76.22745	43.9686
6	6.070	VV	0.1867	38.95068	2.99376	1.3542
7	6.536	VV	0.2121	31.59610	2.26762	1.0985
8	6.746	VV	0.1637	25.92975	2.29757	0.9015
9	7.358	VB	0.2113	1209.35095	87.21255	42.0453

Figure S44. HPLC chromatogram of compound **15** (racemic).

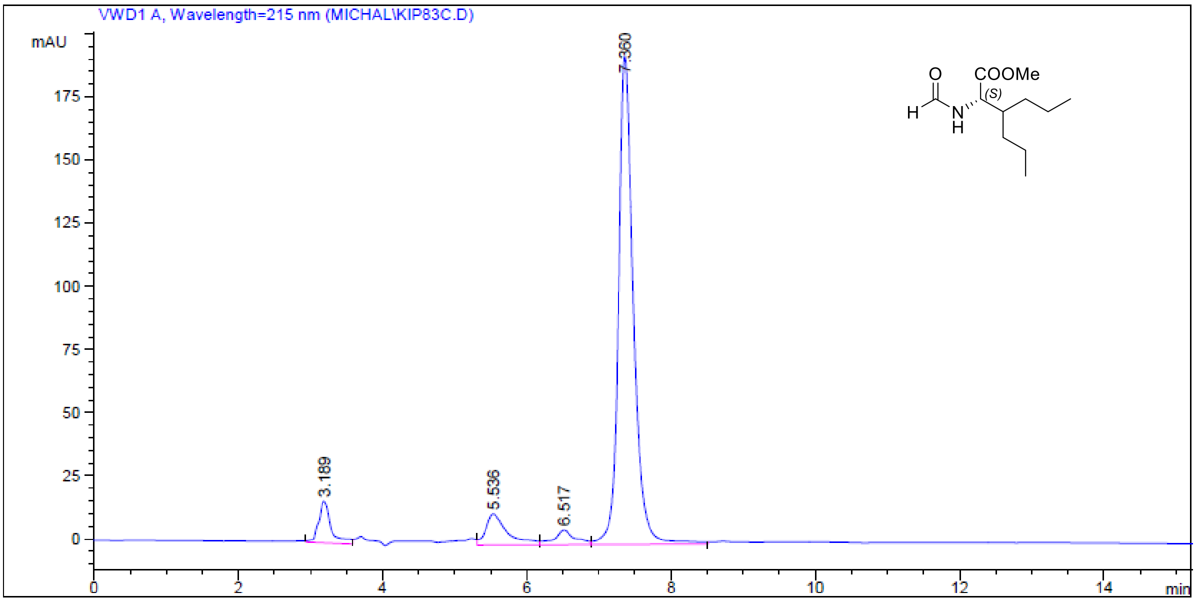

Peak #	RetTime [min]	Type	Width [min]	Area mAU *s	Height [mAU]	Area %
1	3.189	BV	0.1750	197.02626	16.41138	5.9018
2	5.536	VV	0.2996	253.25974	12.24398	7.5862
3	6.517	VV	0.2743	113.09209	5.72157	3.3876
4	7.360	VV	0.2143	2775.02734	193.07364	83.1243

Figure S45. HPLC chromatogram of compound (*S*)-**15** (83% ee).

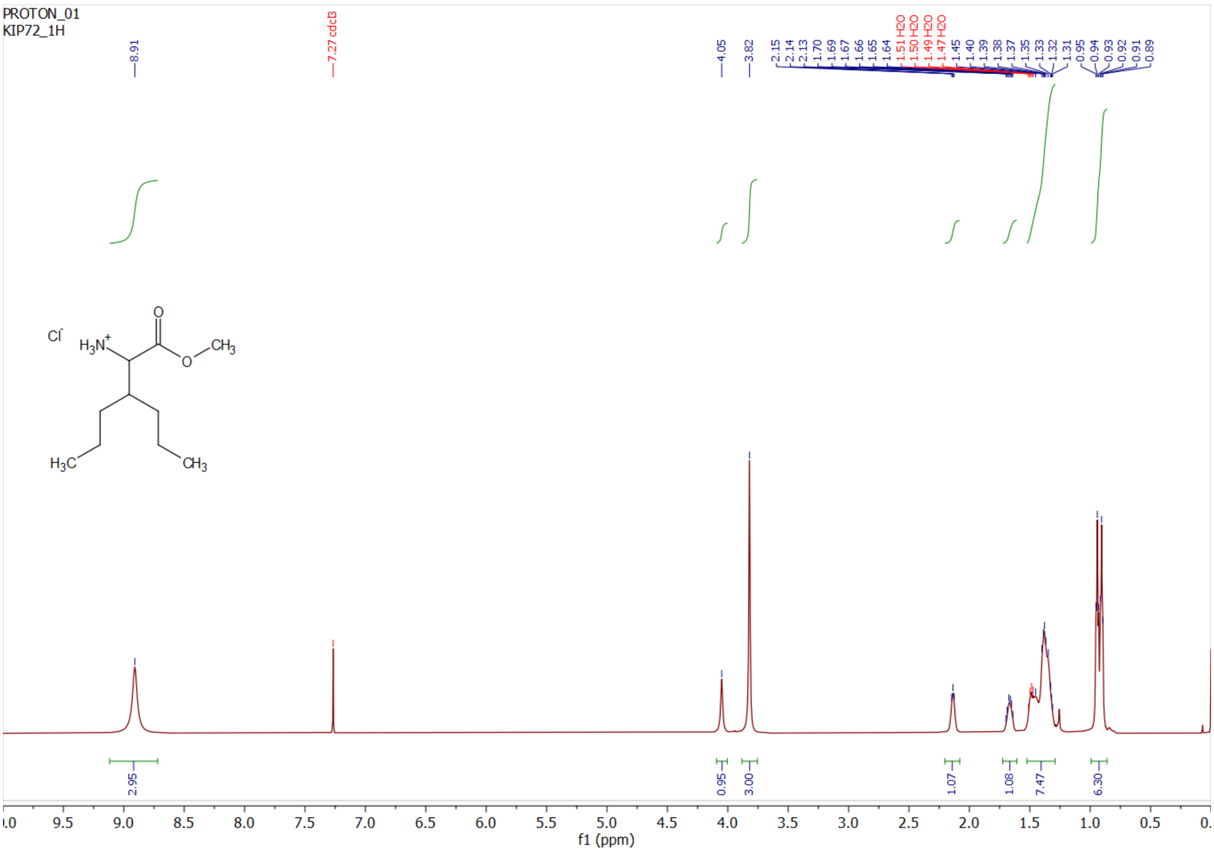

Figure S46. ^{1}H NMR spectrum of compound **16·HCl**.

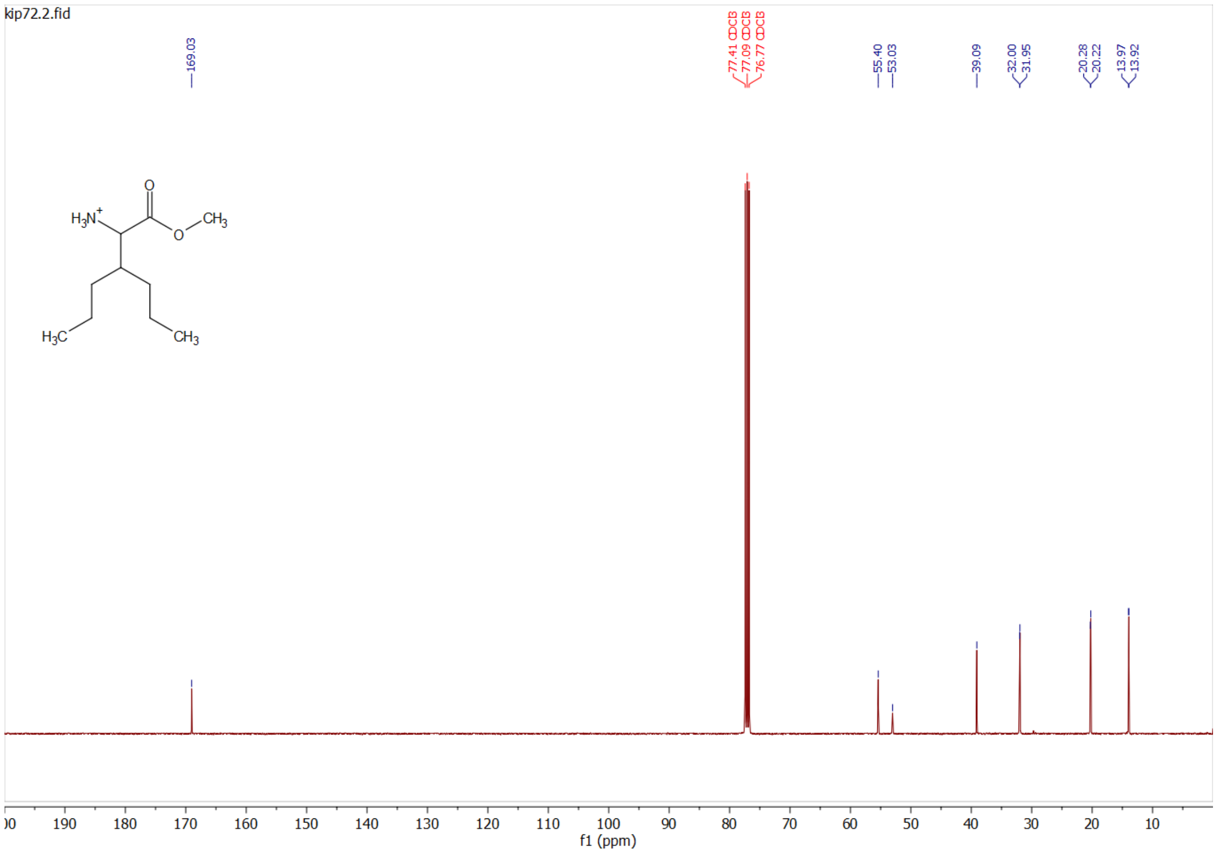

Figure S47. ^{13}C NMR spectrum of compound **16·HCl**.

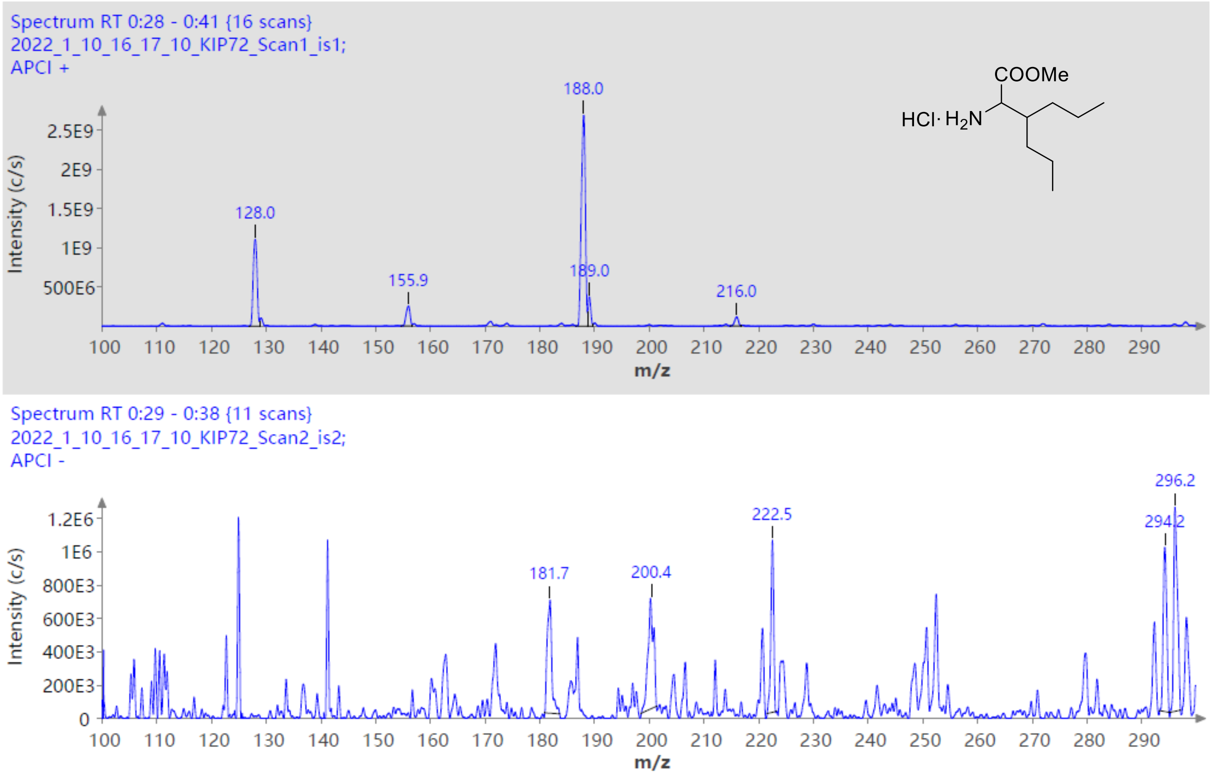

Figure S48. MS spectrum of compound **16·HCl**.

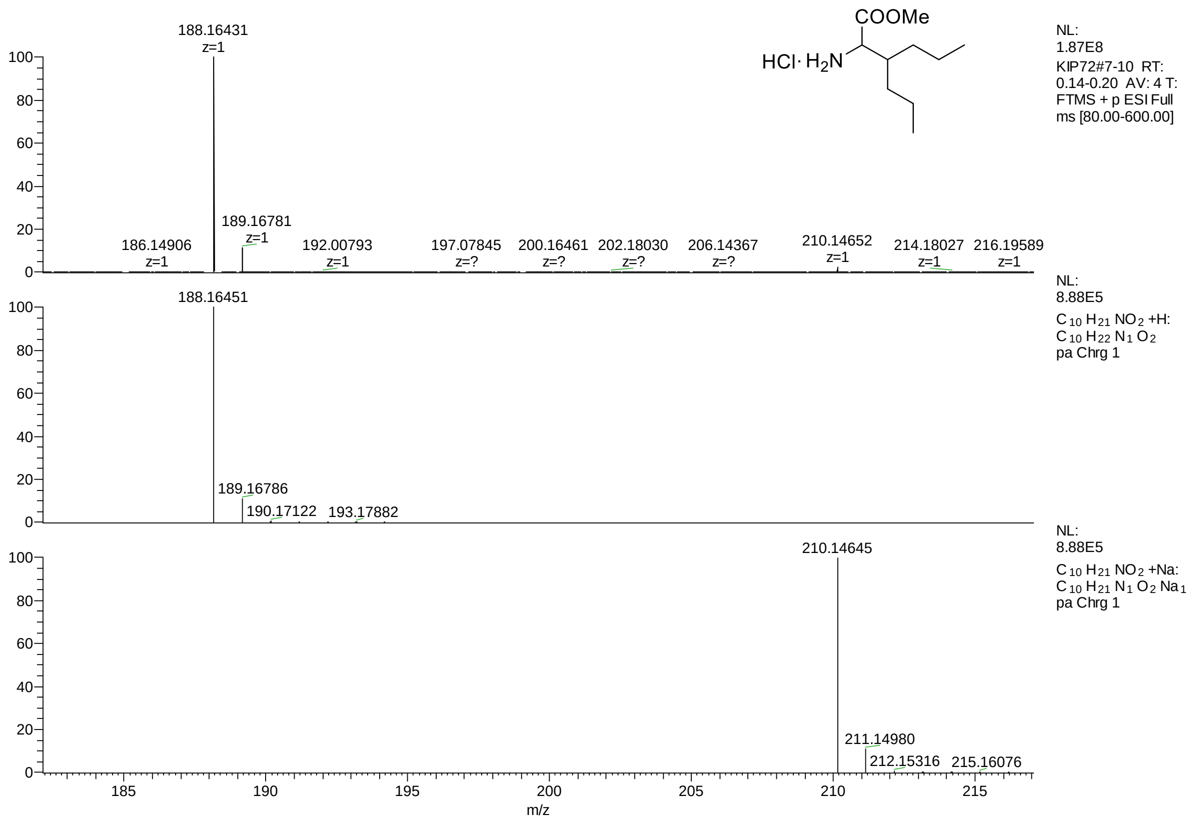

Figure S49. HRMS spectrum of compound **16·HCl**.

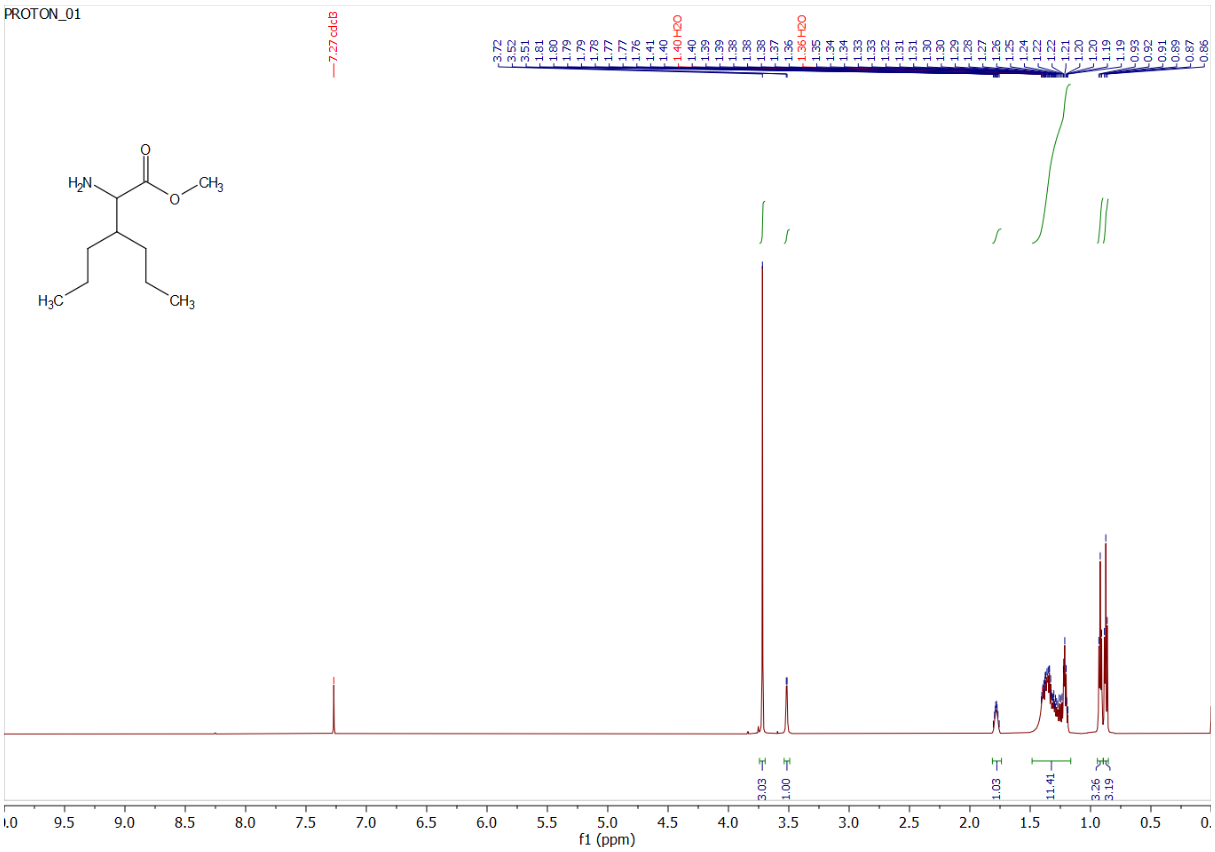

Figure S50. ^{1}H NMR spectrum of compound **16**.

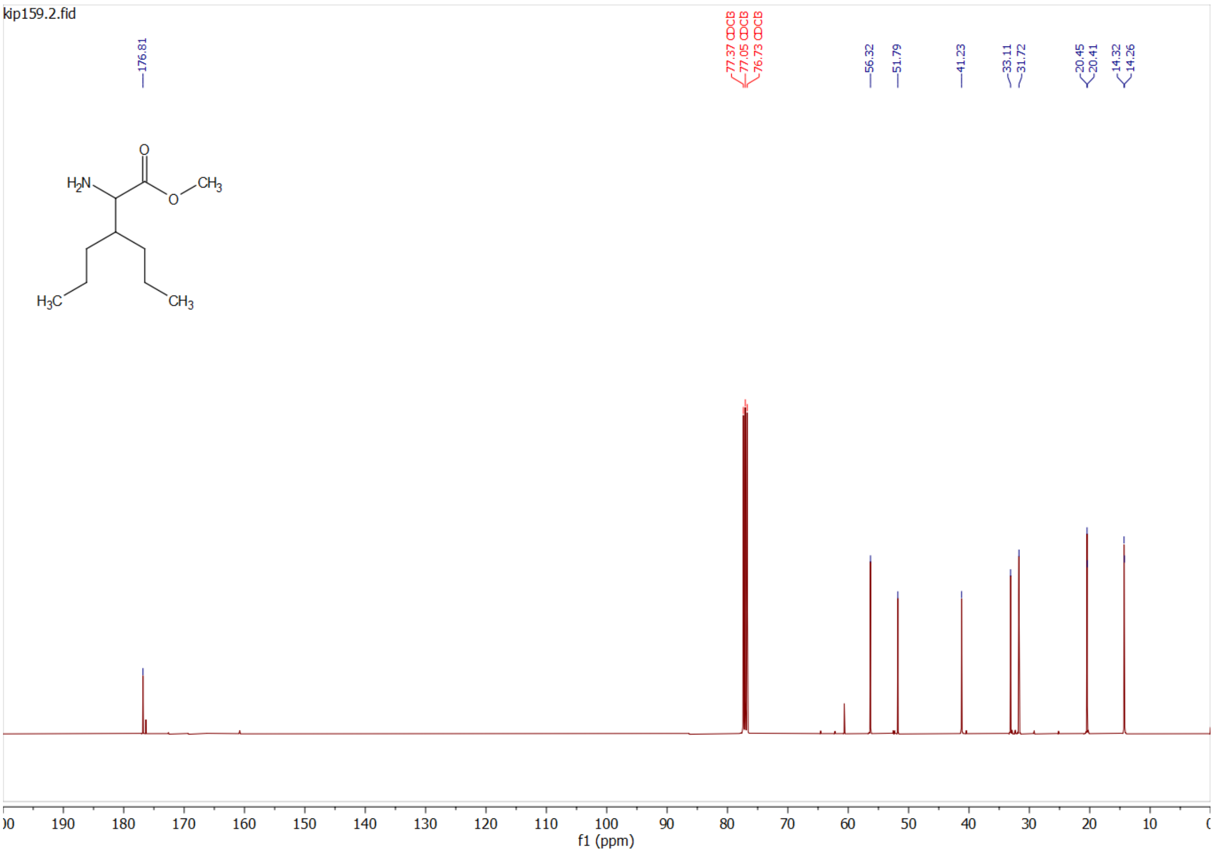

Figure S51. ^{13}C NMR spectrum of compound **16**.

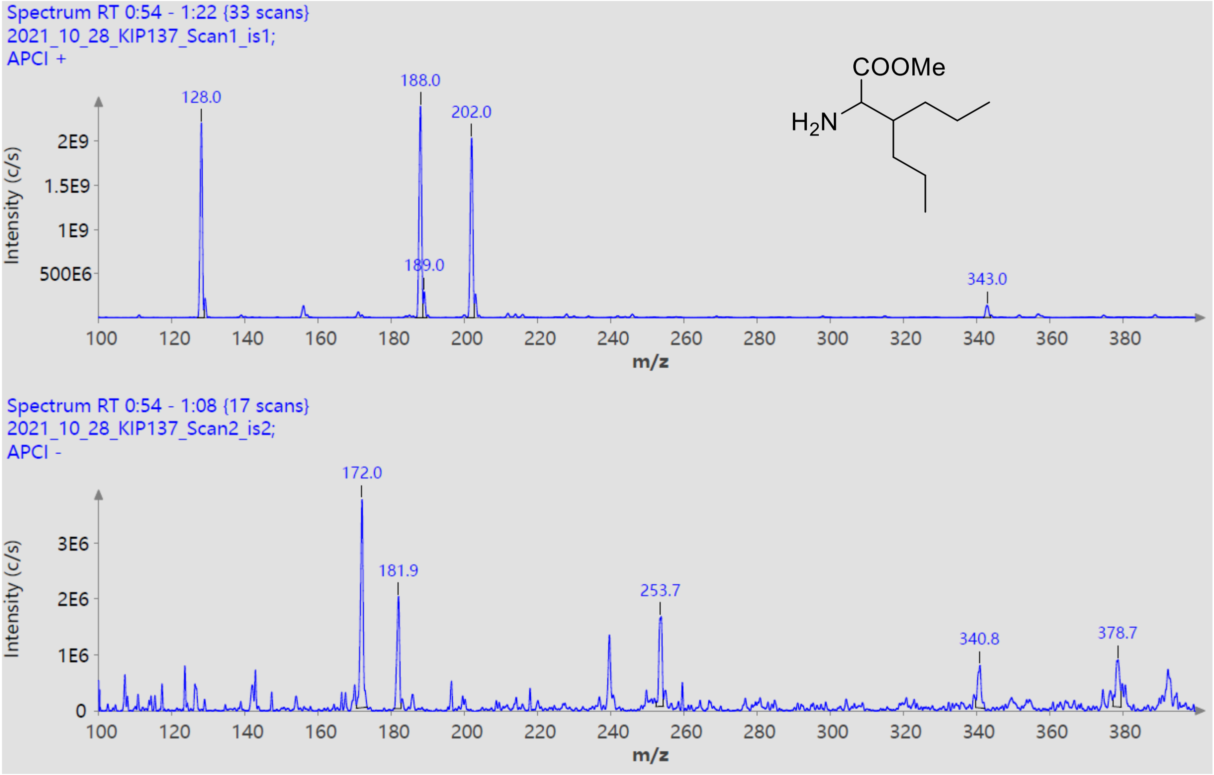

Figure S52. MS spectrum of compound **16**.

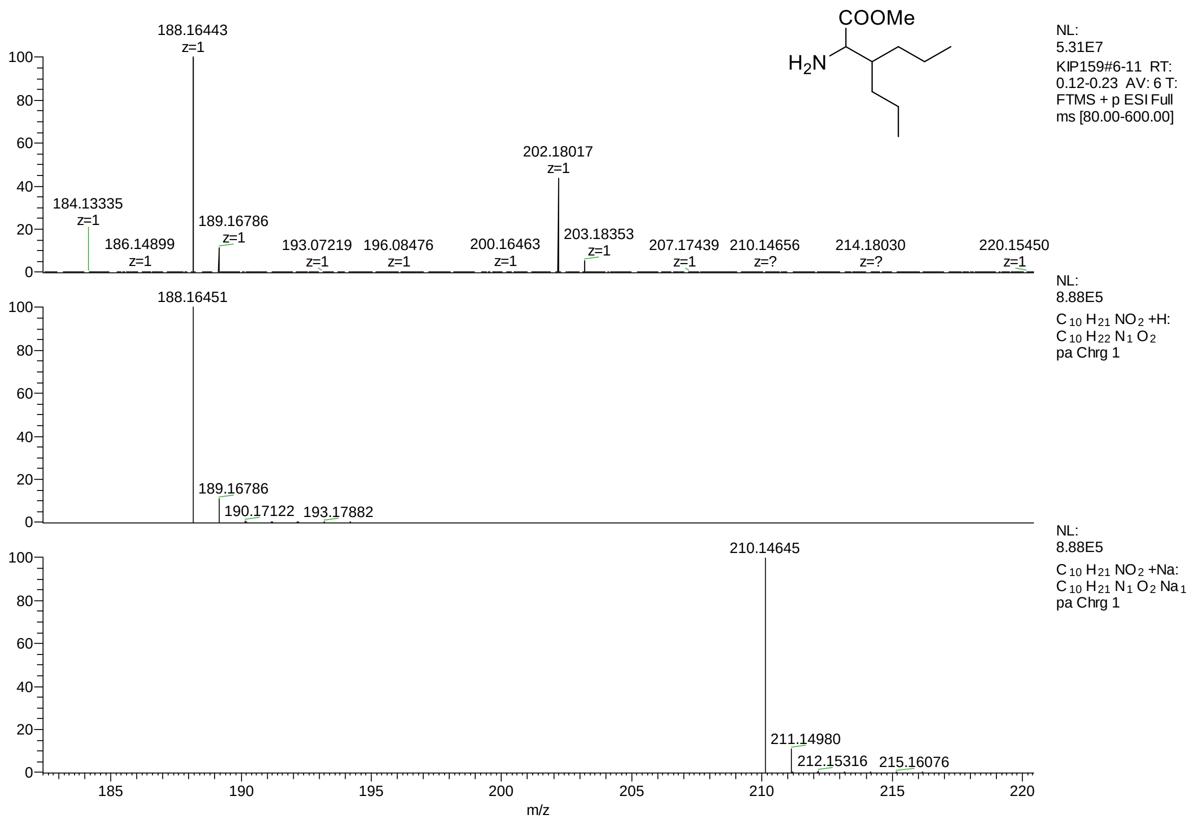

Figure S53. HRMS spectrum of compound **16**.

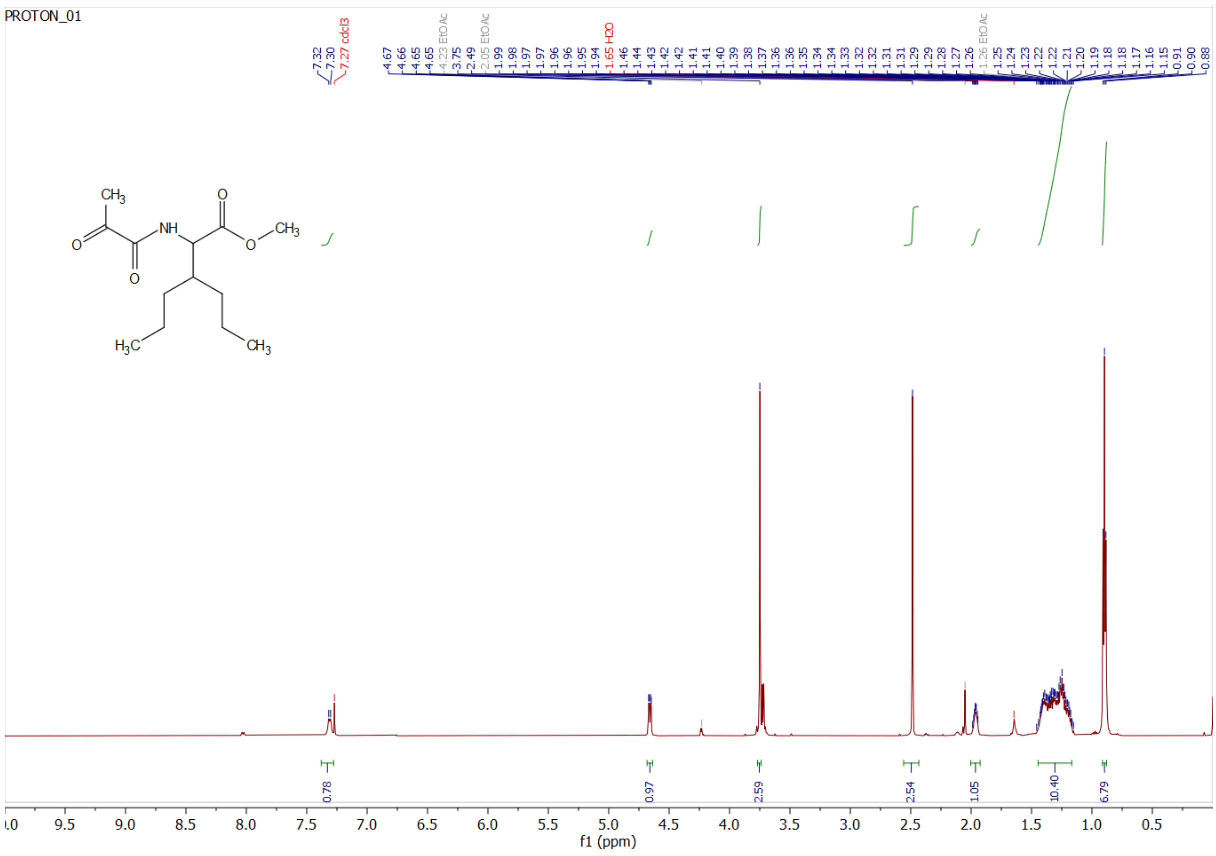

Figure S54. ^{1}H NMR spectrum of compound **17a**.

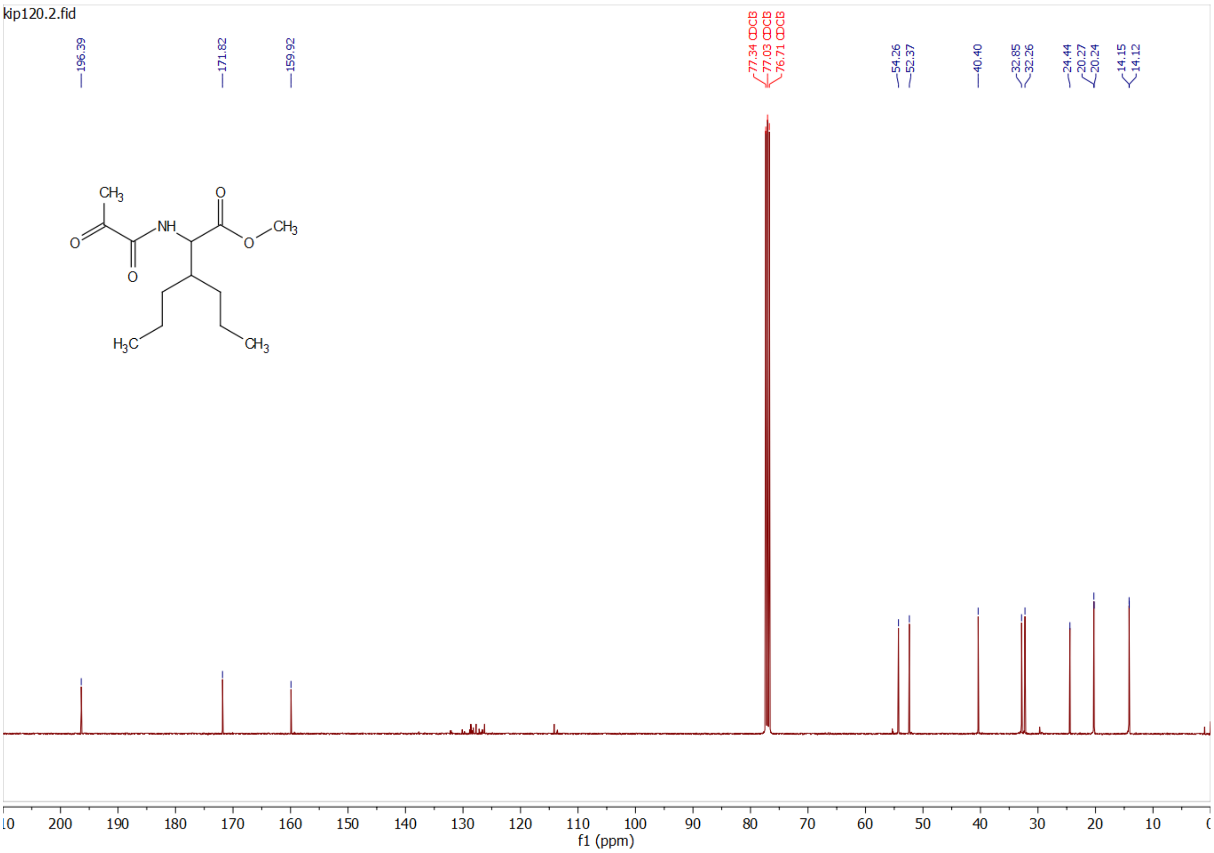

Figure S55. ^{13}C NMR spectrum of compound **17a**.

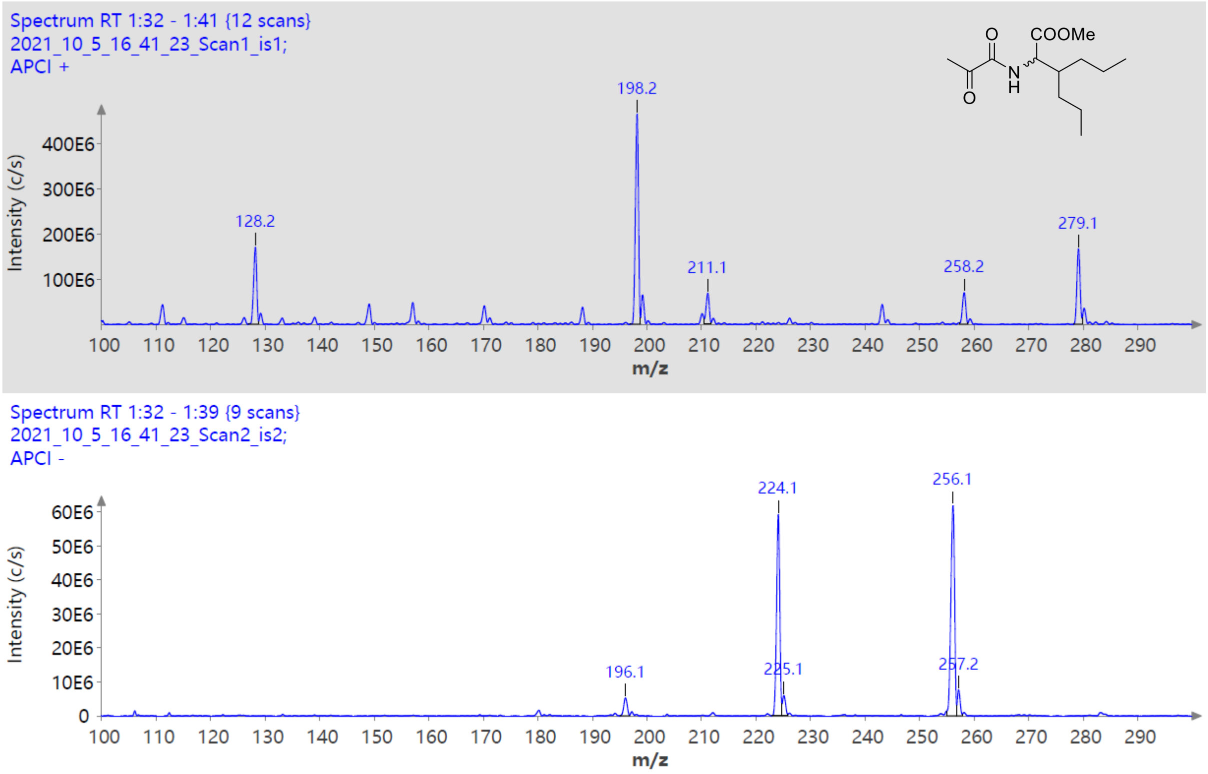

Figure S56. MS spectrum of compound **17a**.

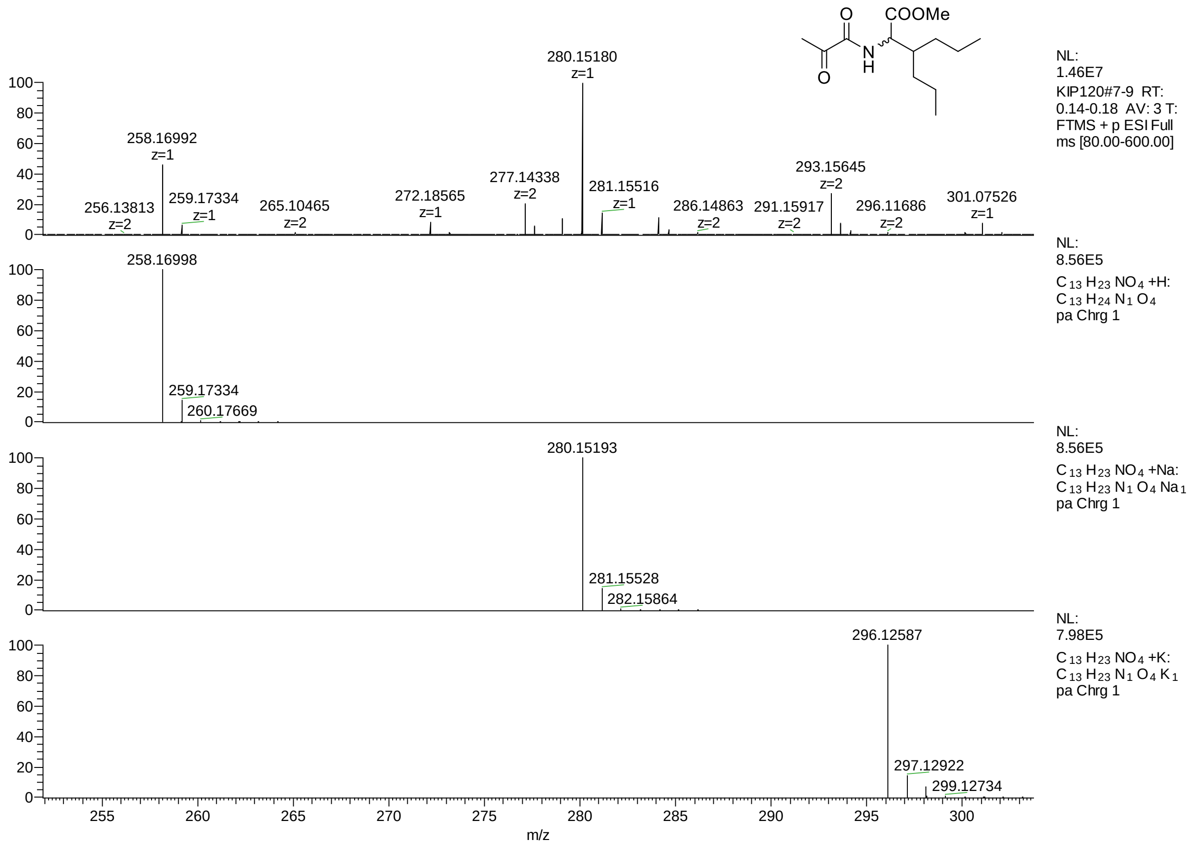

Figure S57. HRMS spectrum of compound **17a**.

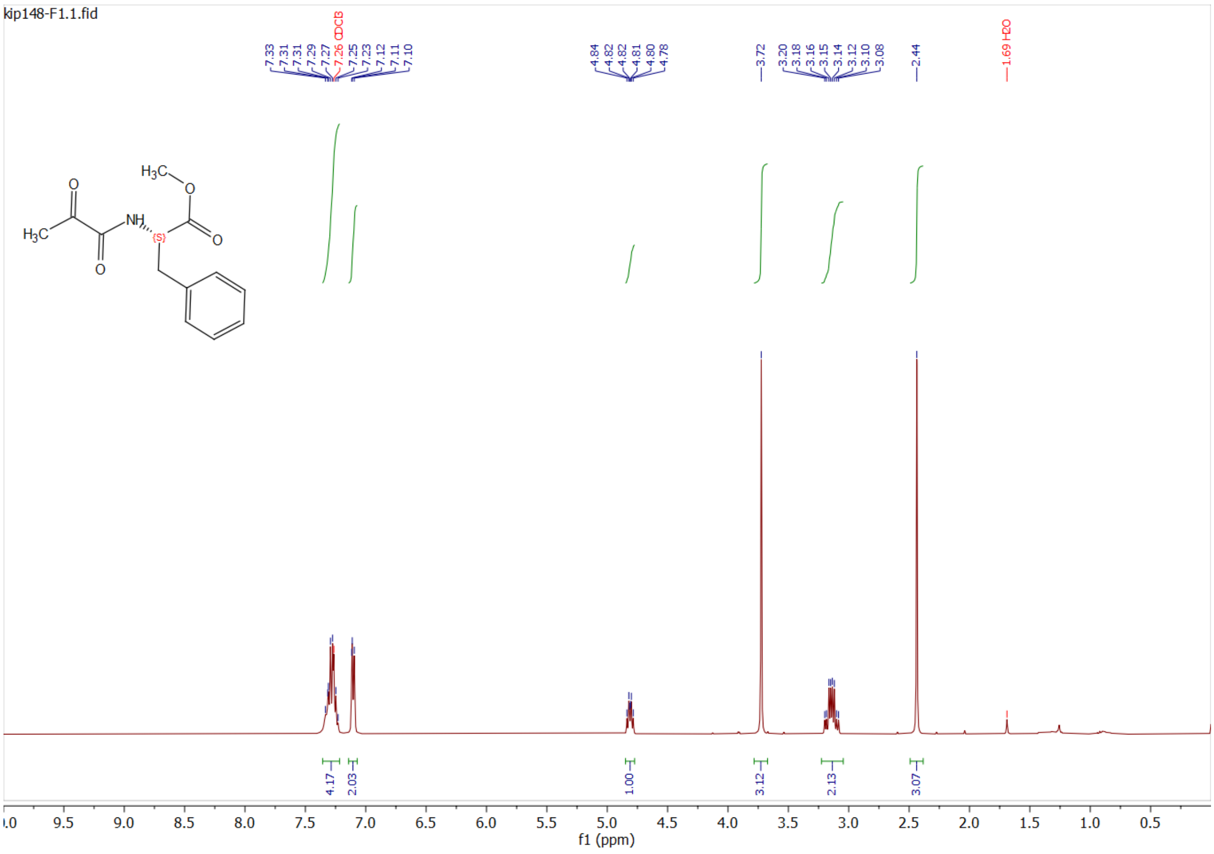

Figure S58. ^{1}H NMR spectrum of compound **17b**.

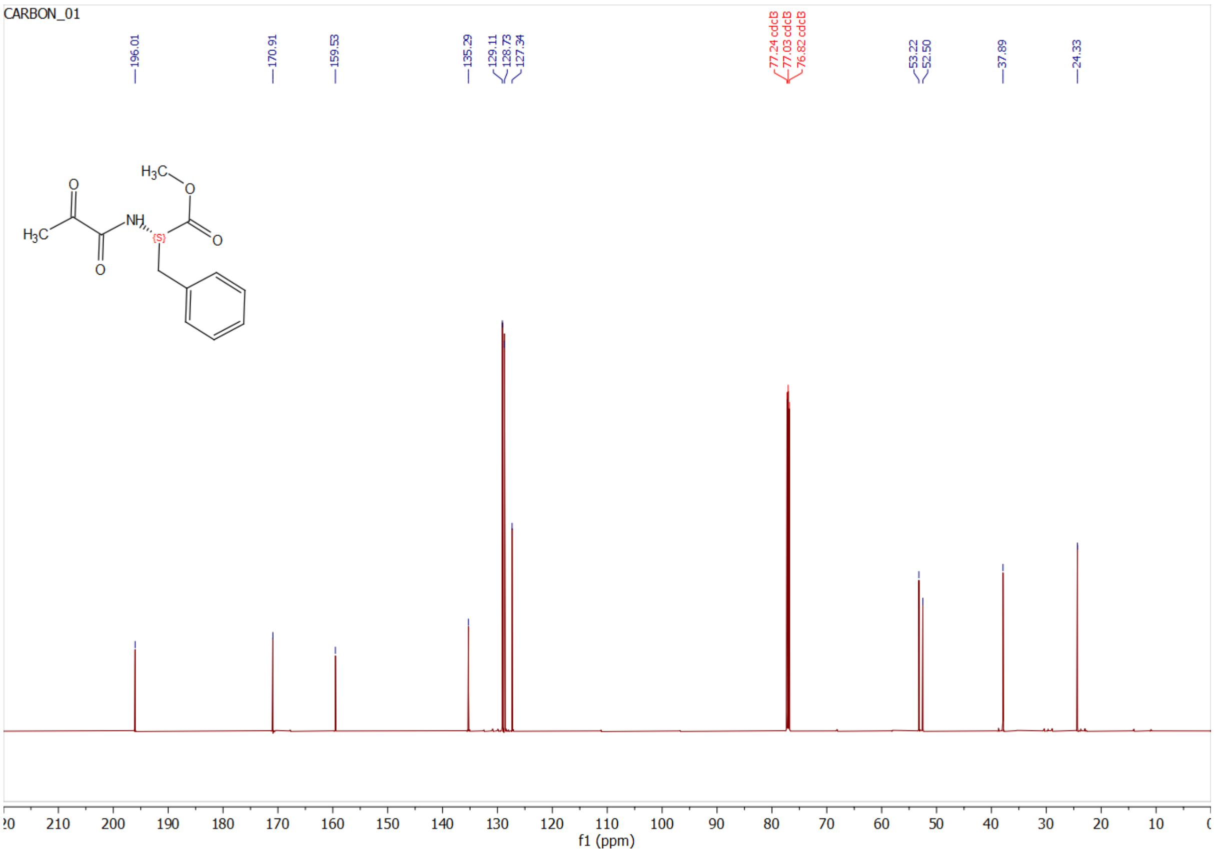

Figure S59. ^{13}C NMR spectrum of compound **17b**.

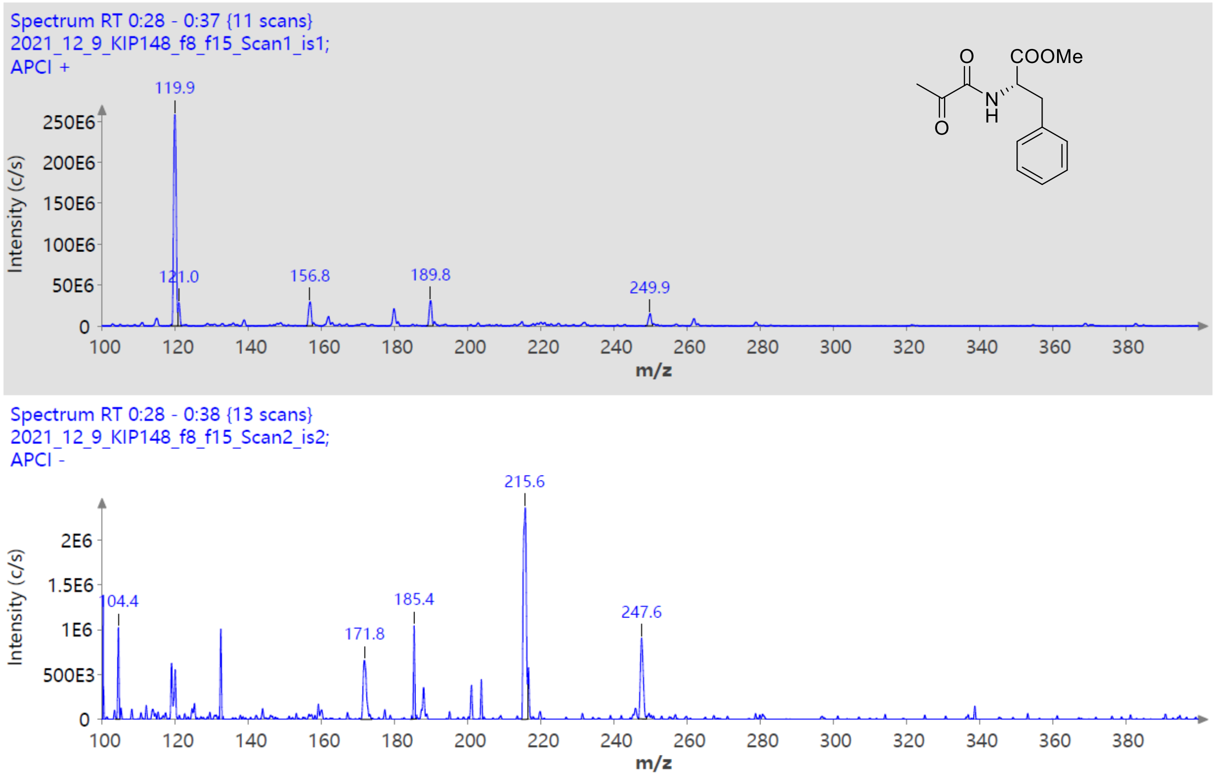

Figure S60. MS spectrum of compound **17b**.

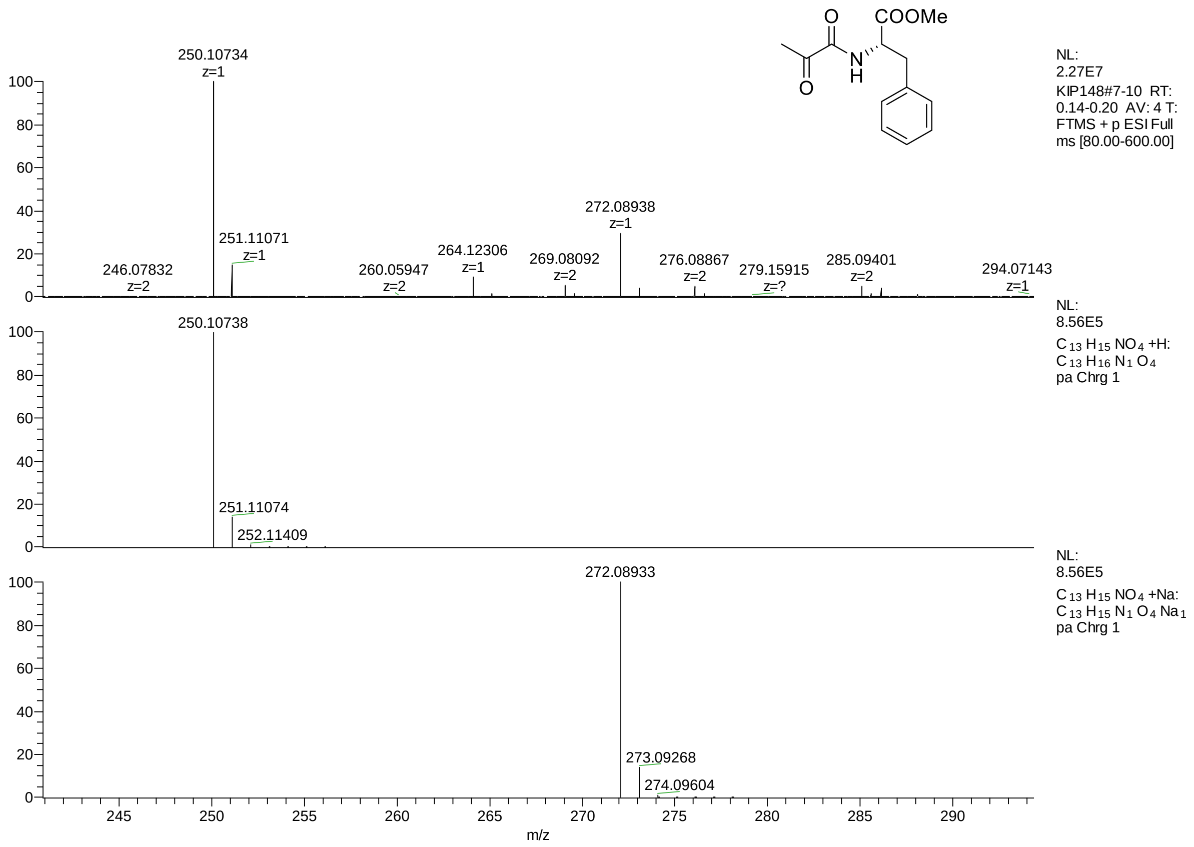

Figure S61. HRMS spectrum of compound **17b**.

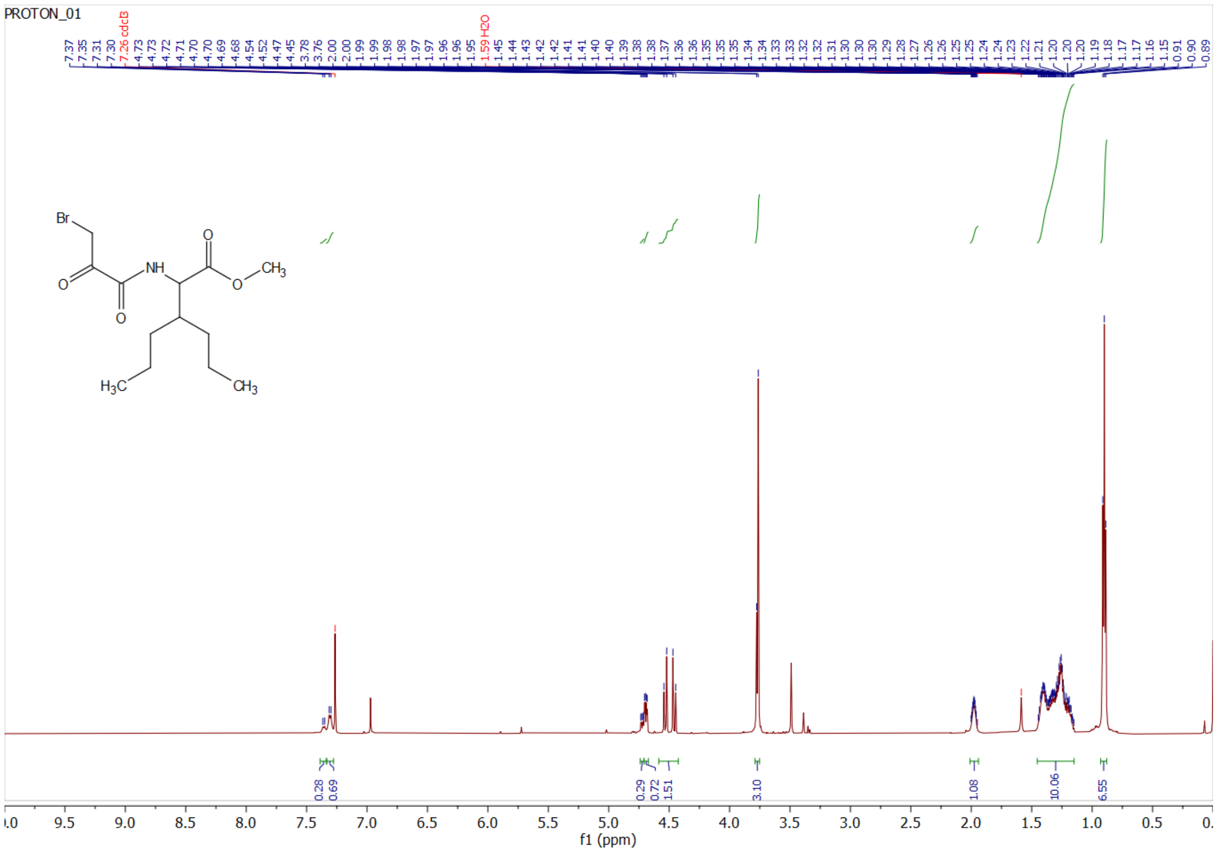

Figure S62. ^{1}H NMR spectrum of compound **18a**.

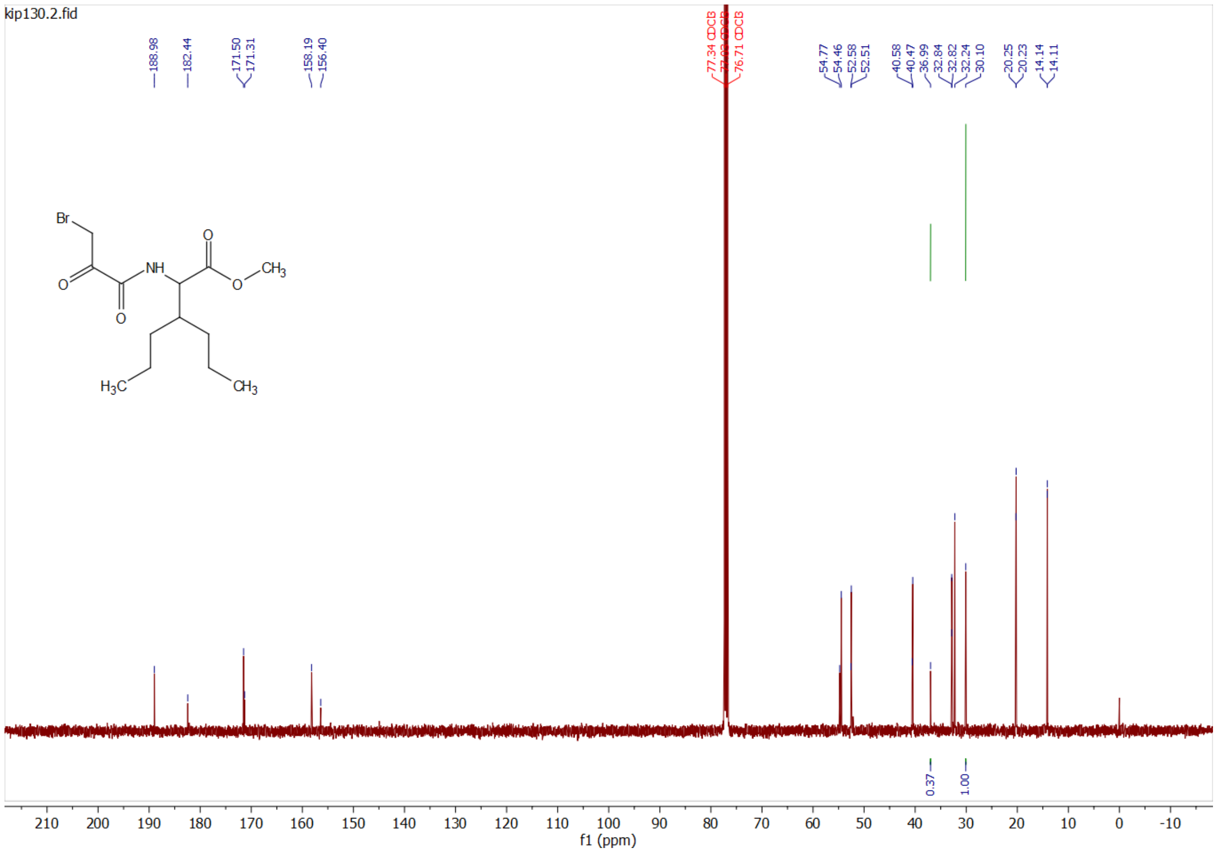

Figure S63. ^{13}C NMR spectrum of compound **18a**.

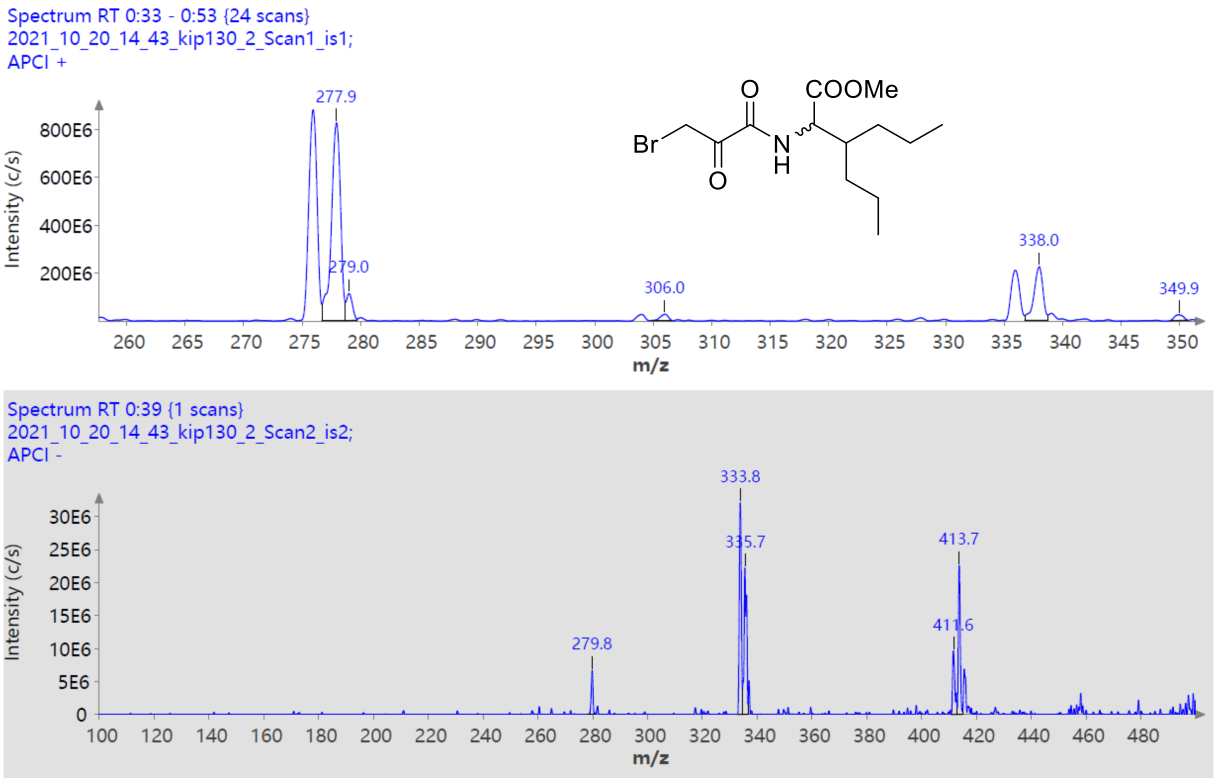

Figure S63. MS spectrum of compound **18a**.

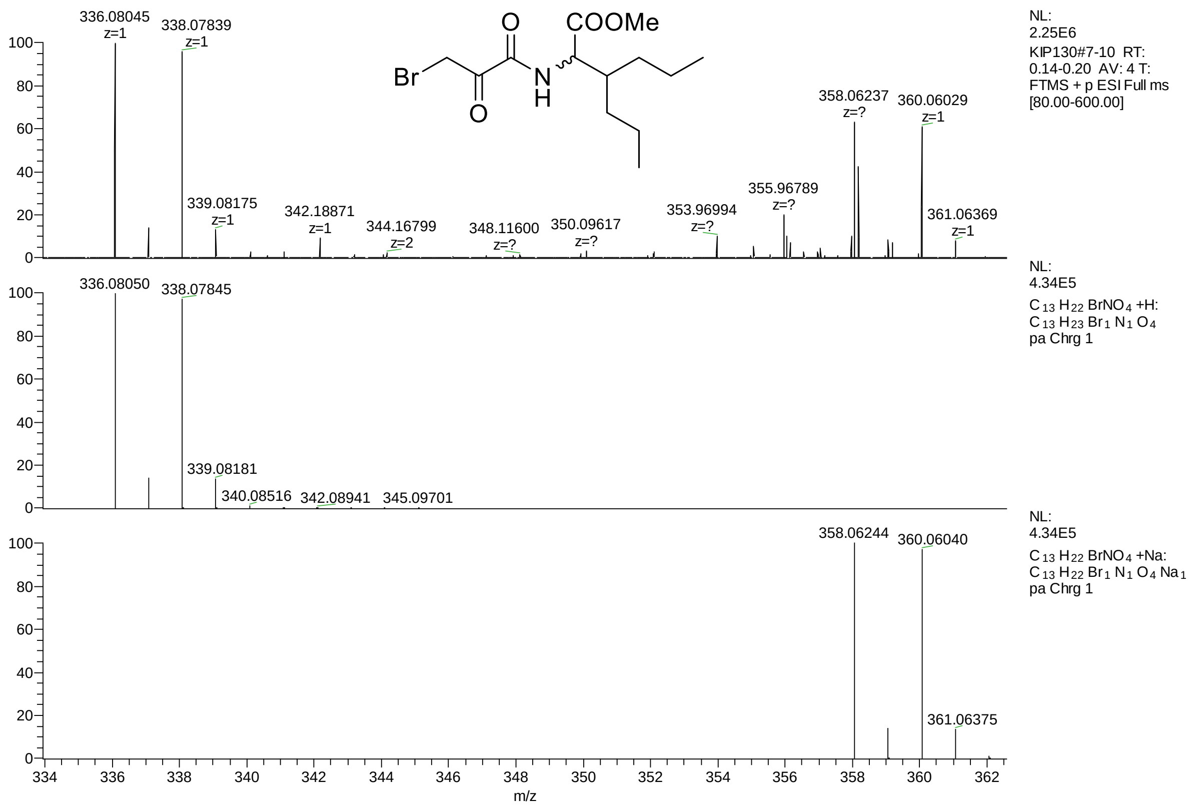

Figure S65. HRMS spectrum of compound **18a**.

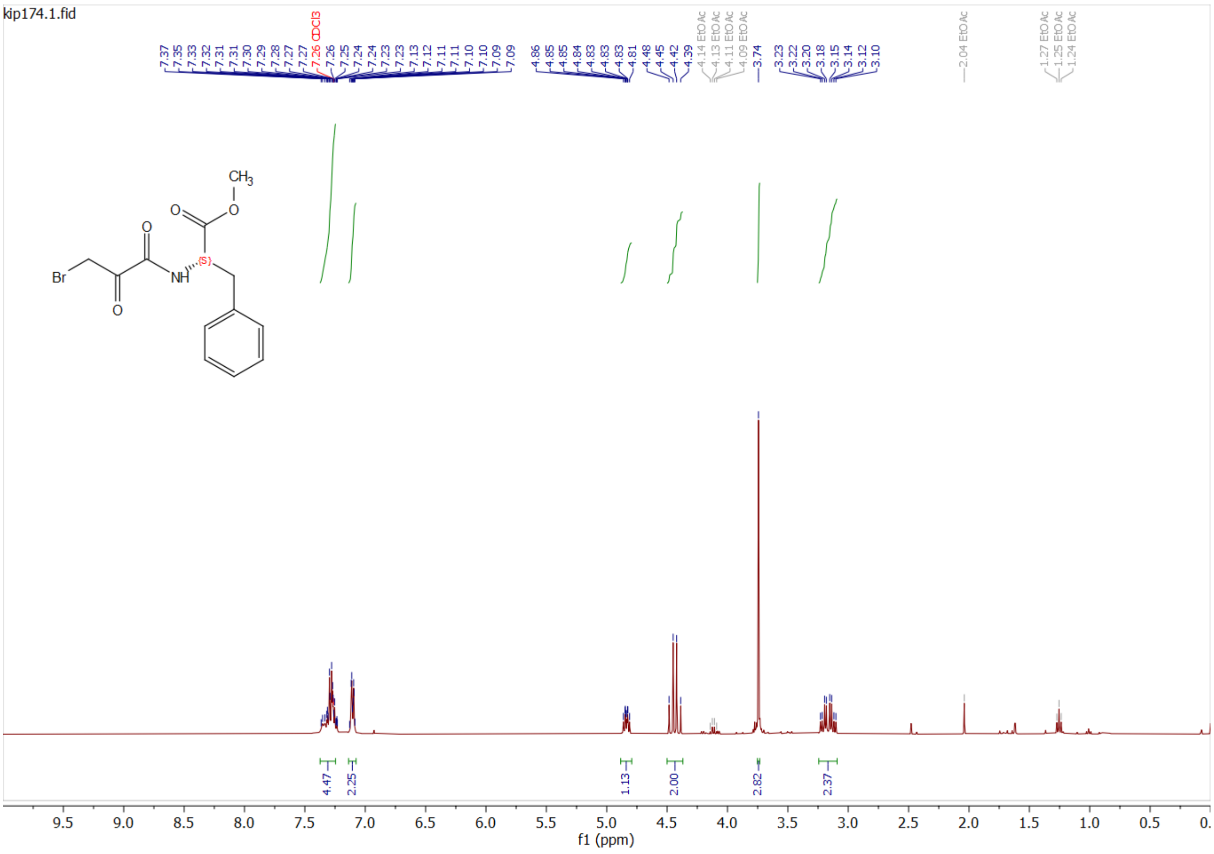

Figure S66. ^{1}H NMR spectrum of compound **18b**.

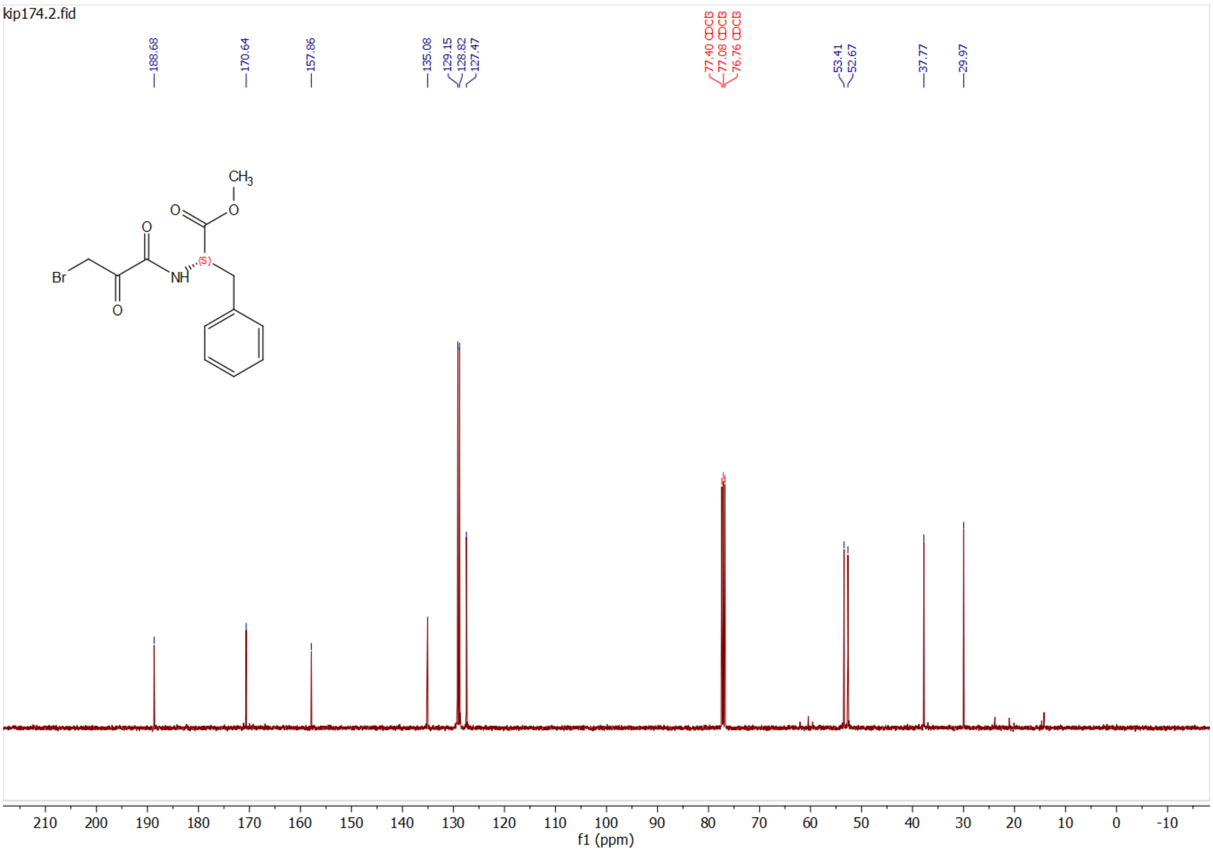

Figure S67. ^{13}C NMR spectrum of compound **18b**.

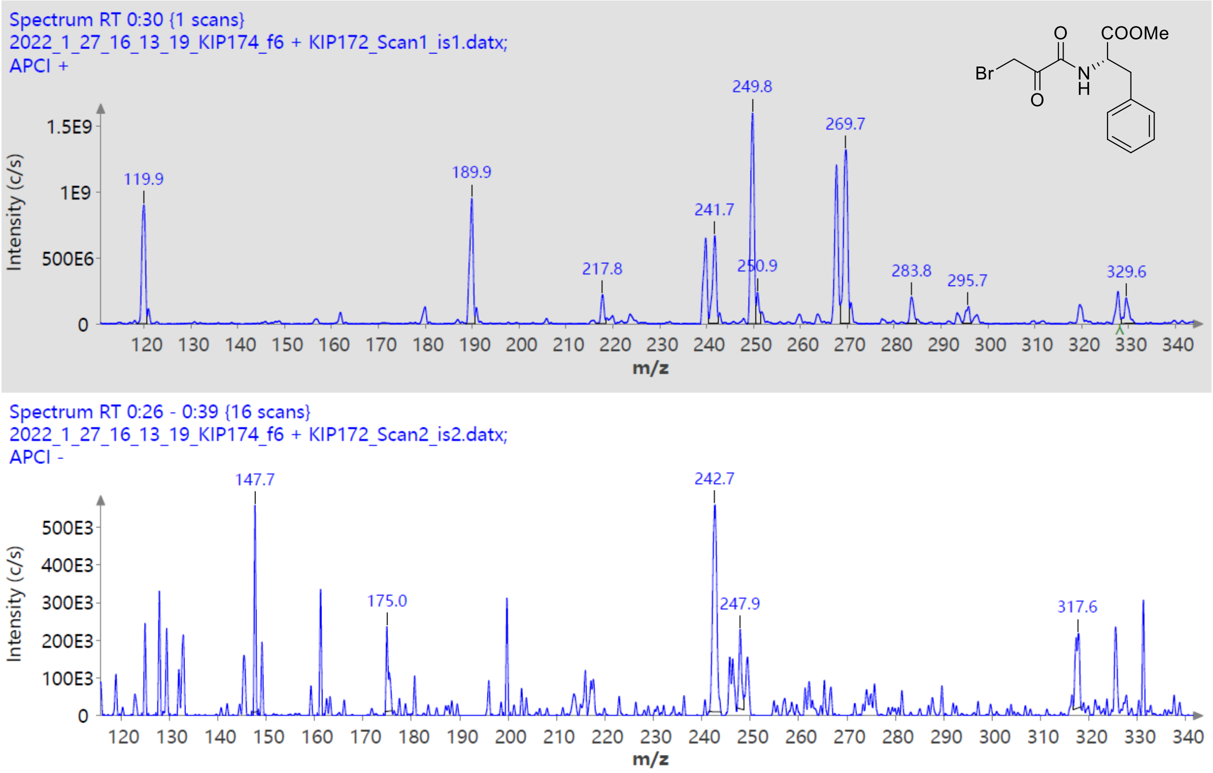

Figure S68. MS spectrum of compound **18b**.

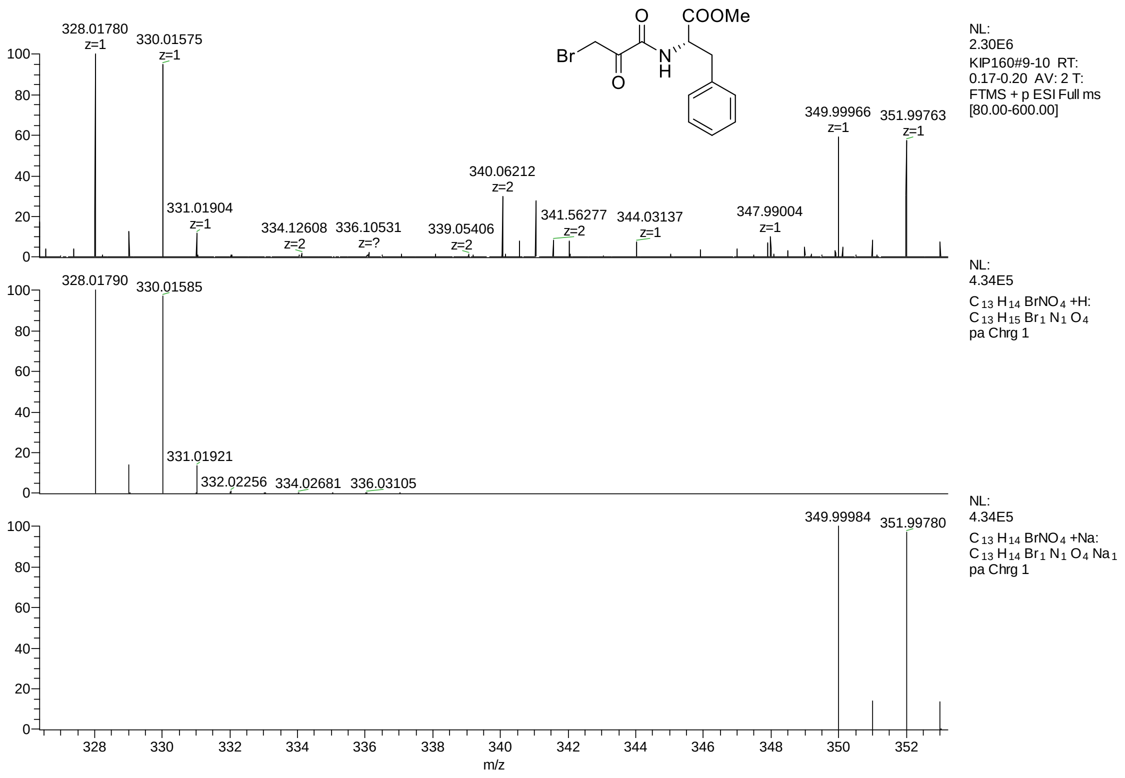

Figure S69. HRMS spectrum of compound **18b**.

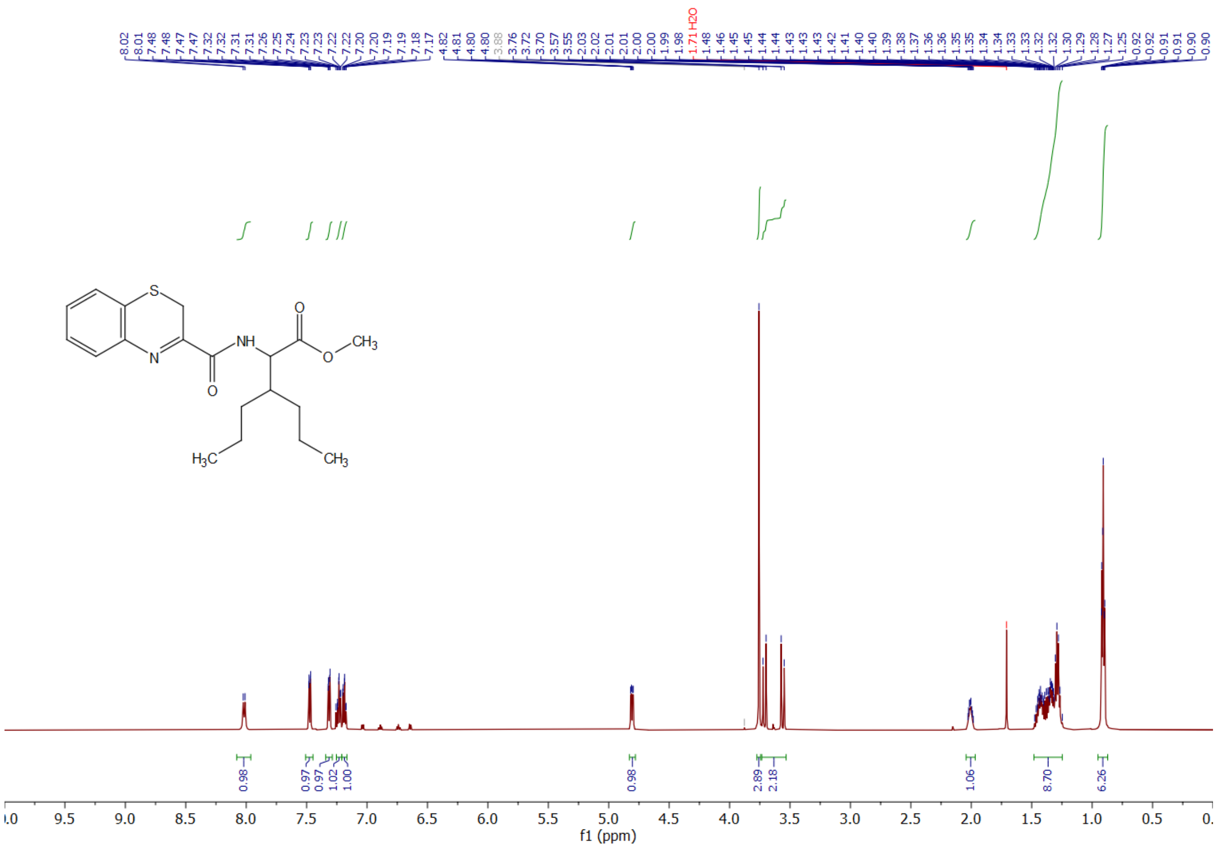

Figure S70. ^{1}H NMR spectrum of compound **19a**.

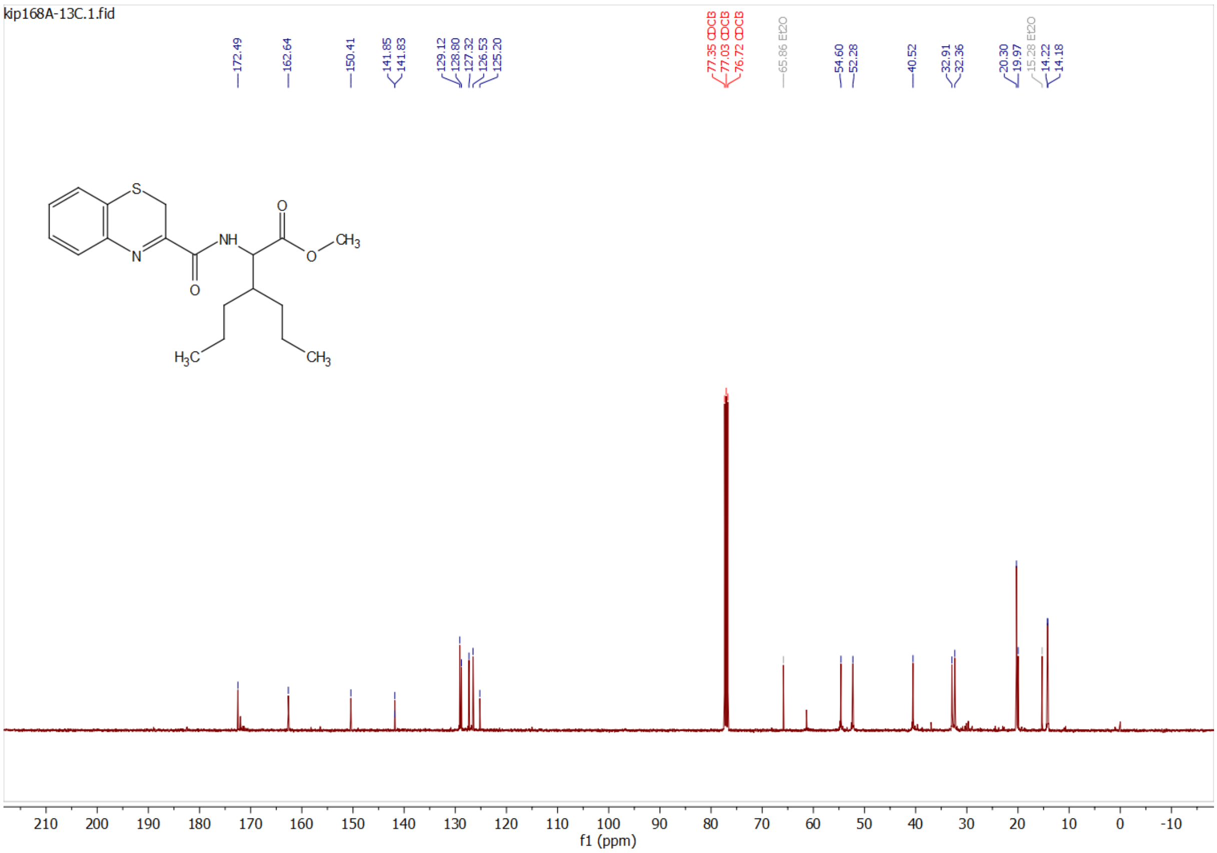

Figure S71. ^{13}C NMR spectrum of compound **19a**.

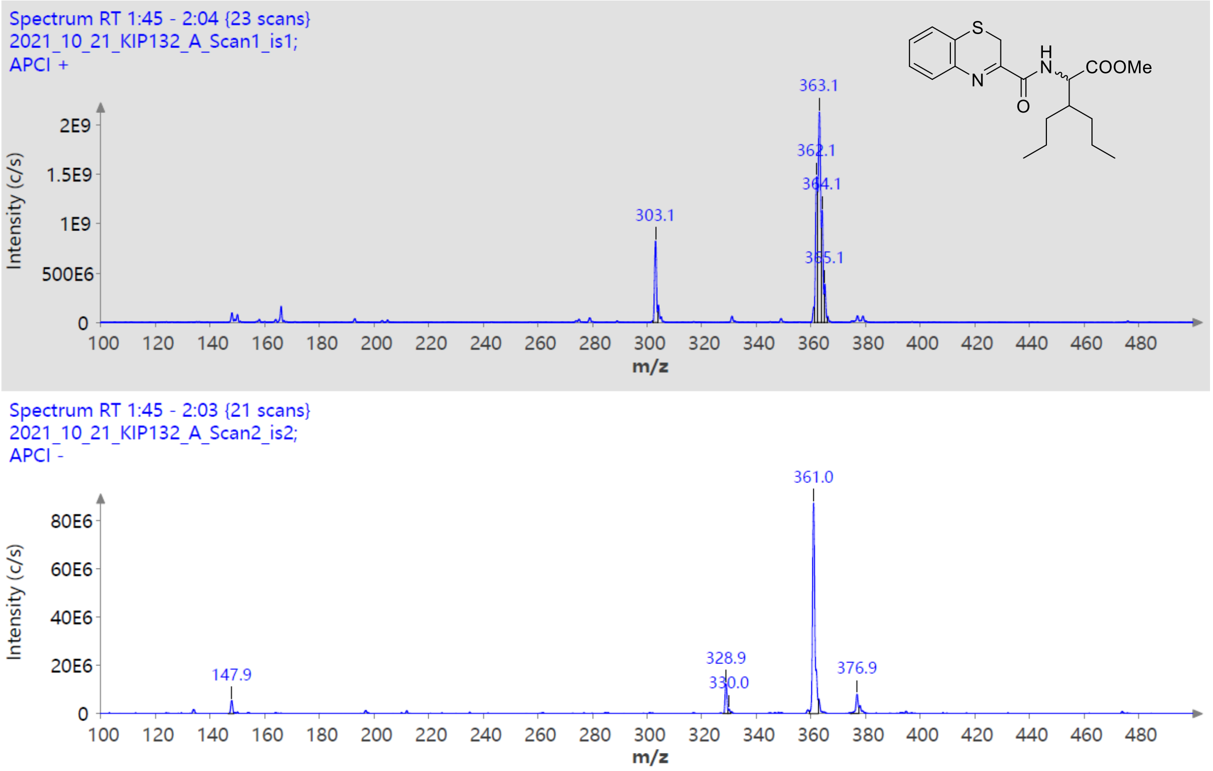

Figure S72. MS spectrum of compound **19a**.

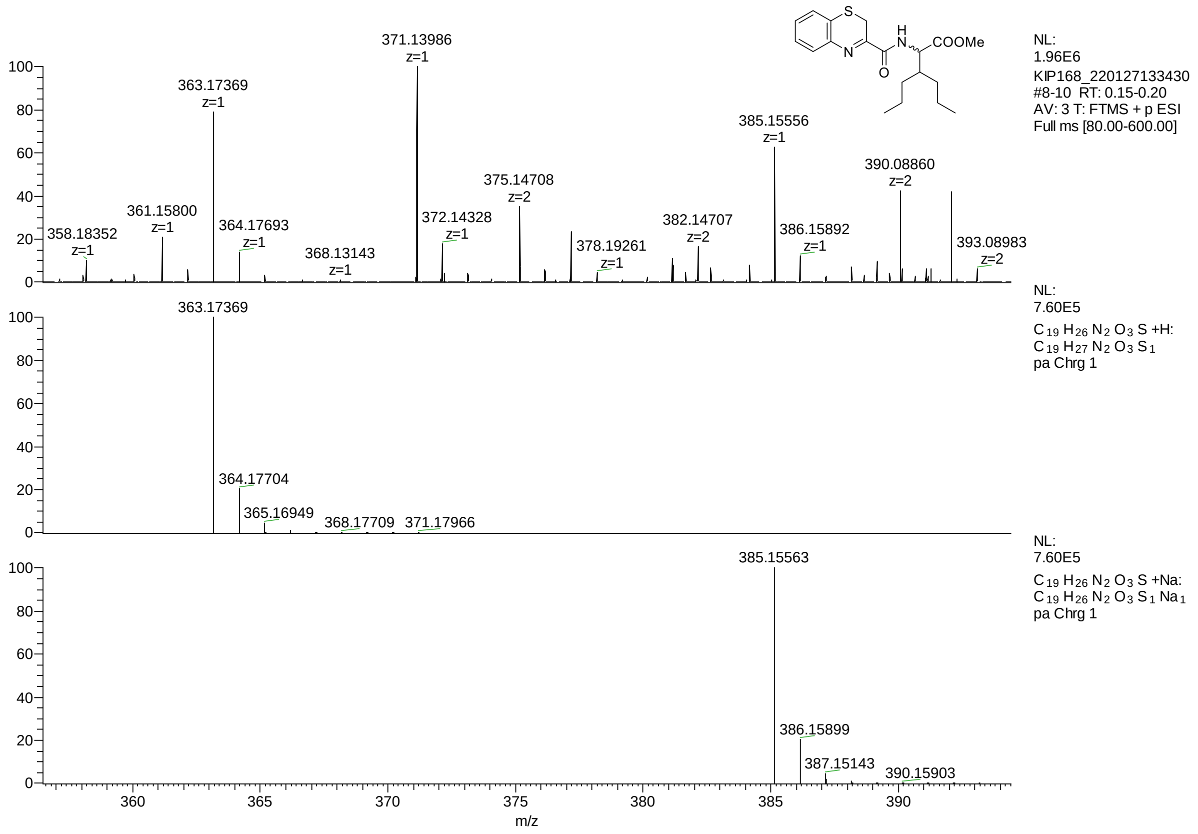

Figure S73. HRMS spectrum of compound **19a**.

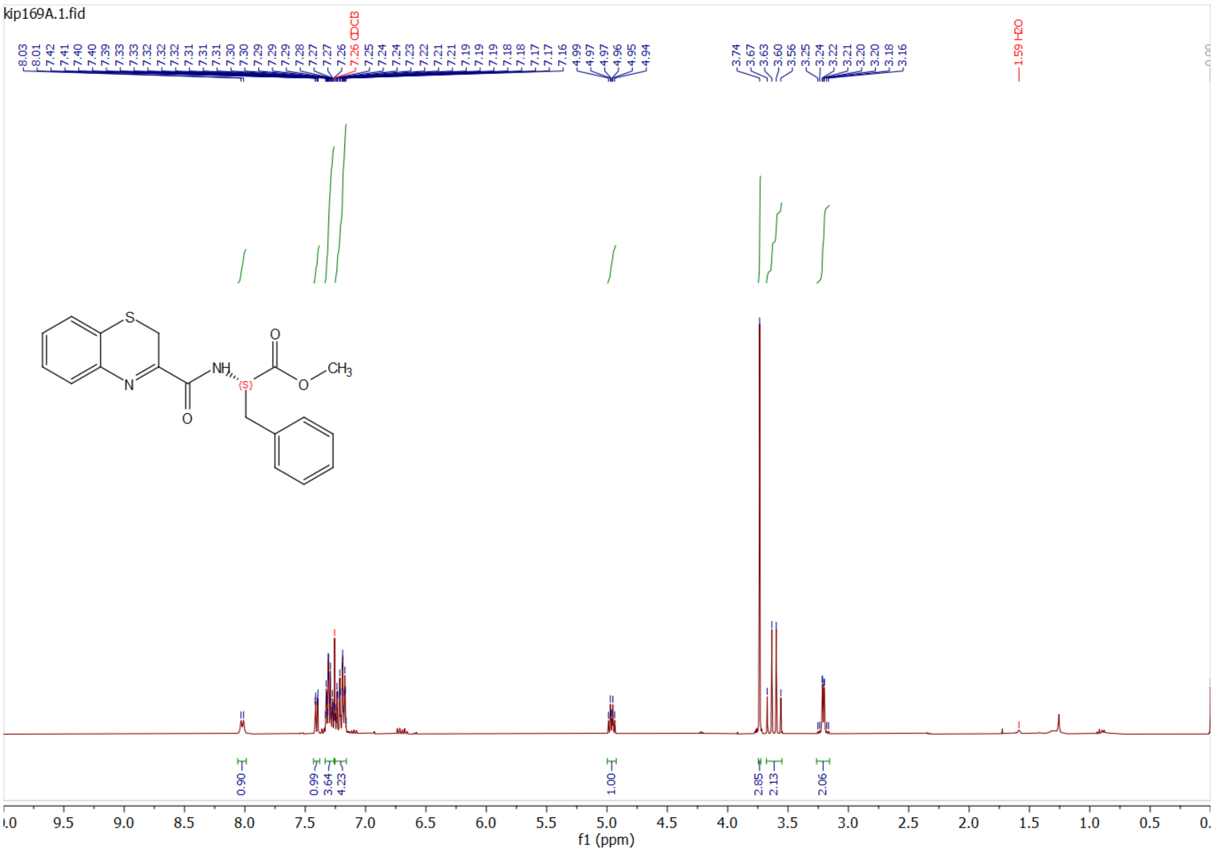

Figure S74. ^{1}H NMR spectrum of compound **19b**.

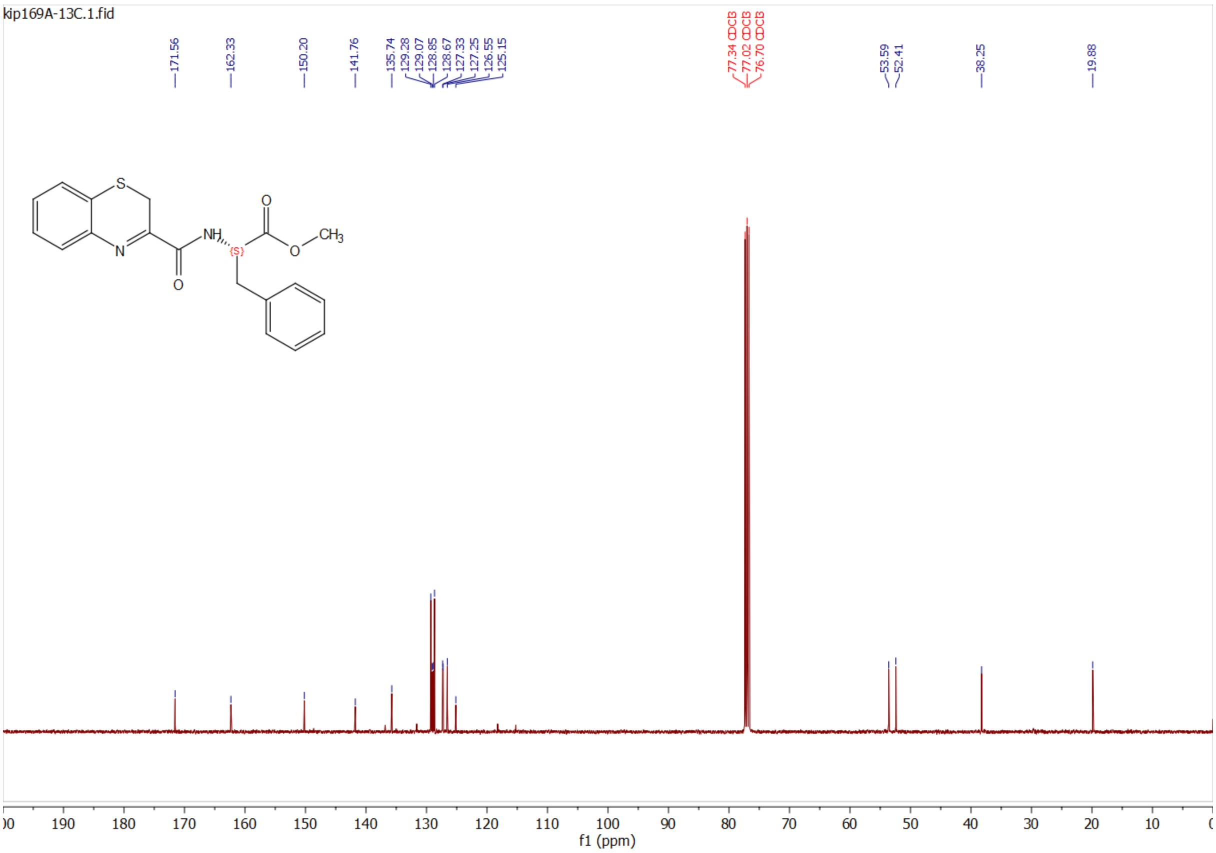

Figure S75. ^{13}C NMR spectrum of compound **19b**.

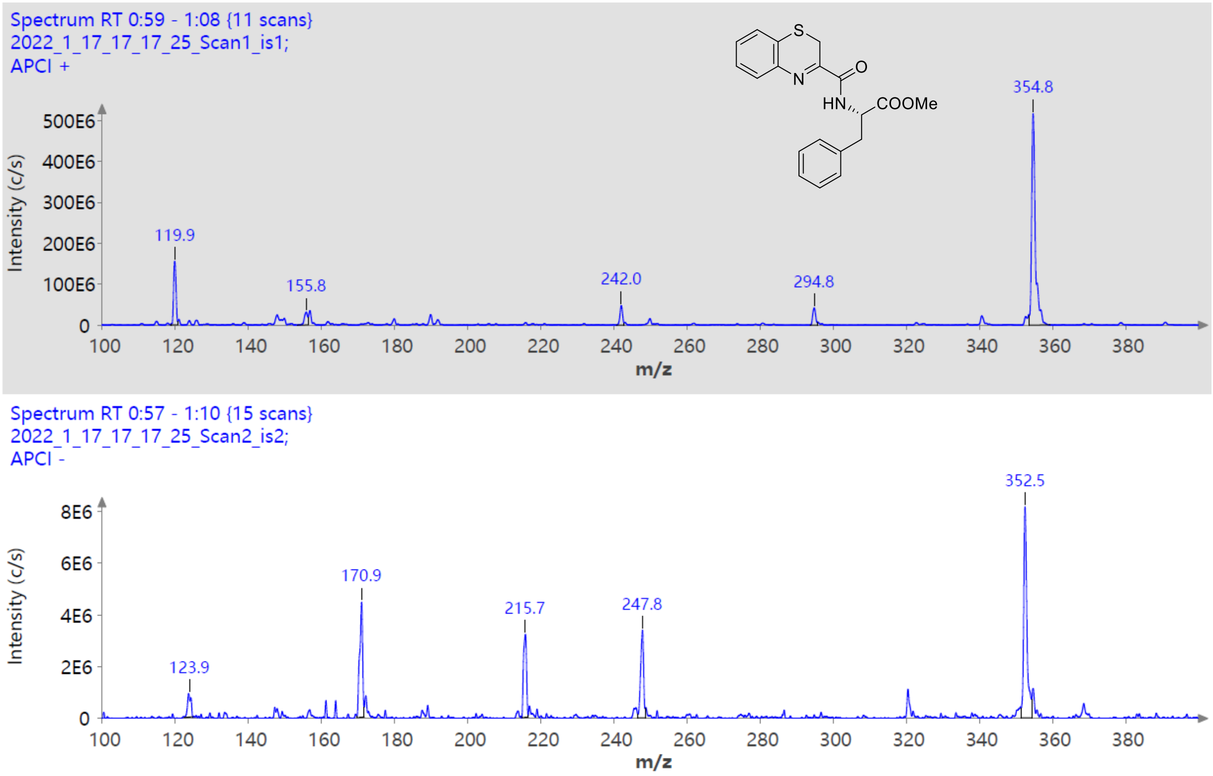

Figure S76. MS spectrum of compound **19b**.

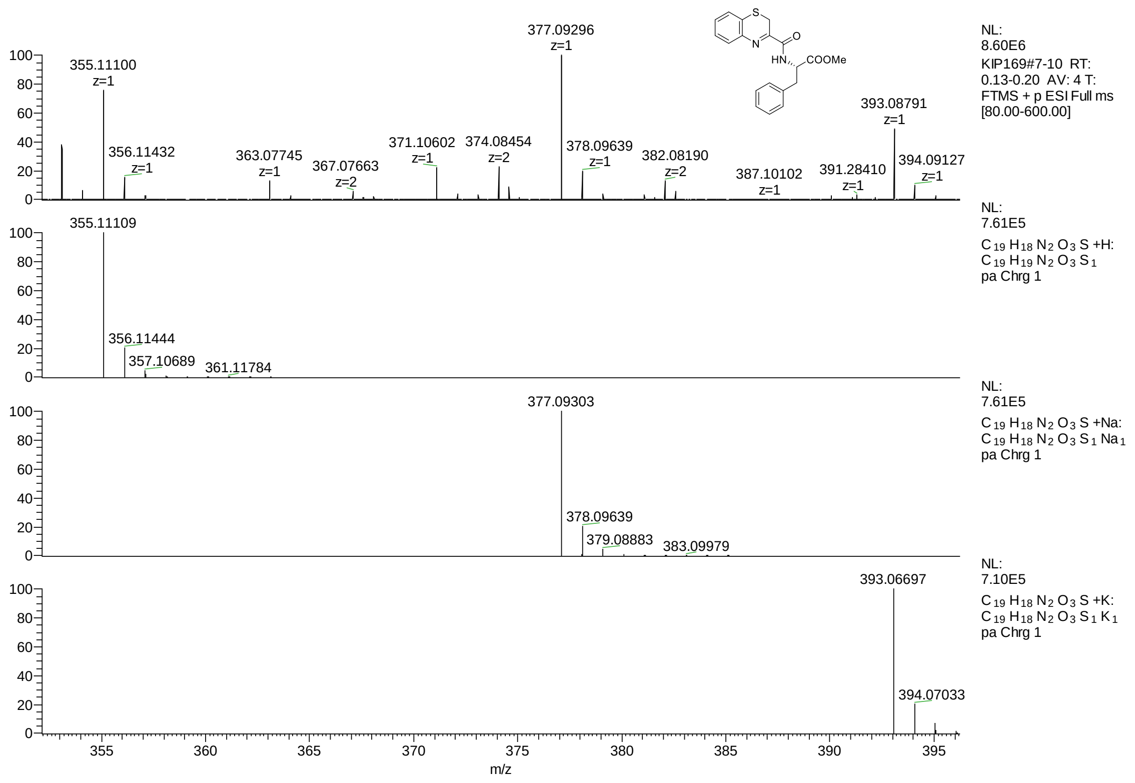

Figure S77. HRMS spectrum of compound **19b**.

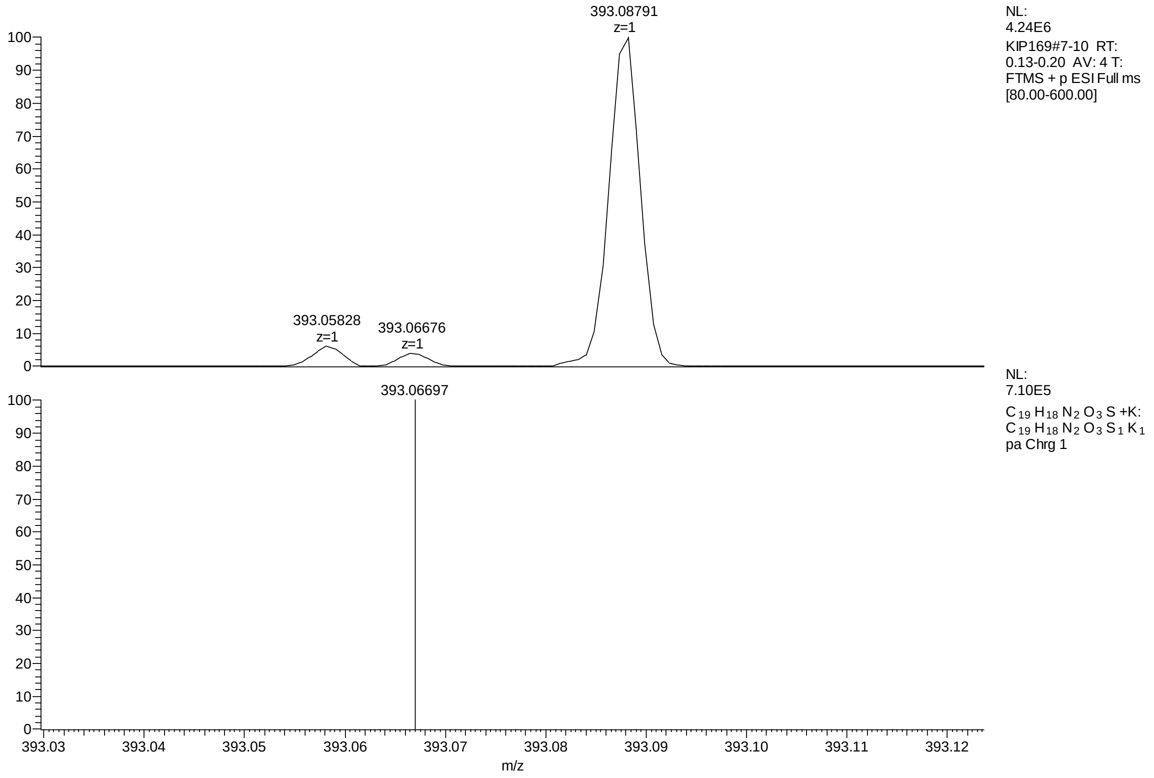

Figure S78. HRMS spectrum of compound **19b** (enlarged for $[M+K]^+$).

6. Stereochemical description of 16·HCl

In the literature [2], the optical rotation of a highly similar deprotected ester is given. Thus, the product of the stereoselective reduction was deprotected using AcCl, and then the optical rotation was measured and compared with the literature analogue.

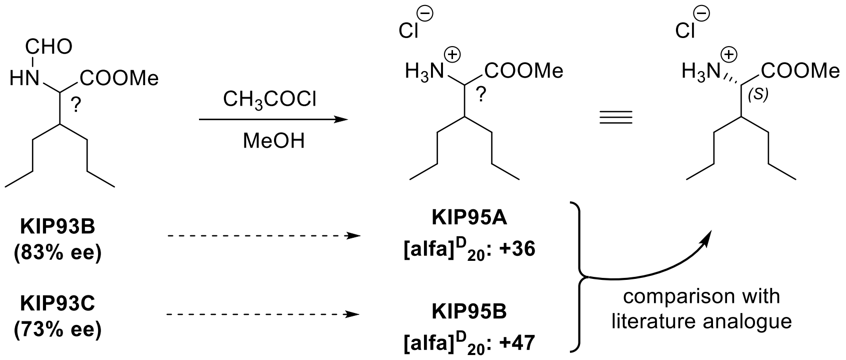

Purified by flash chromatography (SiO_2, CH_2Cl_2 with up to 5% MeOH). Optical rotation measured in MeOH (c = 0.35 and 0.31, respectively)

7. Details of DFT calculations

Structures were built and preoptimized using a semiempirical RM1 method and by HF/3-21G using Spartan program package [3]. Then, the structures were fully geometrically optimized at the wB97xD/6-31G(d) level [4]. Single-point energies were calculated at the MN15/6-311+G(2d,p) level [5]. DFT calculations were performed using the Gaussian program package [6].

Benzothiazine-imine-dimer

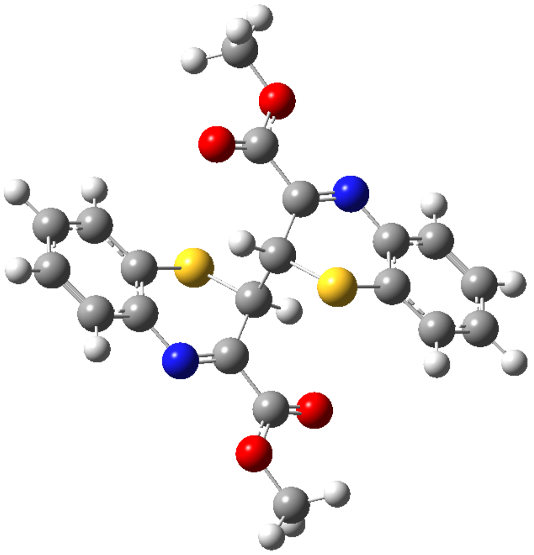

```
Imaginary Freq 0
Temperature    298.15     Kelvin
Pressure  1     atm
Frequencies scaled by    1
Electronic Energy (EE)   -1978.1239      Hartree
Zero-point Energy Correction  0.333555   Hartree
Thermal Correction to Energy  0.357944   Hartree
Thermal Correction to Enthalpy     0.358888   Hartree
Thermal Correction to Free Energy  0.277595   Hartree
EE + Zero-point Energy   -1977.7904      Hartree
EE + Thermal Energy Correction     -1977.766   Hartree
EE + Thermal Enthalpy Correction   -1977.7651      Hartree
EE + Thermal Free Energy Correction     -1977.8463
Hartree
E (Thermal)    224.613    kcal/mol
Heat Capacity (Cv)  91.871     cal/mol-kelvin
Entropy (S)    171.096    cal/mol-kelvin
Energy (FREE) = -5.192835576172E6 kJ.mol-1

Single point energy MN15/6-311+G(2d,p)
E(RMN15)  -1977.3584      Hartree
```

Cartesian coordinates

	Atom	X	Y	Z
1	C	-0.0654	0.7068	-3.9573
2	C	2.1731	-0.8965	-3.5436
3	C	0.5587	0.7039	-2.7105
4	C	0.4210	-0.0929	-4.9827
5	C	1.5355	-0.9059	-4.7738
6	C	1.6955	-0.0985	-2.4978
7	N	2.4394	-0.0881	-1.3103
8	C	1.9017	0.2841	-0.2158
9	C	0.4389	0.6352	-0.0378
10	S	-0.0502	1.7272	-1.4062
11	C	2.7551	0.3923	1.0215
12	O	2.3672	0.9379	2.0317
13	O	3.9538	-0.1624	0.8789
14	C	4.7960	-0.0801	2.0277
15	H	-0.9498	1.3171	-4.1106
16	H	-0.0793	-0.0909	-5.9460
17	H	1.9094	-1.5363	-5.5740
18	H	3.0560	-1.4992	-3.3571
19	H	4.3309	-0.5835	2.8791
20	H	4.9856	0.9634	2.2908
21	H	5.7221	-0.5794	1.7459
22	H	0.3259	1.2216	0.8769
23	C	0.0654	-0.7068	3.9573
24	C	-2.1731	0.8965	3.5436
25	C	-0.5587	-0.7039	2.7105
26	C	-0.4210	0.0929	4.9827
27	C	-1.5355	0.9059	4.7738
28	C	-1.6955	0.0985	2.4978
29	N	-2.4394	0.0881	1.3103
30	C	-1.9017	-0.2841	0.2158
31	C	-0.4389	-0.6352	0.0378
32	S	0.0502	-1.7272	1.4062
33	C	-2.7551	-0.3923	-1.0215
34	O	-2.3672	-0.9379	-2.0317
35	O	-3.9538	0.1624	-0.8789
36	C	-4.7960	0.0801	-2.0277
37	H	0.9498	-1.3171	4.1106
38	H	0.0793	0.0909	5.9460
39	H	-1.9094	1.5363	5.5740
40	H	-3.0560	1.4992	3.3571
41	H	-0.3259	-1.2216	-0.8769
42	H	-4.9856	-0.9634	-2.2908
43	H	-4.3309	0.5835	-2.8791
44	H	-5.7221	0.5794	-1.7459

Benzothiazine-enamine-dimer

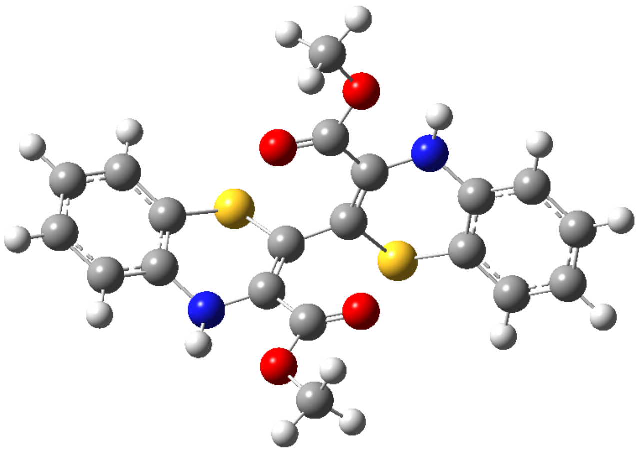

Imaginary Freq 0
Temperature 298.15 Kelvin
Pressure 1 atm
Frequencies scaled by 1
Electronic Energy (EE) -1978.1133 Hartree
Zero-point Energy Correction 0.333367 Hartree
Thermal Correction to Energy 0.358322 Hartree
Thermal Correction to Enthalpy 0.359266 Hartree
Thermal Correction to Free Energy 0.276511 Hartree
EE + Zero-point Energy -1977.7799 Hartree
EE + Thermal Energy Correction -1977.755 Hartree
EE + Thermal Enthalpy Correction -1977.754 Hartree
EE + Thermal Free Energy Correction -1977.8368
Hartree
E (Thermal) 224.85 kcal/mol
Heat Capacity (Cv) 93.939 cal/mol-kelvin
Entropy (S) 174.171 cal/mol-kelvin
Energy (FREE) = -5.1928104422605E6 kJ.mol-1

Single point energy MN15/6-311+G(2d,p)
E(RMN15) -1977.3563 Hartree

Cartesian coordinates

	Atom	X	Y	Z
1	C	-4.5475	0.5321	-1.2239
2	C	-4.5355	0.4879	1.5573
3	C	-3.5670	-0.1802	-0.5450
4	C	-5.5520	1.1890	-0.5163
5	C	-5.5456	1.1577	0.8736
6	C	-3.5435	-0.1918	0.8522
7	N	-2.5211	-0.8849	1.5189
8	C	-1.2182	-0.8756	0.9993
9	C	-0.9838	-0.9518	-0.3246
10	S	-2.3599	-1.1357	-1.4350
11	C	-0.1064	-0.7244	1.9757
12	O	1.0721	-0.6817	1.7144

13	O	-0.5800	-0.6530	3.2380
14	C	0.4155	-0.5329	4.2530
15	H	-4.5335	0.5561	-2.3093
16	H	-6.3313	1.7216	-1.0515
17	H	-6.3216	1.6684	1.4354
18	H	-4.5168	0.4861	2.6443
19	H	-2.5415	-0.7946	2.5246
20	H	0.9949	0.3823	4.1116
21	H	1.0914	-1.3906	4.2270
22	H	-0.1290	-0.5008	5.1962
23	C	3.7807	-2.8398	-0.3197
24	C	4.5451	-0.4588	-1.5379
25	C	2.8291	-2.0407	-0.9406
26	C	5.1249	-2.4742	-0.3434
27	C	5.5027	-1.2868	-0.9599
28	C	3.2026	-0.8341	-1.5384
29	N	2.2197	-0.0221	-2.1256
30	C	0.9572	0.0900	-1.5248
31	C	0.3457	-0.9686	-0.9600
32	S	1.1315	-2.5613	-1.0380
33	C	0.3460	1.4457	-1.4932
34	O	-0.7218	1.7362	-1.0091
35	O	1.1432	2.3479	-2.1042
36	C	0.6297	3.6784	-2.1491
37	H	3.4691	-3.7592	0.1667
38	H	6.5469	-0.9904	-0.9818
39	H	4.8385	0.4821	-1.9967
40	H	2.5627	0.8584	-2.4818
41	H	0.4824	4.0638	-1.1376
42	H	-0.3237	3.7009	-2.6817
43	H	1.3798	4.2651	-2.6782
44	H	5.8682	-3.1151	0.1194

Benzothiazine-imine-Phe

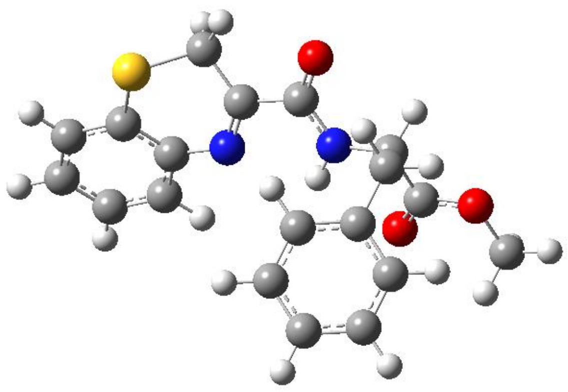

Imaginary Freq 0
Temperature 298.15 Kelvin
Pressure 1 atm
Frequencies scaled by 1

Electronic Energy (EE) -1467.899 Hartree
Zero-point Energy Correction 0.344768 Hartree
Thermal Correction to Energy 0.366783 Hartree
Thermal Correction to Enthalpy 0.367727 Hartree
Thermal Correction to Free Energy 0.289259 Hartree
EE + Zero-point Energy -1467.5542 Hartree
EE + Thermal Energy Correction -1467.5322 Hartree
EE + Thermal Enthalpy Correction -1467.5313 Hartree
EE + Thermal Free Energy Correction -1467.6097
Hartree
E (Thermal) 230.16 kcal/mol
Heat Capacity (Cv) 83.421 cal/mol-kelvin
Entropy (S) 165.15 cal/mol-kelvin
Energy (FREE) = -3.8532093224855E6 kJ.mol-1

Cartesian coordinates

	Atom	X	Y	Z
1	H	1.0479	4.4209	-4.5328
2	C	0.2794	3.9707	-3.9114
3	C	-1.6956	2.8089	-2.3333
4	C	0.4748	2.6869	-3.4013
5	C	-0.8987	4.6585	-3.6474
6	C	-1.8955	4.0725	-2.8678
7	C	-0.5161	2.1030	-2.5895
8	H	-1.0411	5.6534	-4.0579
9	H	-2.8183	4.6071	-2.6667
10	H	-2.4394	2.3395	-1.6974
11	N	-0.3500	0.8712	-1.9377
12	C	0.4822	0.0104	-2.3783
13	C	1.2904	0.1511	-3.6395
14	S	1.9853	1.8232	-3.7199
15	H	2.1087	-0.5697	-3.6346
16	C	0.6809	-1.2629	-1.5811
17	O	1.5256	-2.0891	-1.9085
18	N	-0.1285	-1.3801	-0.5130
19	H	-0.7808	-0.6309	-0.3115
20	C	-0.0171	-2.4773	0.4127
21	H	0.0451	-3.4156	-0.1501
22	C	-1.2809	-2.4927	1.2557
23	O	-2.1479	-1.6534	1.2106
24	O	-1.2977	-3.5451	2.0800
25	C	-2.4077	-3.5950	2.9789
26	H	-2.2630	-4.4967	3.5728
27	H	-2.4149	-2.7083	3.6175
28	H	-3.3475	-3.6458	2.4246
29	C	1.2454	-2.3762	1.3077
30	H	2.1049	-2.3554	0.6316
31	H	0.6592	-0.0369	-4.5159
32	H	1.3136	-3.2916	1.9028
33	C	1.2240	-1.1604	2.1999
34	C	1.0664	1.1219	3.8220

35	C	1.6436	0.0839	1.7224
36	C	0.7308	-1.2444	3.5046
37	C	0.6524	-0.1134	4.3116
38	C	1.5639	1.2172	2.5252
39	H	2.0381	0.1653	0.7131
40	H	0.4088	-2.2090	3.8903
41	H	0.2703	-0.1979	5.3248
42	H	1.8931	2.1763	2.1365
43	H	1.0058	2.0060	4.4497

Benzothiazine-imine-norleucine

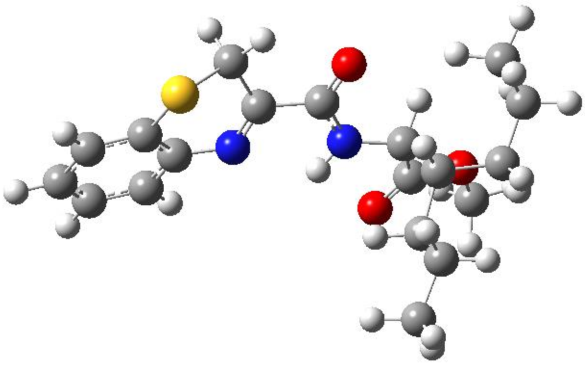

Imaginary Freq 0
Temperature 298.15 Kelvin
Pressure 1 atm
Frequencies scaled by 1
Electronic Energy (EE) -1472.744 Hartree
Zero-point Energy Correction 0.435646 Hartree
Thermal Correction to Energy 0.461386 Hartree
Thermal Correction to Enthalpy 0.46233 Hartree
Thermal Correction to Free Energy 0.376848 Hartree
EE + Zero-point Energy -1472.3083 Hartree
EE + Thermal Energy Correction -1472.2826 Hartree
EE + Thermal Enthalpy Correction -1472.2817 Hartree
EE + Thermal Free Energy Correction -1472.3671
Hartree
E (Thermal) 289.524 kcal/mol
Heat Capacity (Cv) 94.884 cal/mol-kelvin
Entropy (S) 179.913 cal/mol-kelvin
Energy (FREE) = -3.865699941823E6 kJ.mol-1

Cartesian coordinates

	Atom	X	Y	Z
1	H	1.8542	4.7238	-5.0486
2	C	0.9641	4.1959	-4.7193
3	C	-1.3133	2.8319	-3.8881
4	C	1.0949	3.0863	-3.8836
5	C	-0.2921	4.6085	-5.1462
6	C	-1.4341	3.9181	-4.7415
7	C	-0.0569	2.4038	-3.4487

8	H	-0.3785	5.4687	-5.8029
9	H	-2.4149	4.2362	-5.0799
10	H	-2.1857	2.2932	-3.5319
11	N	-0.0260	1.3545	-2.5167
12	C	1.0355	0.6678	-2.3457
13	C	2.3044	0.8187	-3.1403
14	S	2.6939	2.5793	-3.3226
15	H	3.1270	0.3292	-2.6181
16	C	1.0266	-0.4093	-1.2794
17	O	2.0309	-1.0767	-1.0526
18	N	-0.1492	-0.5498	-0.6434
19	H	-0.9240	0.0420	-0.9207
20	C	-0.3477	-1.5252	0.4032
21	H	0.1417	-2.4559	0.1022
22	C	-1.8423	-1.7652	0.5069
23	O	-2.6857	-1.0073	0.0850
24	O	-2.1185	-2.9040	1.1477
25	C	-3.5071	-3.1725	1.3544
26	H	-3.5440	-4.1180	1.8936
27	H	-3.9659	-2.3748	1.9432
28	H	-4.0260	-3.2544	0.3967
29	C	0.2679	-1.0519	1.7530
30	H	1.2929	-0.7657	1.4857
31	C	0.3481	-2.1596	2.8151
32	H	0.9165	-1.7579	3.6633
33	H	-0.6588	-2.3767	3.1928
34	C	-0.4528	0.2003	2.2725
35	H	-1.4592	-0.0699	2.6258
36	H	-0.5984	0.8978	1.4376
37	C	1.0140	-3.4688	2.3745
38	H	0.3793	-3.9825	1.6421
39	H	1.0555	-4.1332	3.2466
40	C	0.2890	0.9366	3.3897
41	H	0.3643	0.2990	4.2786
42	H	1.3183	1.1390	3.0648
43	C	-0.3956	2.2482	3.7707
44	H	0.1428	2.7601	4.5750
45	H	-1.4214	2.0703	4.1136
46	H	-0.4459	2.9286	2.9131
47	C	2.4231	-3.2871	1.8081
48	H	2.4203	-2.7149	0.8741
49	H	2.8883	-4.2571	1.6036
50	H	3.0629	-2.7547	2.5226
51	H	2.1888	0.3540	-4.1265

8. References

1 K. Tani, K. Suwa, E. Tanigawa, T. Ise, T. Yamagata, Y. Tatsuno, S. Otsuka, *J. Organomet. Chem.* **1989**, *370*, 203-221.

2 WO/2006/026759, M. Dennis, S., S. Fong, Humanized anti-beta7 antagonists and uses therefor.

3 *Spartan ´20*, Wavefunction, Inc., Irvine, CA,

4 J.-D. Chai, M. Head-Gordon, *Phys. Chem. Chem. Phys.* **2008**, *10*, 6615-6620.

5 H. S. Yu, X. He, S. L. Li, D. G. Truhlar, *Chem. Sci.* **2016**, *7*, 5032-5051.

6 *Gaussian 16 Rev. C.01*, M. J. Frisch, G. W. Trucks, H. B. Schlegel, G. E. Scuseria, M. A. Robb, J. R. Cheeseman, G. Scalmani, V. Barone, G. A. Petersson, H. Nakatsuji, X. Li, M. Caricato, A. V. Marenich, J. Bloino, B. G. Janesko, R. Gomperts, B. Mennucci, H. P. Hratchian, J. V. Ortiz, A. F. Izmaylov, J. L. Sonnenberg, Williams, F. Ding, F. Lipparini, F. Egidi, J. Goings, B. Peng, A. Petrone, T. Henderson, D. Ranasinghe, V. G. Zakrzewski, J. Gao, N. Rega, G. Zheng, W. Liang, M. Hada, M. Ehara, K. Toyota, R. Fukuda, J. Hasegawa, M. Ishida, T. Nakajima, Y. Honda, O. Kitao, H. Nakai, T. Vreven, K. Throssell, J. A. Montgomery Jr., J. E. Peralta, F. Ogliaro, M. J. Bearpark, J. J. Heyd, E. N. Brothers, K. N. Kudin, V. N. Staroverov, T. A. Keith, R. Kobayashi, J. Normand, K. Raghavachari, A. P. Rendell, J. C. Burant, S. S. Iyengar, J. Tomasi, M. Cossi, J. M. Millam, M. Klene, C. Adamo, R. Cammi, J. W. Ochterski, R. L. Martin, K. Morokuma, O. Farkas, J. B. Foresman, D. J. Fox, Wallingford, CT, **2016**.
